# Influence of Pollen Dispersal and Mating Pattern in Domestication of Intermediate Wheatgrass, a Novel Perennial Food Crop

**DOI:** 10.3389/fpls.2022.871130

**Published:** 2022-04-28

**Authors:** Prabin Bajgain, Yaniv Brandvain, James A. Anderson

**Affiliations:** ^1^Department of Agronomy and Plant Genetics, University of Minnesota, Saint Paul, MN, United States; ^2^Department of Plant Biology, University of Minnesota, Saint Paul, MN, United States

**Keywords:** intermediate wheatgrass, genetic diversity, perennial, pollen, domestication, breeding

## Abstract

Intermediate wheatgrass (IWG) is a perennial forage grass that is currently being domesticated as a grain crop. It is a primarily wind-pollinated outcrossing species and expresses severe inbreeding depression when self-pollinated. Characterization of pollen dispersal, mating parameters, and change in genetic diversity due to pollen movement is currently lacking in IWG. In this study, we examined pollen dispersal in an IWG selection nursery by evaluating 846 progeny from 15 mother plants and traced their parentage to 374 fathers. A set of 2,500 genomic loci was used to characterize the population. We assigned paternity to 769 (91%) progeny and the average number of fathers per mother plant was 37, from an average of 56 progeny examined per mother. An extensive number (80%) of pollination events occurred within 10 m of the mother plants. Pollination success was not correlated with trait attributes of the paternal genotypes. Mating system analysis confirmed that IWG is highly outcrossing and inbreeding was virtually absent. Neither genetic diversity nor the genome-estimated trait values of progeny were significantly affected by pollinator distance. The distance of pollinator in an IWG breeding nursery therefore was not found to be a major contributor in maintaining genetic diversity. These findings reveal the pollen dispersal model in IWG for the first time and its effect on genetic diversity, which will be valuable in designing future IWG breeding populations. Information generated and discussed in this study could be applied in understanding gene flow and genetic diversity of other open-pollinated species.

## Introduction

The reproductive success of plants in a nursery can have profound influence on the genetic makeup of succeeding generations as well as on their trait and behavioral expression. A better understanding of pollen movement is therefore important in outcrossing plant populations, especially so in breeding populations, as high genetic diversity is desired for population maintenance and variety development.

Genetic diversity of plant populations is a key deterministic feature in crop breeding programs. Higher amounts of genetic diversity allows for the selection of individuals with specific gene combinations that favor trait improvement. Genetic diversity of a population is typically determined by mutation, genetic drift, gene flow, and selection (Falconer and Mackay, 1996). By distributing genomic variation across a physical landscape, gene flow plays an important role in the distribution of genetic variation (Chybicki and Oleksa, 2018). Specifically, gene flow mediates the operation of genetic drift and selection by setting “Wright’s neighborhood size”; for example, (a) spatially restricted gene flow can limit the spread of adaptive alleles (Kawecki, 2008), (b) decrease the local effective population size (and therefore local genetic diversity; Wright, 1946), and (c) encourage mating between close relatives, and increase population divergence (measured by fixation index, F_ST_, for example) while spatially expansive gene flow can limit local adaptation (Haldane, 1956). Pollen dispersal and fertilization plays an important role in both generating novel genotypic combinations and mediating gene flow (Crawford, 1984; Rieseberg and Burke, 2001).

In the applied evolutionary scenarios of plant breeding and conservation, restricted gene flow or strong selection pressure can ultimately lead to a reduction in genetic diversity. This is a concern for crop and forage restoration projects as well as in crop domestication and breeding programs (Ellstrand and Elam, 1993). Conversely, broader pollen dispersal kernels can foster genetic diversity by facilitating hybridization (Klein et al., 2017) or transgene escape (Hudson et al., 2004). In addition to characterizing the average distance of pollen dispersal, differential pollination success could induce selection on traits unrelated to plant vigor and likely will further decrease genetic diversity. A sound knowledge of genetic diversity, gene flow, mating system, and progeny composition would therefore be of great value to breeding programs to evaluate germplasm and making informed selection decisions.

Parentage analysis is one of the most frequent and reliable methods implemented to assess pollen dispersal in local plant populations (Sork et al., 1999). It uses genotype data from the progeny obtained from selected mother plants with or without genotypic information of the mother plants. Paternity analysis is also used to estimate pollen dispersal, pollination distance between parental pairs, and infer the genetic structure of progeny (Smouse and Sork, 2004; Robledo-Arnuncio et al., 2006). In breeding populations, paternity analysis is carried out to study pollen dispersal and quantify paternal contribution in the progeny makeup as well as any possible contamination of foreign pollen (Nurminiemi et al., 1998). Historically, genotypic data for parentage analysis have come from microsatellite [simple sequence repeat, (SSR)] markers; however, the ease and low cost per data point of SNP-based genotyping provides a high throughput and modern approach to parentage analysis (Flanagan and Jones, 2019).

Limited work has been carried out to study pollen dispersal and gene flow in perennial grass species. Studies that tracked pollen travel in wind-pollinated grass species of the Poaceae family, namely, *Poa pratensis* L., *Agrostis stolonifera* L., *Lolium perenne* L., and *Festuca arundinacea*, found that gene flow varied by species, location, and field size (Wipff and Fricker, 2001; Cunliffe et al., 2004; Wang et al., 2004; Johnson et al., 2006). In all species, the proportion of pollen travel and gene flow was reduced exponentially as pollination distance increased from the recipient plant. To our knowledge, no such investigation has been done in a perennial grain species. As perennial grain crops, such as intermediate wheatgrass, perennial wheat, perennial cereal rye, perennial sorghum, and perennial rice, are increasingly being adopted by breeding institutions and commercial entities (Ryan et al., 2018), obtaining a first-hand knowledge on gene flow and its impact on population diversity will widen our understanding of population genetics in perennial cropping systems.

Intermediate wheatgrass (IWG) [*Thinopyrum intermedium* (Host) Barkworth and D. R. Dewey subsp. *intermedium*, 2n = 6x = 42] is a cool-season perennial grass species that is native to the Mediterranean and Eastern European regions (Tsvelev, 1984). It is predominantly outcrossing as self-pollination results in severe inbreeding depression. IWG was selected as the best candidate for domestication as a perennial grain crop because it had relatively low seed shattering, low lodging, and high threshability compared with other perennial grasses and produced relatively large edible seed with synchronous maturity (Wagoner, 1990). The crop also provides excellent ecosystem services, such as reduced soil erosion and nutrient leaching to groundwater while improving carbon sequestration (Culman et al., 2013; Jungers et al., 2019). The University of Minnesota (UMN) started their IWG breeding and domestication program in 2011 and a few other institutions have also been involved with improving this crop (Cattani, 2016; De Haan et al., 2018; Bajgain et al., 2020b). The IWG breeding and domestication program at UMN combines traditional breeding methods with genomic selection. Genomic selection is a robust statistical selection approach that uses genome-wide markers to predict trait performance of several thousand plants and has potential to improve important agronomic and domestication traits of IWG, such as grain yield, resistance to seed shatter, improved grain threshing, and larger seed size (Meuwissen et al., 2001; Bajgain et al., 2020a). The IWG cultivars released by UMN are population synthetics generated from open pollination of 7–10 parent plants in 5–7 replications (Bajgain et al., 2020b). Crain et al. (2020) used genotyping by sequencing to successfully assign paternity to progeny obtained from multiple breeding cycles of IWG, suggesting that parentage analysis could be used to characterize pollen dispersal and its influence on population diversity in IWG.

This study was conducted with the primary goal of understanding the extent of pollen dispersal in IWG. Specifically, we: (i) studied the parameters and patterns of mating in IWG, (ii) traced the parentage of progeny used to create the next breeding population, (iii) studied patterns of genetic diversity in both parents and progeny, and (iv) evaluated the effect of pollen dispersal in progeny diversity and trait distribution. For this purpose, we characterized several aspects of population properties as functions of pollen dispersal, such as number and distance of paternal genotypes contributing to the progeny, genetic diversity among the progeny, and genomic-predicted trait distribution in the progeny.

## Materials and Methods

### Population Sampling

The intermediate wheatgrass (IWG) population used in this study is the breeding germplasm from the fourth recurrent selection cycle at the University of Minnesota and is referred to as UMN_C4. This population was developed by inter-crossing 73 cycle 3 (UMN_C3; Bajgain et al., 2019) genets selected based on their superior genome-estimated breeding values (GEBVs). A genet is defined as a genetically unique organism and refers to individual plants in an outcrossing species, such as IWG (Zhang et al., 2016). From each of the 73 mother plants (i.e., families), nine random seeds (i.e., half-sibs) were germinated and transplanted in the field in September 2019. In addition, 114 F_1_ genets obtained from 12 bi-parental crosses among eight UMN_C3 genets were transplanted in the same field. The eight UMN_C3 genets were selected because of their relatively large grain size observed in 2017. The site of population establishment is located within the University of Minnesota’s Agricultural Research Station in St. Paul, MN, USA (44° 59′ 36.20″ N, −93° 10′ 28.90″ W). Each seedling was planted approximately 0.91 m (3 ft) apart with border IWG plants surrounding the nursery (Supplementary Figure S1). The nine half-sibs from each family were planted adjacent to each other. Because of a few dead plants in the nursery and poor genotype data for others, the final number of UMN_C4 parental genets used in this study was 749 out of 771 originally planted.

Seed sampling for this study was done by selecting fifteen mother plants (i.e., 15 families) during 2019 in the nursery that were positioned away from the plot borders. Upon maturity, 10 seed-bearing inflorescences were harvested per plant, dried at 36°C for 72 h, and threshed using a Wintersteiger LD 350 (Wintersteiger Inc., Salt Lake City, United States). From the threshed grain, half-sib progeny were selected for genotyping at random without any preference for seed size, color, or its hulled state. The number of sampled half-sibs per mother plant ranged from 44 to 72 with an average of 56 and a total of 846.

### Genotyping

Seeds sampled from the 15 mother genets (families) were germinated and maintained in a greenhouse during September–December in 2019. From each seedling 10–12 cm, leaf tissue was collected and dried on silica for 5 d. Leaf tissue from UMN_C4 genets were collected in July 2018. DNA was extracted using the BioSprint 96 DNA Plant Kit (QIAGEN, Valencia, CA) and sequenced using the genotyping by sequencing method (Poland et al., 2012) on Illumina Novaseq 6,000 at the University of Minnesota Genomics Center. Obtained reads were quality filtered (Q ≥ 30) and aligned to the *Thinopyrum intermedium* v2.1 reference genome (Thinopyrum intermedium Genome Sequencing Consortium Thinopyrum intermedium v2.1 DOE-JGI, n.d.) using *bwa* 0.7.5a (Li and Durbin, 2009). SAMtools 1.6 and BCFtools 1.6 (Li et al., 2009; Li, 2011) were used with default parameters for detection of single nucleotide polymorphism (SNP) markers among the progeny and parents. SNP markers were filtered for a minimum minor allele frequency of 3% to allow for inclusion of rare alleles. Loci with missing proportion of >10% were discarded. Filtration steps resulted in 21,256 SNP markers that were imputed using the LD-kNNi method (Money et al., 2015) in Tassel 5.2.70 (Bradbury et al., 2007). Imputation accuracy was estimated within Tassel and found to be 94.2%.

### Genetic Diversity

The amount of genetic diversity among the parents and sampled progeny were characterized by number of alleles per locus (A), observed and expected heterozygosity (H_o_, H_e_, respectively), polymorphism information content (PIC), and number of loci assumed to be following Hardy–Weinberg equilibrium; all of these parameters were estimated in Cervus 3.0.7 (Kalinowski et al., 2007). We note that this measure of diversity considers only heterozygosity at polymorphic SNPs analyzed in this study (and excludes invariant sites) and is therefore appropriate for comparing variation within this study but is not an absolute measure of diversity.

We also estimated pairwise fixation index (F_ST_) using Weir and Cockerham’s method (Weir and Cockerham, 1984) using the R package *snpStats* 1.40.0. Because of a large number (~85 million) of data points for each of the families, the estimates of genetic diversity have been averaged over the genome per mother genet (family). Population structure was analyzed from a subset of 2,500 SNPs (detail below) using two methods: (1) principal component (PC) analysis with the function *prcomp* in R 4.0.2 (R Core Team, 2021), and (2) using STRUCTURE V2.3.4 (Pritchard et al., 2000) with number of subgroups (k) = 15, one for each family.

### Parentage Analysis

We used Cervus 3.0.7 to carry out paternity analysis on 846 half-sib progeny collected from 15 mother plants. Initially, all 21,256 genomic markers were used which led to the program halting with a “floating-point overflow error.” The number of markers was then reduced to a randomly selected set of 2,500 in a similar approach to a recently published study in IWG (Crain et al., 2020). For paternity analysis, the number of offspring was set to 10,000 and number of potential fathers as 749. Proportion of loci mistyped was set to 10% as this was the maximum amount of missing data allowed during SNP filtering. Proportion of loci typed was left at the default value generated by the program. A minimum of 300 loci were required for the analysis with confidence levels of 80% (relaxed) and 95% (strict) yet fathers were identified based on 95% confidence levels of LOD scores. We did not rely on the delta (Δ) estimate because it represents the difference in LOD scores of the two most probable fathers (Kalinowski et al., 2007; Jolivet et al., 2012). As our UMN_C4 nursery had 9 half-sib plants per family, it is possible that more than one half-sib of the same family could have pollinated the same mother plant. In such a case, data interpretation based on Δ would lead to ambiguous and incorrect paternal assignment.

Categorical paternity assignment methods, such as CERVUS, may seldom over-predict the number of fathers, especially that of genetically distant pollinators (Jolivet et al., 2012). Therefore, the effective paternity number was re-calculated using an unbiased estimator described by Nielsen et al. (2003). We also implemented the Kolmogorov–Smirnov test (K-S test; Massey, 1951) in order to investigate if successful pollination events were a function of distance between the pollen donors and maternal plants. The K-S test was carried out by comparing the frequency distribution of progeny (i.e., indirect measure of paternal fitness) with distance of pollen donors in the nursery; this was accomplished by using the function *ks.test* in R 4.0.2 (R Core Team, 2021).

### Pollen Dispersal and Mating System Parameters

Spatial positions of all potential fathers in the IWG spaced-plant selection nursery were known *a priori*. Effective pollen dispersal was therefore calculated as the distances between a mother genet and the predicted father based on the CERVUS analysis. We also estimated the axial standard deviation of pollen dispersal using the following formula proposed by Llaurens et al. (2008):


(σpollen)2=12M

where M is the average of squared distance between putative father and mother plants.

Estimation of mating system parameters was done using MLTR 3.4 (Ritland, 2002). Specifically, we estimated multi-locus outcrossing rate (t_m_), single-locus outcrossing rate (t_s_), bi-parental inbreeding (t_m_-t_s_), and correlated paternity (r_p_). Parameter estimations were done with the Newton–Raphson procedure (Jennrich and Sampson, 1976) and by marking the known maternal parents. Each run was bootstrapped 1,000 times by resampling from maternal families.

### Diversity Indices and Domestication Traits

In order to measure the potential impact of reproductive success on genetic composition of the progeny, we studied the relationship between pollination distance and several measures of population diversity. These measures of diversity include: (i) a pairwise genetic relationship among the progeny estimated using the function *A.mat* in the R package *rrBLUP* 4.6.1 (Endelman, 2011), (ii) Nei’s *D* using the R package *StAMPP* (Pembleton et al., 2013), (iii) Shannon’s diversity index (*H*; Hill, 1973), and (iv) rarefaction index (Heck et al., 1975); Shannon’s diversity index and rarefaction index were measured using the function *diversity* in the R package *vegan* 2.5–7 (Oksanen et al., 2020). Differences in mean values of diversity indices, wherever applicable, among the pollination distance bins were tested using the least significant difference (LSD) test at *α* = 0.05 with function *LSD.test* in the R package *agricolae* 1.3–3 (De Mendiburu, 2020). All statistical analyses done in R were done in R 4.0.2.

We also investigated if pollination distance had any effect on trait distribution of the progeny. In the IWG breeding program at the University of Minnesota, a large portion (~3,000–3,500 genets) of the half-sib progeny are not evaluated in the field. Rather, an *in silico* marker-based selection approach, commonly known as genomic selection (Meuwissen et al., 2001), is implemented to select the best 70–75 individuals. These individuals are inter-crossed in a greenhouse and progeny are planted in the field as the next cycle in a spaced-plant selection nursery, similar to the one used in this study. The spaced-plant nursery is evaluated for several agronomic and domestication traits, such as days to heading and anthesis, plant height, grain yield, spike and seed characteristics, resistance to grain and spike shatter, percentage of de-hulled grain (threshability hereafter), and disease resistance. As IWG is a novel crop undergoing domestication, rapid improvement of domestication traits is a priority. The relationship between pollen dispersal and progeny trait distribution has never been evaluated in this crop. Thus, we investigated if the genome-predicted trait performances are affected by differences in pollination distance. For the purpose of this study, we focused on six important agronomic and domestication traits: grain yield per spike (an indirect measure of fertility), heading, plant height, shatter resistance, threshability, and seed mass. Grain yield is measured after threshing individual spikes using a Wintersteiger LD 350 (Wintersteiger Inc., Salt Lake City, United States). Heading is recorded after nearly 50% spike emergence per plant. Plant height is measured from the ground to the tip of the tallest spike. Shatter resistance is measured as the percentage of seed, spikelet, or a portion of spike that break off from the spike. Threshability is measured by evaluating the proportion (%) of grain that are de-hulled. Seed mass (expressed as thousand kernel weight) was measured by weighing 100–300 de-hulled seeds. Traits were fitted on a genomic selection model that accounted for genotype by environment effect (Bajgain et al., 2020a) and were used to predict the trait values of the progeny sampled for this study.

## Results

### Genotyping and Population Diversity

Genotyping of the UMN_C4 population and 846 progeny from the selected 15 mother genets resulted in 21,256 high-quality SNP markers that met our filtering criteria of minimum minor allele frequency of 3% and maximum missing proportion of 10%. For this study, we used a randomly selected set of 2,500 genome-wide SNP markers. The mean marker distribution was 119 per chromosome with a minimum of 53 (Chromosome 21) and maximum of 222 (Chromosome 20). Minor allele frequency for these markers ranged 0.04–0.25 with an average of 0.10.

Measures of population diversity estimated from the 2,500 SNP markers are summarized in Supplementary Table S1 and sequencing and quality metrics on the markers are shown in Supplementary Table S2. Because of the large number of markers used, we discuss these metrics with reference to each family and the overall population rather than individuals. The average number of alleles observed in the maternal families ranged from 2 to 3. Observed genome-wide heterozygosity across all 15 families ranged from 0.39–0.40. Polymorphism information content was moderately low with an average of 0.29 per family and ranged from 0.28–0.30. As maternal parents were known in the nursery, we estimated the non-exclusion probability (N_E_) for the second parent (i.e., paternal genets), which was 0.93 for each of the maternal families. The proportion of markers that deviated from Hardy–Weinberg equilibrium were low with 102 (4.1%) markers on average per family and ranged from 92 to 124 markers (3.7–5.0%).

**Figure 1:**
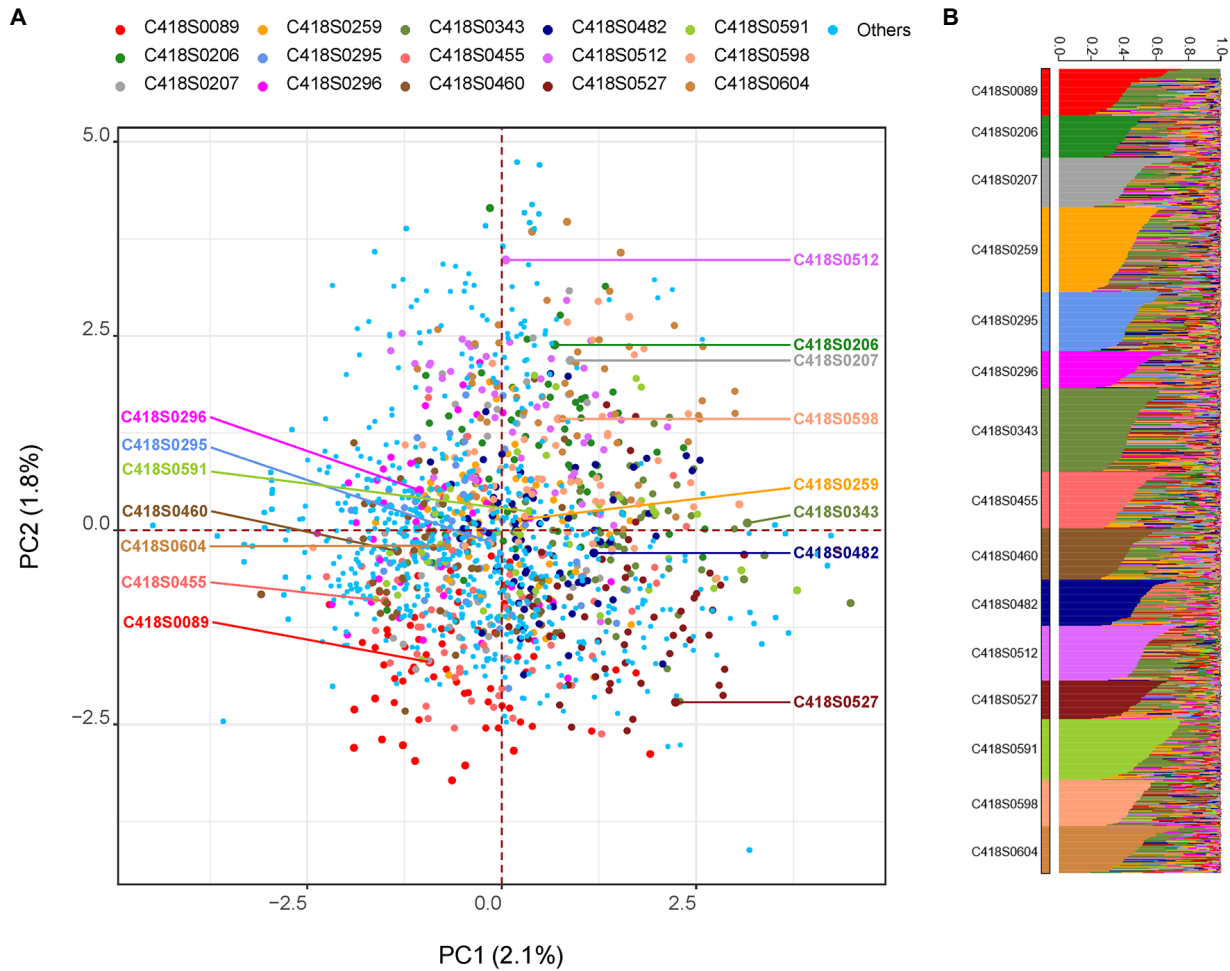
Genetic relationship among the sampled progeny population visualized using: **(A)** principal component (PC) analysis and **(B)** STRUCTURE analysis. In Panel **(A)**, mother genets are labeled with text and progeny sampled from the respective mother genet are highlighted with the same color. All remaining genets in the nursery (including putative paternal genets) are labeled “Others.” In Panel **(B)**, population structure was inferred using the program STRUCTURE by setting *K* = 15. The 15 clusters (families) are displayed in ascending order and individuals within each cluster are sorted by *Q* value.

Genetic relationships among the sampled progeny and their mother plants as well as the whole nursery are shown in Figure 1. Weir and Cockerham fixation index (F_ST_) values were relatively high with an average of 0.24 and ranged from 0.19–0.28. While all families grouped together, principal component (PC) analysis indicated weak population stratification with the first two axes explaining only 3.9% of the overall genetic diversity (Figure 1A). A STRUCTURE analysis with 15 clusters (*k* = 15, one for each family) grouped progeny derived from a mother plant within same clusters (Figure 1B). On average, 84% (median = 87%) of progeny derived from a mother plant were placed within the same cluster.

### Mating System and Paternity Analysis

High outcrossing rates were observed in the 15 IWG families with high multi-locus outcrossing rate (t_m_) of 1.041 and single-locus outcrossing rate (t_s_) of 0.997 (Table 1). The rate of inbreeding was extremely low at 0.003. These results confirm the conventional knowledge of IWG being a near-obligate outcrossing species. Correlated paternity (r_p_), a measure of diversity of the pollen cloud received by the maternal plant, was estimated to be 0.180 and was significantly different from zero (*p* = 2.42E-07).

**Table 1:** Summary of mating pattern and pollen diversity and parentage analysis. Values in parenthesis are standard deviations.

Metric	Value
Multi-locus outcrossing rate (t_m_)	1.041 (0.02)
Single-locus outcrossing rate (t_s_)	0.997 (0.05)
Bi-parental inbreeding (t_m_-t_s_)	0.003 (0.04)
Correlated paternity (r_p_)	0.180 (0.16)
Mean progeny per mother plant with paternity assigned	51
Mean progeny per mother plant with paternity unassigned	5
Mean progeny number for a parent pair	1.40 (1.31)
Maximum progeny number for a parent pair	18
Total number of fathers	549
Number of unique fathers	374
Mean fathers per mother plant	37
Mean/range of pollination distance (m)	6.89/0.91–27.28

**Figure 2:**
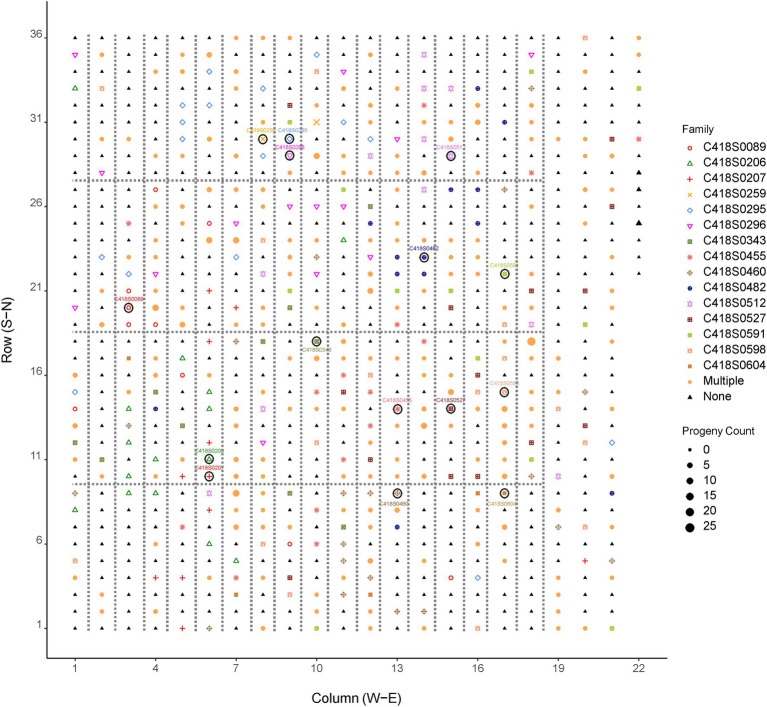
Distribution of pollen donors in the cycle 4 IWG breeding population (UMN_C4) at the University of Minnesota. Symbols enclosed within a circle are mother plants. The position of fathers in the breeding nursery are indicated by shapes and colors of the family/mother plant. Larger shapes indicate more progeny fathered by a given genotype. Fathers of more than one progeny are represented by the shape and color labeled as “Multiple.” “None” indicates plants that were not identified as fathers of the mother plants and their progeny sampled for this study. Plants enclosed within dotted lines are half-sibs; plants not enclosed within the dotted lines are F_1_ genets obtained from bi-parental crosses.

Results of paternity assignment from CERVUS of the 846 progeny (mean of 56 per mother plant) are also summarized in Table 1 and Figure 2. We identified a total of 562 paternal genets that pollinated the 15 mother genets, of which 374 were unique fathers. The number of unique parent pairs was 549 with each pair responsible for 1.4 progeny on average. The number of father genets that pollinated each mother plant ranged from 19 to 47 with a mean value of 36.6. The Nielsen method also predicted the average number of effective fathers per mother to be 36.6. Each father genet was responsible for 2.1 progeny on average with a maximum of 19 progeny fathered by the same paternal genet (C418S0631). Most (87%) paternal genets were responsible for 1–3 progeny. Of the 15 mother genets, 13 (all except C418S0089 and C418S0207) were also identified as the fathers of progeny sampled from remaining mother plants. Of 846 progeny, 77 (9%) had no paternity assigned. Although we observed almost no self-fertilization, we find many cases of bi-parental inbreeding, as half-sibs of mother plants were fairly successful. On average, across all mother plants, 5.5% of father plants were half-sibs planted adjacent to the mother genet (Supplementary Table S3).

### Pollen Dispersal

**Figure 3:**
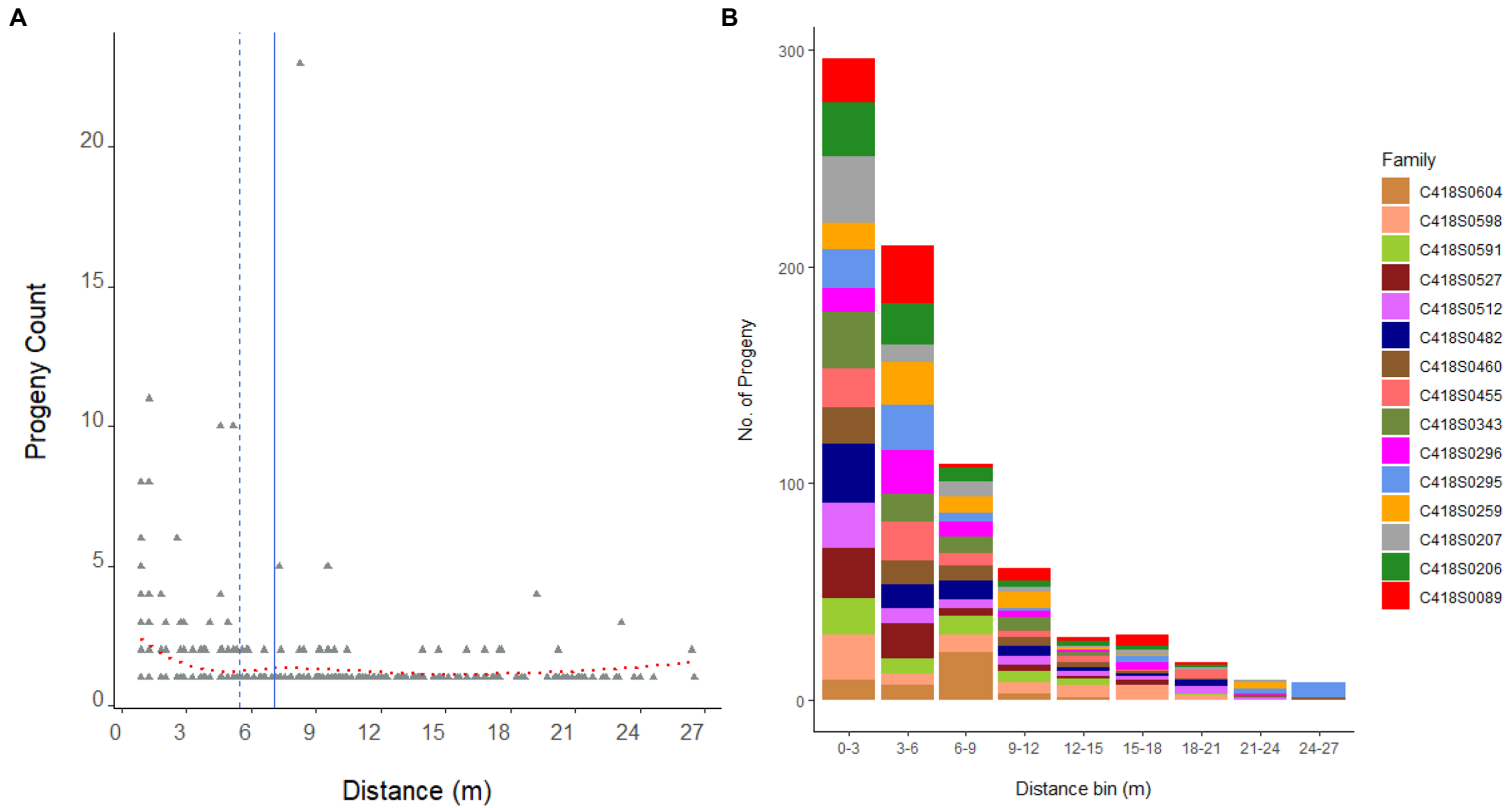
Distribution of 846 progeny from 15 mother genets over pollination distance **(A)**. Dashed blue line is the median distance and solid blue line is the mean distance. Dotted red line is the locally weighted polynomial regression (LOWESS) curve. Panel **(B)** displays the distribution of progeny per mother plant, that is, family sizes sampled over pollination distance bins.

A frequency distribution of progeny over pollination distance in the UMN_C4 IWG spaced-plant selection nursery is shown in Figure 3 and summary data is provided in Table 1. Pollen donors were distributed randomly in the nursery with no particular direction favored for pollination (Figure 2; Supplementary Figure S2). Most fathers (*n* = 60, 11% of all fathers) were located in the West–Northwest direction whereas the North–Northeast direction had the least fathers (*n* = 19, 3.5% of all fathers). The direction of paternal plants and their distances from the maternal plants were negatively correlated and non-significantly different from zero: Pearson’s correlation coefficient *r* = −0.10, *p* = 0.16. During the week of anthesis (07/01/19–07/07/19), air movement in the East–Southwest direction was minimal (2.3 kph or 1.4 mph) yet frequent (Supplementary Table S4). Air movement in the South–Southwest direction, the area of the field with the least number of father plants that successfully pollinated the 15 mother plants, during this week was the third-highest (5.0 kph or 3.1 mph).

The average pollination distance of a mother genet, that is, the average distance between a parent pair, was 6.9 m (median distance = 5.6 m); and the furthest father (C418S0072) in the nursery was located 27.28 m away from the mother genet C418S0295 (Figure 3). Mating success was the highest among close-range genets and reduced with increasing distance: paternal assignment of approximately 52% of the progeny population placed their father genets within 5 m of the mother plants (80% within 10 m). A frequency distribution of father genets that pollinated each mother plant and their distances from the mother plant are shown in Supplementary Figure S3.

**Figure 4:**
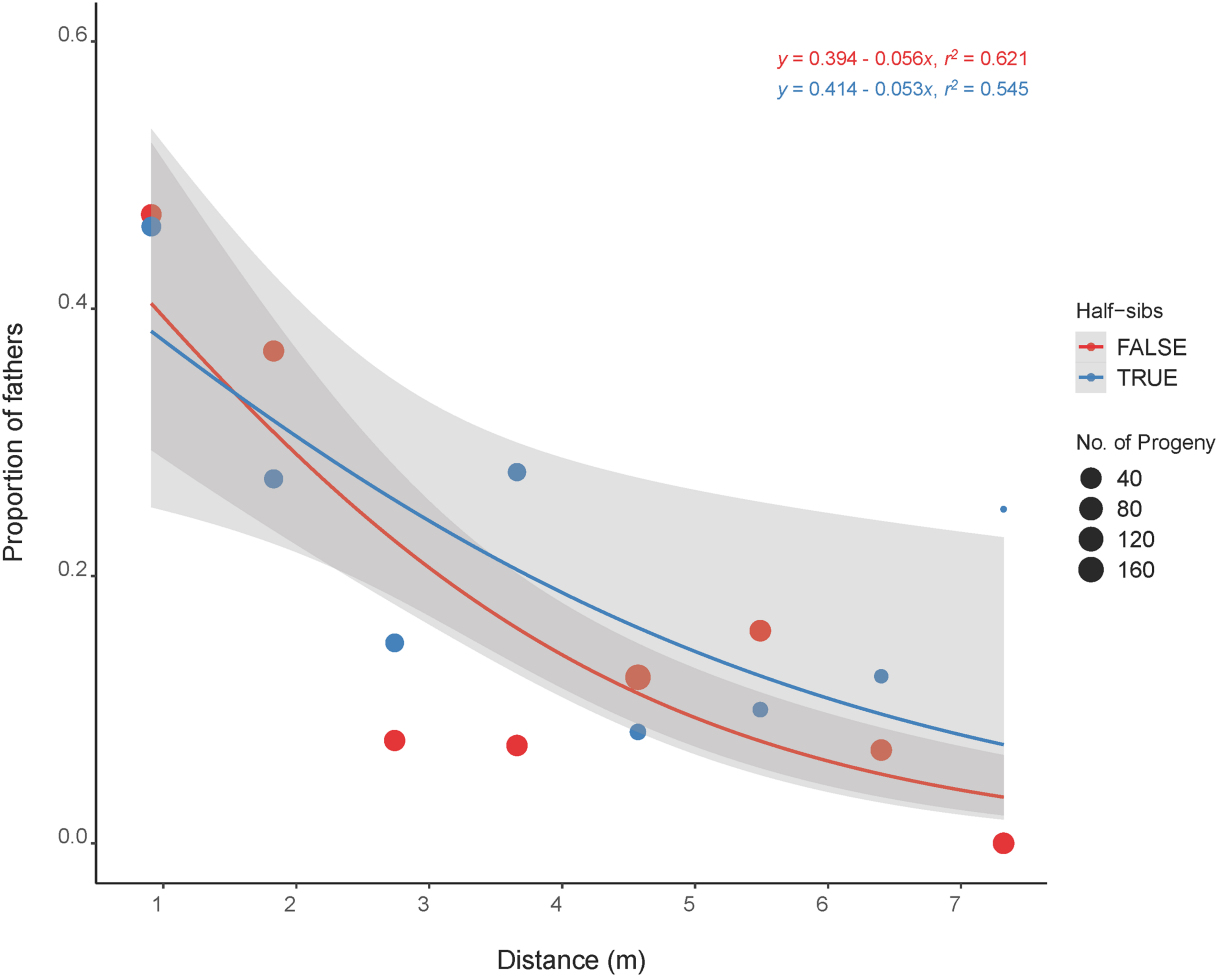
Distance of mother plants that were pollinated by their half-sibs (blue line) and non-half-sibs (red line). Dot sizes are proportional to the total number of progeny fathered by the father plants located at specific distance from the mother plants. Shaded regions are standard error estimates of the distribution at 95% confidence interval.

The majority of the half-sib plants (60%) that fathered an offspring were located within 1.8 m of the mother plant with 40% located next (0.91 m) to the mother plant (Figure 4). The furthest distance of a half-sib father (C418S0199) from the mother plant (C418S0207) was 7.3 m. A Kolmogorov–Smirnov test (*D* = 0.67112, *p* < 2.2E-16) between pollination distance and progeny count suggested that the effect of pollen distances on progeny frequency was non-random.

We also estimated the axial standard deviation distance for pollen dispersal using the method of Llaurens et al. (2008): (σ_pollen_)^2^ = ½ M, where M is the average of squared distance between putative father and mother plants. In our study, this distance (σ_pollen_) was observed to be 4.96 m. This distance was similar to our apparent mean pollination distance of 6.9 m (median = 5.6 m). Neither strong nor significant correlations were observed between trait attributes of the father genets and their success in pollination (number of progeny per father): Pearson’s correlation coefficient (*r*) of 0.08 for plant height, 0.08 for days to heading, and 0.06 for spring growth vigor. Likewise, poor correlations were observed between paternal traits and pollination distance: −0.10 for plant height, 0.08 for days to heading, −0.11 for spring growth vigor.

### Consequences of Pollen Dispersal on Progeny Diversity and Traits

Genetic distance among the progeny were not significantly different among the pollination distance bins (Table 2). Nei’s *D*, a measure of diversity per locus, was significantly (*p* < 0.05) different between bins 6–9 m (0.0131) and 12–15 m (0.0138) but not among the remaining bins. Shannon’s diversity index (*H*), which measures the overall community diversity, was similar among the bins except for the last bin that had a value close to zero. Rarefaction index, a measure of community richness, was also similar among the bins except the last bin where the value was two. A similar trend was observed for diversity estimates of the progeny fathered by the half-sib fathers as no significant differences were observed among the distance bins (Supplementary Table S5). These estimates were also similar to those of the progeny fathered by the entire nursery, except for the rarefaction index in the distance bin 6–9 m where progeny fathered by half-sib plants had an estimate of 2 compared to 7.16 for the progeny fathered by the at-large population in the same distance bin (Supplementary Table S5).

**Table 2:** Diversity indices in the progeny population as a function of pollination distance bins.

Pollination distance bin	Genetic distance	Nei’s *D*	Shannon diversity index	Rarefaction
*m*			*H*	
0–3	0.36a	0.0132ab	2.66	7.40
3–6	0.36a	0.0133ab	2.60	7.23
6–9	0.36a	0.01310b	2.56	7.16
9–12	0.36a	0.0132ab	2.62	7.57
12–15	0.36a	0.01380a	2.48	7.61
15–18	0.36a	0.0135ab	2.27	6.88
18–21	0.36a	0.0133ab	2.04	6.67
21–24	0.36a	0.0134ab	1.68	6.00
24–27	0.37a	0.0133ab	0.38	2.00

Values with different letters, wherever applicable, are significantly different at *α* = 0.05 level according to the LSD test among the bins.

**Figure 5:**
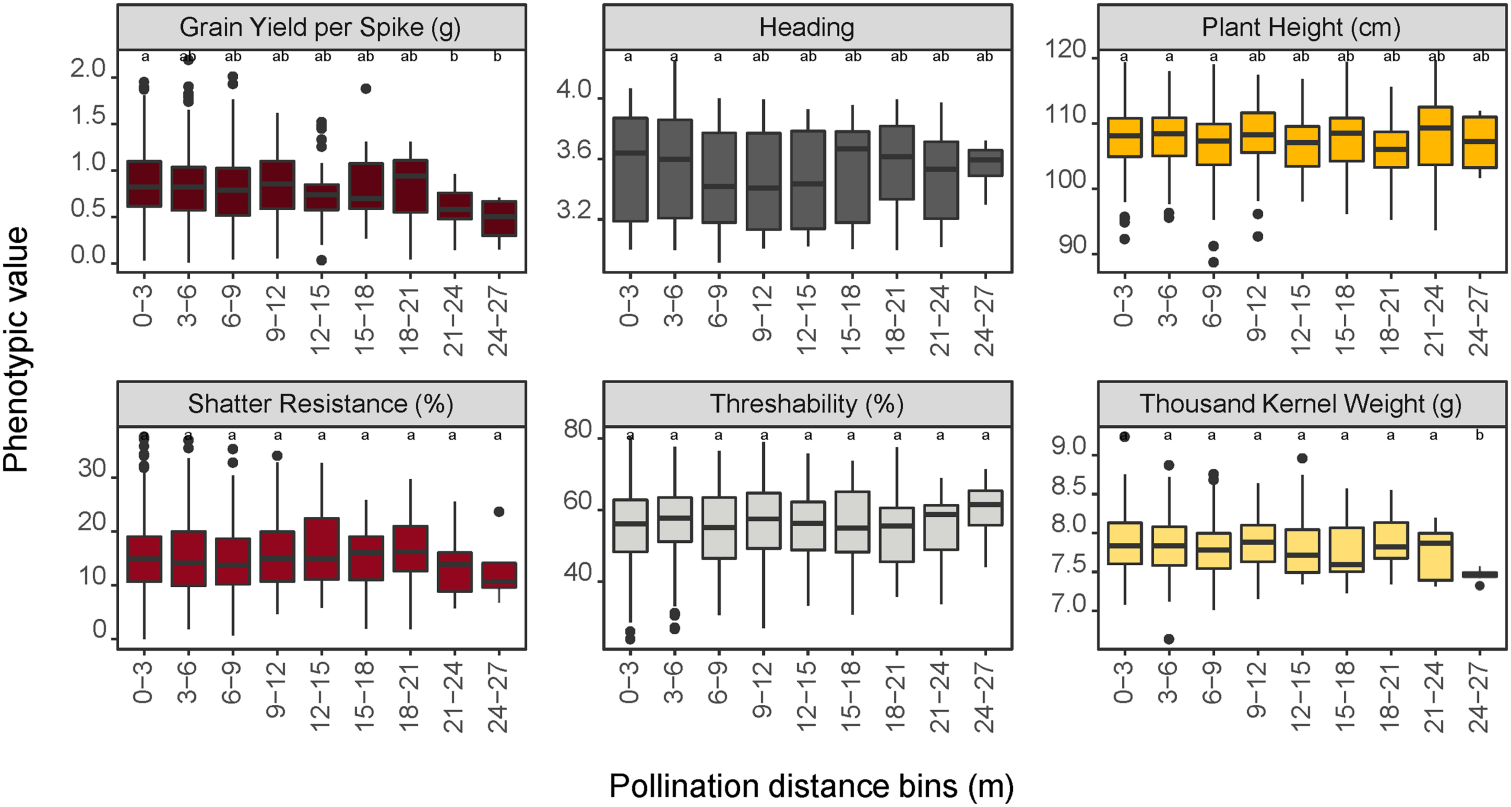
Impact of pollination distance bins on trait performance of the progeny population predicted by a genomic prediction model. Values with different letters are significantly different at *α* = 0.05 level according to the LSD test among the bins. For shatter resistance, a lower trait value is preferred (i.e., less prone to shatter in the field) whereas a higher value is preferred for threshability (more % of de-hulled grain) and thousand kernel weight (larger seeds).

The relationship between pollination distance and trait data of the progeny was investigated by using genome-predicted trait values from a genomic selection model. Predicted trait values from a genomic selection model were used because the progeny are not evaluated in the field prior to their selection for advancement in our breeding scheme. For grain yield per spike, progeny in the furthest distance bins (21–24 and 24–27 m) were significantly different (*p* < 0.05) from progeny in the 0–3 m bin (Figure 5). For the traits heading, height, shatter resistance, and threshability, no significant differences were observed in trait means of progeny sampled from different distance bins. For seed mass (thousand kernel weight), only the progeny in the last bin (24–27 m) had significantly different trait mean relative to other bins. This difference could also be attributed to the small sample size (*n* = 8) in this bin.

## Discussion

Our primary goal in this study was to study pollen dispersal in an IWG breeding nursery and how it affects genetic diversity of and trait distribution in the progeny. We used genome-wide SNP markers to estimate population diversity and study the patterns of pollen dispersal and establish paternity in our advanced IWG breeding population. We observed an average heterozygosity of 0.39 in our IWG breeding population. This value is larger than the observed heterozygosity in other perennial grasses, such as perennial ryegrass (*Lolium perenne* L.) with a range of 0.17–0.32 (Lin et al., 2016), false brome (*Brachypodium sylvaticum*) with value of 0.19 (Steinwand et al., 2013); and range of 0.04–0.1 in switchgrass (*Panicum virgatum*; Lovell et al., 2021). Likewise, we observed a mean fixation index (F_ST_) of 0.24 in our population which has been shown to range 0.01–0.47 in different perennial ryegrass populations (Wang et al., 2014) and 0.05–0.07 in switchgrass (*Panicum virgatum*; Evans et al., 2018). The IWG breeding population used in this study therefore appears to have reasonably high level of population diversity.

In this study, we use paternity assignment to obtain insight about the crop’s mating system, genetic diversity, and pollen dispersal for the first time while exploring the utility of paternal assignment in IWG under field conditions. Toward this goal, we evaluated 846 progeny from 15 mother plants with 749 putative fathers. The average number of paternal genets that contributed pollen per mother plant was quite high (mean of 37). Most fathers of the progeny were located within a short range from the mother plants, although pollen flow was recorded from longer distances as well. We observed a very high outcrossing rate with inbreeding virtually absent (< 1%). The multi-locus outcrossing rate (t_m_ = 1.041) was greater than the theoretical maximum value of 1, which suggests a near-obligate outcrossing nature of the species, in accordance with the known mating behavior of IWG. Multi-locus outcrossing rate greater than 1, that is, an upward bias of t_m_, could result from a computational drawback where large number of DNA markers increase t_m_ (Ritland, 2002) and have previously been reported in the literature (João Gaspar et al., 2009; Ferreira et al., 2010; Ottewell et al., 2012). IWG is predominantly a wind-pollinated species with pollen traveling up to 125 m and rarely beyond 200 m (Jones and Newell, 1946). We observed the highest frequency of pollination among genets that were within a short distance of one another. The median distance between a pollen donor and a mother plant was 5.6 m and approximately 80% of the progeny were obtained from parent pairs that were located within 10 m of each other. The longest observed pollination distance was 27.3 m despite the plant nursery being nearly 33 m long on the North–South direction with a maximum diagonal distance of 38.6 m. Additional study would be needed to thoroughly explore pollination frequency and mating success at larger distances.

In other perennial forage grasses of the Pooideae subfamily which IWG is a member of, pollen travel has been observed at distances >200 m (Wipff and Fricker, 2001; Wang et al., 2004) and up to 21 km (Watrud et al., 2004). Pollen traps by Jones and Newell (1946) showed that nearly 80% of IWG pollen was found within 25.1 m (five rods) from the plot with IWG plants. It is also known that synchrony of flowering time among neighboring plants can reduce the distance of pollen dispersal because of a high volume of pollen present within a short distance (Augspurger, 1980). Additionally, pollen from genets planted in close proximity to the mother plants were more successful in pollination compared to genets located further away from the mother plants. Taken together, we conclude that pollination in IWG occurs preferentially among neighboring genets. Pollen dispersal studies conducted in outcrossing non-grass species have also established similar preferential pollen flow where most-effective pollination was observed among the neighbors and pollen volume reduces as pollination distance increases (Robledo-Arnuncio and Gil, 2005; Oddou-Muratorio et al., 2011; Jolivet et al., 2012). While pollen dispersal and gene flow in these studies are modeled based on plant nurseries that were established in confined fields or set boundaries, the experimental design and lessons learned could be applicable in naturally occurring plant populations of perennials and annuals alike. Knowledge obtained on mating systems, gene flow, and overall diversity of important crop populations can not only shed light on their domestication history, but potentially also assist in their genetic improvement (Civáň et al., 2013; Smýkal et al., 2018).

We observed low correlations between pollination distance and trait attributes of the parental genets. In other words, no parent was favored for pollination efficiency because it was taller or flowered earlier than other paternal candidates. The density of flowering plants and their spatial distribution can affect outcrossing rates and pollen dispersal (Murawski and Hamrick, 1991; Dick et al., 2008). On a similar note, not all neighboring paternal genets close to one of the 15 mother plants were identified as fathers of the progeny sampled from the closest mother plant. Mating incompatibility could be a reason why we did not observe a higher frequency of pairs among neighboring genets (Crain et al., 2020). Another reason could be from the progeny size we sampled per mother plant. A single IWG plant in spaced-plant selection nursery is capable of producing thousands of seed. We sampled <100 seed per mother plant as this is representative of our breeding design when the progeny are sampled and sequenced for genomic prediction. Since we were unable to genotype all progeny per mother plant, evaluation for mating incompatibility among all possible pairs was not possible. We estimated the average number of pollen donors using CERVUS and used the method of Nielsen et al. (2003) to confirm CERVUS findings; both approaches concluded the average number of fathers per mother plant is the same: that is, 38 based on an average of 56 progeny/seed examined per mother plant.

While pollination success was more pronounced within shorter distances, the distance between maternal and paternal genets did not significantly affect progeny genetic diversity and their trait distribution (Table 2; Figure 5). Progeny distributed in nearly all distance bins had similar diversity indices except for the most distant bin (24–27 m) which had skewed estimations of diversity indices. This distance bin has only 8 progeny from two mothers: C418S0295 and C418S0460. The indices estimations are therefore biased due to a small sample size, as genetic variation is related to population size (Nei et al., 1975; Frankham, 1996). The Shannon diversity index (*H*) indicates more diversity with an increasing value of *H* and was nearly identical for all groups except the most distant bin that had no meaningful diversity. The rarefaction index, which is a method used to compare communities when sample sizes are different, indicated that the offspring sampled from different distance bins are quite similar to each other despite the progeny count favoring the short distance. This was true for progeny fathered by the entire nursery as well as the progeny fathered by half-sib genets adjacent to the mother plants (Supplementary Table S5). Despite the half-sibs being planted next to the mother plants, diversity estimates of the progeny were not significantly different than those observed in the overall progeny population.

The half-sib fathers were in fact more successful in pollinating the mother plants within a short distance (2–7 m) except for the immediate 1–2 plants (Figure 4). We therefore did not see strong evidence for incompatibility in pollination among the half-sibs. This also corroborates the overall mating trend in the whole plant nursery as a whole where most pollination occurred within 10 m or less. Additionally, this is indicative that randomization of the half-sibs during their establishment in the nursery might not be necessary. The self-incompatibility (SI) system in IWG is hypothesized to be gametophytic and controlled by two independent genes, *S* and *Z* (Lundqvist, 1954; Crain et al., 2020). While the effect and extent of SI in IWG half-sibs yet to be studied, successful mating among half-sibs could help accumulate favorable alleles and thus aid in trait improvement. Our findings hence indicate that there are no significant differences in progeny performance when sampled from different distance bins. In other words, the distance of pollinator in a breeding nursery does not appear to be a major contributor in maintaining diversity in IWG. Despite pollination mostly occurring within short distances, formation of parent pairs appear to be random as nearly half of the breeding nursery were fathers of the progeny sampled in this study. While the progeny grouped with their mother plants, no strong population stratification was observed (Figure 1) and is consistent with our previous findings of a lack of strong population substructure in our IWG breeding population (Bajgain et al., 2019). In collective, these findings suggest that the danger of loss in genetic diversity and population fragmentation with subsequent population advancement in our IWG breeding nursery is minimal.

Likewise, we were interested in learning if the distance between mother and father genets, that is, pollination distances, has any impact on progeny performance with regard to important domestication traits, such as shatter resistance, threshability, and grain mass. The IWG breeding programs apply strong selection pressure in the breeding population by selecting the few best performing mother plants (out of several hundred) for the aforementioned traits, which can potentially reduce genetic diversity of the progeny. As the locations of these selected mother plants can be randomly distributed in the field, we were curious to know if distances among mother and father plants had any effect on progeny diversity and trait performance. The effect of pollination distances on genetic diversity and trait performance in the progeny population (often F_1_) of other perennial species has been found to vary depending upon the trait type and diversity of the parent population (Becker et al., 2006; Raabová et al., 2009). In this IWG population, there was no significant difference in trait mean values among the progeny grouped by distance bins for the traits shatter resistance, threshability, and grain size (Figure 5). For these important domestication traits, our results thus demonstrate that both diversity and trait distribution of progeny are not affected by distance of fathers from their mother plants. This finding also validates our current method of progeny selection using predicted *per se* performance, that is, best values for traits of interest as predicted by genomic selection models, can be continued without a substantial reduction in genetic diversity of future populations.

In summary, we characterized pollen dispersal in IWG, a cross-pollinated perennial grass crop. Our results indicate that the majority of the pollination events in IWG occur within 10 m although pollen can travel longer distances to a mother plant. Morphological attributes of father plants were poor determinants of pollination success. A major takeaway from evaluation of the progeny population was that the pollination distance had no effect on their genetic diversity. Similarly, distance between maternal and paternal genets had no significant effect on progeny trait values predicted using genomic selection models. IWG breeding programs generally evaluate genets in a spaced-plant selection nursery and rely on genomic selection-based breeding approaches to expedite domestication. Neither genetic diversity nor trait distribution of the progeny was affected by pollination distances. Thus, a progeny population selected from a spaced-plant selection nursery based on genomic-predicted trait values appears less likely to suffer from loss of genetic diversity. Our findings could have implications in investigating and understanding gene flow and population dynamics in open-pollinated breeding programs, especially those targeting domestication of novel and/or orphan crops. Likewise, a similar approach could be implemented to study native agro-ecosystems, for example, prairies and grasslands and wild species in their natural habitat.

## Data Availability Statement

The datasets presented in this study can be found in online repositories. The names of the repository/repositories and accession number(s) can be found at: https://www.ncbi.nlm.nih.gov/, PRJNA722274 https://www.ncbi.nlm.nih.gov/, PRJNA722280 https://www.ncbi.nlm.nih.gov/, PRJNA722532 https://doi.org/10.5061/dryad.73n5tb2zb.

## Author Contributions

PB conceptualized the study, collected or generated the data, performed the data analyses, and wrote the manuscript. YB contributed to the methodology and statistical analyses, helped refine result interpretation, and assisted in writing the manuscript. JA and PB acquired the funding, provided feedback on the study, and assisted in writing the manuscript. All authors contributed to the article and approved the submitted version.

## Funding

Funding for this study was provided by the Forever Green Initiative at the University of Minnesota through the Minnesota Department of Agriculture, General Mills Inc., and PepsiCo. The funders were not involved in the study design, collection, analysis, interpretation of data, the writing of this article or the decision to submit it for publication.

## Conflict of Interest

The authors declare that the research was conducted in the absence of any commercial or financial relationships that could be construed as a potential conflict of interest.

## Publisher’s Note

All claims expressed in this article are solely those of the authors and do not necessarily represent those of their affiliated organizations, or those of the publisher, the editors and the reviewers. Any product that may be evaluated in this article, or claim that may be made by its manufacturer, is not guaranteed or endorsed by the publisher.
