# Supplementary material for: Influence of Pollen Dispersal and Mating Pattern in Domestication of Intermediate Wheatgrass, a Novel Perennial Food Crop
Source: Front Plant Sci. 2022 Apr 28;13:871130. doi: 10.3389/fpls.2022.871130 (PMC9096613; doi:10.3389/fpls.2022.871130)

Supplementary Material

**Supplementary Table S1:** Parameters of population genetic diversity in 15 IWG families of the University of Minnesota’s cycle 4 intermediate wheatgrass breeding population.

| **Family** | **A** | **N** | **Reads** | **HObs** | **HExp** | **PIC** | **N_E_** | **F_ST_** | **HW (%)** |
| --- | --- | --- | --- | --- | --- | --- | --- | --- | --- |
| C418S0089 | 2.270 | 63 (815) | 34.553 | 0.388 | 0.351 | 0.287 | 0.932 | 0.227 | 98 (3.91%) |
| C418S0206 | 2.272 | 65 (817) | 35.112 | 0.388 | 0.352 | 0.287 | 0.931 | 0.271 | 94 (3.75%) |
| C418S0207 | 2.271 | 53 (805) | 30.425 | 0.387 | 0.349 | 0.284 | 0.932 | 0.249 | 92 (3.67%) |
| C418S0259 | 3.067 | 61 (813) | 12.103 | 0.391 | 0.358 | 0.295 | 0.929 | 0.192 | 109 (4.35%) |
| C418S0295 | 3.067 | 58 (810) | 10.330 | 0.391 | 0.356 | 0.292 | 0.930 | 0.199 | 106 (4.23%) |
| C418S0296 | 3.067 | 50 (802) | 12.614 | 0.390 | 0.352 | 0.288 | 0.931 | 0.207 | 106 (4.23%) |
| C418S0343 | 2.283 | 59 (811) | 18.847 | 0.393 | 0.351 | 0.287 | 0.931 | 0.275 | 94 (3.75%) |
| C418S0455 | 2.272 | 65 (817) | 12.493 | 0.396 | 0.354 | 0.288 | 0.931 | 0.276 | 99 (3.95%) |
| C418S0460 | 2.271 | 53 (805) | 17.572 | 0.392 | 0.350 | 0.285 | 0.932 | 0.258 | 93 (3.71%) |
| C418S0482 | 3.067 | 72 (824) | 16.932 | 0.393 | 0.361 | 0.297 | 0.928 | 0.239 | 124 (4.95%) |
| C418S0512 | 2.275 | 46 (798) | 17.472 | 0.390 | 0.347 | 0.283 | 0.932 | 0.255 | 100 (3.99%) |
| C418S0527 | 3.067 | 54 (806) | 12.741 | 0.392 | 0.352 | 0.288 | 0.931 | 0.228 | 99 (3.95%) |
| C418S0591 | 2.969 | 44 (796) | 15.947 | 0.389 | 0.348 | 0.284 | 0.932 | 0.249 | 96 (3.83%) |
| C418S0598 | 3.068 | 56 (808) | 12.260 | 0.392 | 0.354 | 0.290 | 0.930 | 0.222 | 109 (4.35%) |
| C418S0604 | 3.068 | 47 (799) | 12.779 | 0.389 | 0.351 | 0.287 | 0.931 | 0.197 | 109 (4.35%) |
| Mean | 2.690 | 56 (808) | 18.451 | 0.391 | 0.352 | 0.288 | 0.931 | 0.236 | 102 (4.06%) |

**Family**: Family name, same as maternal genet names; **A**: Average number of alleles observed across all loci; **N**: Number of progeny analyzed per family with the total population size in parentheses; **Reads**: family-average of mean sequencing read depth per individual; **HObs**: Observed heterozygosity; **HExp**: Expected heterozygosity; **PIC**: Polymorphic information content; **N_E_**: Average non-exclusion probability for one candidate parent with the assumption that the genotype of the second parent of the opposite sex is known; **F_ST_**: mean Weir and Cockerham fixation index values; **HW**: Number of markers out of 2500 with significant deviation from Hardy-Weinberg equilibrium.

**Supplementary Table S2**: Sequencing and quality metrics on the 2500 SNP markers used in the study.

| SNP | Chr | Pos | Mean depth | Var depth | Sum depth | Qual |
| --- | --- | --- | --- | --- | --- | --- |
| Chr01_8342873 | Chr01 | 8342873 | 27.3847 | 1367.11 | 44281 | 999 |
| Chr01_9585390 | Chr01 | 9585390 | 7.81942 | 81.0404 | 12644 | 999 |
| Chr01_28608466 | Chr01 | 28608466 | 11.9926 | 214.618 | 19392 | 999 |
| Chr01_33413524 | Chr01 | 33413524 | 16.1855 | 368.102 | 26172 | 999 |
| Chr01_36661501 | Chr01 | 36661501 | 3.51515 | 12.3824 | 5684 | 999 |
| Chr01_36661508 | Chr01 | 36661508 | 3.51886 | 12.4181 | 5690 | 999 |
| Chr01_42168991 | Chr01 | 42168991 | 8.72109 | 81.8572 | 14102 | 999 |
| Chr01_42168996 | Chr01 | 42168996 | 8.78417 | 83.2993 | 14204 | 999 |
| Chr01_42168998 | Chr01 | 42168998 | 8.71861 | 81.8484 | 14098 | 999 |
| Chr01_42168999 | Chr01 | 42168999 | 8.69882 | 81.8764 | 14066 | 999 |
| Chr01_44418844 | Chr01 | 44418844 | 9.64255 | 88.3226 | 15592 | 999 |
| Chr01_47269858 | Chr01 | 47269858 | 20.8714 | 690.424 | 33749 | 999 |
| Chr01_50894015 | Chr01 | 50894015 | 7.11379 | 58.1479 | 11503 | 999 |
| Chr01_50894036 | Chr01 | 50894036 | 7.07174 | 57.3018 | 11435 | 999 |
| Chr01_52076376 | Chr01 | 52076376 | 22.7761 | 824.324 | 36829 | 999 |
| Chr01_65777178 | Chr01 | 65777178 | 20.6654 | 335.606 | 33416 | 999 |
| Chr01_72930980 | Chr01 | 72930980 | 5.26036 | 29.4984 | 8506 | 999 |
| Chr01_78726905 | Chr01 | 78726905 | 9.04762 | 102.72 | 14630 | 999 |
| Chr01_102004022 | Chr01 | 102004022 | 10.4632 | 99.1931 | 16919 | 999 |
| Chr01_103705677 | Chr01 | 103705677 | 15.0625 | 296.42 | 24356 | 999 |
| Chr01_114279039 | Chr01 | 114279039 | 6.90785 | 67.309 | 11170 | 999 |
| Chr01_129060687 | Chr01 | 129060687 | 9.29252 | 86.5375 | 15026 | 999 |
| Chr01_142085536 | Chr01 | 142085536 | 8.76314 | 68.4581 | 14170 | 999 |
| Chr01_142085539 | Chr01 | 142085539 | 8.72171 | 67.5797 | 14103 | 999 |
| Chr01_155821352 | Chr01 | 155821352 | 5.671 | 31.7754 | 9170 | 999 |
| Chr01_164025343 | Chr01 | 164025343 | 9.76129 | 81.6249 | 15784 | 999 |
| Chr01_192149997 | Chr01 | 192149997 | 31.7662 | 1011.32 | 51366 | 999 |
| Chr01_203129917 | Chr01 | 203129917 | 10.4997 | 98.5509 | 16978 | 999 |
| Chr01_213805160 | Chr01 | 213805160 | 27.1515 | 1473.14 | 43904 | 999 |
| Chr01_234216169 | Chr01 | 234216169 | 5.75696 | 32.3574 | 9309 | 999 |
| Chr01_234216188 | Chr01 | 234216188 | 5.73531 | 32.1811 | 9274 | 999 |
| Chr01_234216189 | Chr01 | 234216189 | 5.74768 | 32.2296 | 9294 | 999 |
| Chr01_234216229 | Chr01 | 234216229 | 8.29375 | 57.8895 | 13411 | 999 |
| Chr01_234216234 | Chr01 | 234216234 | 8.28262 | 58.0828 | 13393 | 999 |
| Chr01_241357295 | Chr01 | 241357295 | 18.4409 | 590.338 | 29819 | 999 |
| Chr01_254115587 | Chr01 | 254115587 | 9.96722 | 130.054 | 16117 | 999 |
| Chr01_259060563 | Chr01 | 259060563 | 9.80829 | 100.253 | 15860 | 999 |
| Chr01_302338341 | Chr01 | 302338341 | 23.0736 | 1001.28 | 37310 | 999 |
| Chr01_306721056 | Chr01 | 306721056 | 21.9518 | 638.392 | 35496 | 999 |
| Chr01_307647288 | Chr01 | 307647288 | 25.12 | 875.13 | 40619 | 999 |
| Chr01_307919736 | Chr01 | 307919736 | 9.26531 | 95.6777 | 14982 | 999 |
| Chr01_309027695 | Chr01 | 309027695 | 18.0773 | 499.602 | 29231 | 999 |
| Chr01_311143006 | Chr01 | 311143006 | 9.50959 | 141.416 | 15377 | 999 |
| Chr01_323475216 | Chr01 | 323475216 | 5.67904 | 45.6488 | 9183 | 999 |
| Chr01_323475218 | Chr01 | 323475218 | 5.67718 | 45.7757 | 9180 | 999 |
| Chr01_323475229 | Chr01 | 323475229 | 5.55535 | 43.5924 | 8983 | 999 |
| Chr01_326145883 | Chr01 | 326145883 | 12.9864 | 191.09 | 20999 | 999 |
| Chr01_326145885 | Chr01 | 326145885 | 13.039 | 193.458 | 21084 | 999 |
| Chr01_326145892 | Chr01 | 326145892 | 13.0322 | 193.164 | 21073 | 999 |
| Chr01_332939480 | Chr01 | 332939480 | 6.84106 | 61.2736 | 11062 | 999 |
| Chr01_339101017 | Chr01 | 339101017 | 30.4045 | 1238.17 | 49164 | 999 |
| Chr01_339101988 | Chr01 | 339101988 | 28.2616 | 883.597 | 45699 | 999 |
| Chr01_345519469 | Chr01 | 345519469 | 9.03092 | 110.711 | 14603 | 999 |
| Chr01_345657403 | Chr01 | 345657403 | 10.149 | 117.842 | 16411 | 999 |
| Chr01_346672558 | Chr01 | 346672558 | 8.1577 | 103.581 | 13191 | 999 |
| Chr01_349137307 | Chr01 | 349137307 | 6.80643 | 64.3975 | 11006 | 999 |
| Chr01_350269834 | Chr01 | 350269834 | 4.89858 | 33.4031 | 7921 | 999 |
| Chr01_351135100 | Chr01 | 351135100 | 5.26964 | 40.5721 | 8521 | 999 |
| Chr01_352958831 | Chr01 | 352958831 | 23.739 | 783.031 | 38386 | 999 |
| Chr01_355659641 | Chr01 | 355659641 | 20.3902 | 735.721 | 32971 | 999 |
| Chr01_357505244 | Chr01 | 357505244 | 15.8806 | 353.684 | 25679 | 999 |
| Chr01_359829627 | Chr01 | 359829627 | 12.0427 | 220.269 | 19473 | 999 |
| Chr01_363248966 | Chr01 | 363248966 | 8.64007 | 123.114 | 13971 | 999 |
| Chr01_363248970 | Chr01 | 363248970 | 8.66419 | 123.431 | 14010 | 999 |
| Chr01_363248973 | Chr01 | 363248973 | 8.68893 | 124.132 | 14050 | 999 |
| Chr01_368883660 | Chr01 | 368883660 | 10.3377 | 101.596 | 16716 | 999 |
| Chr01_370181130 | Chr01 | 370181130 | 11.2752 | 128.45 | 18232 | 999 |
| Chr01_372196259 | Chr01 | 372196259 | 10.8343 | 132.547 | 17519 | 999 |
| Chr01_375580606 | Chr01 | 375580606 | 13.7069 | 227.615 | 22164 | 999 |
| Chr01_375580621 | Chr01 | 375580621 | 13.6357 | 224.675 | 22049 | 999 |
| Chr01_375580622 | Chr01 | 375580622 | 13.6147 | 225.197 | 22015 | 999 |
| Chr01_375580655 | Chr01 | 375580655 | 13.4094 | 217.877 | 21683 | 999 |
| Chr01_378149050 | Chr01 | 378149050 | 7.82808 | 51.1957 | 12658 | 999 |
| Chr01_382217964 | Chr01 | 382217964 | 15.5628 | 159.205 | 25165 | 999 |
| Chr01_382217972 | Chr01 | 382217972 | 15.5189 | 158.036 | 25094 | 999 |
| Chr01_382217977 | Chr01 | 382217977 | 15.5912 | 160.586 | 25211 | 999 |
| Chr01_383442434 | Chr01 | 383442434 | 6.33828 | 55.4319 | 10249 | 999 |
| Chr01_387578900 | Chr01 | 387578900 | 5.27829 | 35.2517 | 8535 | 999 |
| Chr01_388028629 | Chr01 | 388028629 | 12.0254 | 244.715 | 19445 | 999 |
| Chr01_388028659 | Chr01 | 388028659 | 12.188 | 249.638 | 19708 | 999 |
| Chr01_389466385 | Chr01 | 389466385 | 16.2733 | 306.566 | 26314 | 999 |
| Chr01_395834707 | Chr01 | 395834707 | 6.70068 | 54.45 | 10835 | 999 |
| Chr01_396372529 | Chr01 | 396372529 | 5.31045 | 39.8305 | 8587 | 999 |
| Chr01_396879734 | Chr01 | 396879734 | 9.33704 | 85.6283 | 15098 | 999 |
| Chr01_400069021 | Chr01 | 400069021 | 6.49722 | 30.4506 | 10506 | 999 |
| Chr01_407682437 | Chr01 | 407682437 | 9.85591 | 113.99 | 15937 | 999 |
| Chr01_409896158 | Chr01 | 409896158 | 18.7372 | 467.517 | 30298 | 999 |
| Chr01_411914316 | Chr01 | 411914316 | 27.603 | 780.443 | 44634 | 999 |
| Chr01_411914350 | Chr01 | 411914350 | 30.5615 | 999.803 | 49418 | 999 |
| Chr01_412964082 | Chr01 | 412964082 | 11.2263 | 181.206 | 18153 | 999 |
| Chr01_416306615 | Chr01 | 416306615 | 5.13976 | 37.6537 | 8311 | 999 |
| Chr01_418571025 | Chr01 | 418571025 | 10.7192 | 183.797 | 17333 | 999 |
| Chr01_418604813 | Chr01 | 418604813 | 14.8435 | 263.346 | 24002 | 999 |
| Chr01_421012825 | Chr01 | 421012825 | 5.17687 | 43.7076 | 8371 | 999 |
| Chr01_421299175 | Chr01 | 421299175 | 6.56339 | 68.2944 | 10613 | 999 |
| Chr01_424603534 | Chr01 | 424603534 | 9.53309 | 103.056 | 15415 | 999 |
| Chr01_434013940 | Chr01 | 434013940 | 30.906 | 1088.84 | 49975 | 999 |
| Chr01_435425643 | Chr01 | 435425643 | 14.334 | 213.204 | 23178 | 999 |
| Chr01_437014667 | Chr01 | 437014667 | 16.3525 | 334.17 | 26442 | 999 |
| Chr01_437242702 | Chr01 | 437242702 | 17.329 | 533.612 | 28021 | 999 |
| Chr01_440164986 | Chr01 | 440164986 | 5.59246 | 37.3357 | 9043 | 999 |
| Chr01_440274667 | Chr01 | 440274667 | 8.08782 | 79.8487 | 13078 | 999 |
| Chr01_441127582 | Chr01 | 441127582 | 4.35993 | 24.2677 | 7050 | 999 |
| Chr01_441400500 | Chr01 | 441400500 | 7.15461 | 71.1196 | 11569 | 999 |
| Chr01_443880348 | Chr01 | 443880348 | 6.76623 | 71.0963 | 10941 | 999 |
| Chr01_443880351 | Chr01 | 443880351 | 6.55906 | 66.1761 | 10606 | 999 |
| Chr01_443880359 | Chr01 | 443880359 | 6.35127 | 64.728 | 10270 | 999 |
| Chr01_444570298 | Chr01 | 444570298 | 10.6024 | 188.335 | 17144 | 999 |
| Chr01_444570301 | Chr01 | 444570301 | 10.4954 | 184.334 | 16971 | 999 |
| Chr01_444570307 | Chr01 | 444570307 | 10.6092 | 188.356 | 17155 | 999 |
| Chr01_447174031 | Chr01 | 447174031 | 26.6345 | 1348.52 | 43068 | 999 |
| Chr01_447174047 | Chr01 | 447174047 | 27.2226 | 1385.03 | 44019 | 999 |
| Chr01_448193872 | Chr01 | 448193872 | 6.93816 | 95.3873 | 11219 | 999 |
| Chr01_448193877 | Chr01 | 448193877 | 6.91156 | 95.2663 | 11176 | 999 |
| Chr01_450846019 | Chr01 | 450846019 | 5.01546 | 36.2826 | 8110 | 999 |
| Chr01_450992799 | Chr01 | 450992799 | 7.0303 | 80.2039 | 11368 | 999 |
| Chr01_451284037 | Chr01 | 451284037 | 9.34941 | 140.567 | 15118 | 999 |
| Chr01_451284063 | Chr01 | 451284063 | 9.31416 | 139.079 | 15061 | 999 |
| Chr01_452857428 | Chr01 | 452857428 | 11.4366 | 106.66 | 18493 | 999 |
| Chr01_455269865 | Chr01 | 455269865 | 8.62152 | 90.452 | 13941 | 999 |
| Chr01_455269866 | Chr01 | 455269866 | 8.57823 | 89.0646 | 13871 | 999 |
| Chr01_457989152 | Chr01 | 457989152 | 5.3927 | 40.6978 | 8720 | 999 |
| Chr01_458870936 | Chr01 | 458870936 | 5.61534 | 25.7282 | 9080 | 999 |
| Chr01_459063910 | Chr01 | 459063910 | 5.79901 | 46.3364 | 9377 | 999 |
| Chr01_459079998 | Chr01 | 459079998 | 6.47248 | 65.5415 | 10466 | 999 |
| Chr01_459080016 | Chr01 | 459080016 | 7.00247 | 75.3057 | 11323 | 999 |
| Chr01_460627855 | Chr01 | 460627855 | 8.18862 | 106.115 | 13241 | 999 |
| Chr01_465104506 | Chr01 | 465104506 | 14.4601 | 308.672 | 23382 | 999 |
| Chr01_465416708 | Chr01 | 465416708 | 6.16574 | 46.2101 | 9970 | 999 |
| Chr01_465416714 | Chr01 | 465416714 | 6.15337 | 46.2883 | 9950 | 999 |
| Chr01_465417529 | Chr01 | 465417529 | 13.4001 | 300.454 | 21668 | 999 |
| Chr01_465417541 | Chr01 | 465417541 | 13.3766 | 298.392 | 21630 | 999 |
| Chr01_468065895 | Chr01 | 468065895 | 33.6642 | 1518 | 54435 | 999 |
| Chr01_469600272 | Chr01 | 469600272 | 7.29808 | 93.9507 | 11801 | 999 |
| Chr01_471366235 | Chr01 | 471366235 | 4.95609 | 28.313 | 8014 | 999 |
| Chr01_473632919 | Chr01 | 473632919 | 12.6691 | 201.377 | 20486 | 999 |
| Chr01_473632932 | Chr01 | 473632932 | 12.6475 | 200.472 | 20451 | 999 |
| Chr01_474486768 | Chr01 | 474486768 | 5.23438 | 28.7018 | 8464 | 999 |
| Chr01_474758124 | Chr01 | 474758124 | 8.53803 | 78.2722 | 13806 | 999 |
| Chr01_475088674 | Chr01 | 475088674 | 6.84292 | 51.9654 | 11065 | 999 |
| Chr01_480628857 | Chr01 | 480628857 | 8.84168 | 115.523 | 14297 | 999 |
| Chr01_484420173 | Chr01 | 484420173 | 11.0965 | 137.408 | 17943 | 999 |
| Chr01_484420194 | Chr01 | 484420194 | 11.1911 | 140 | 18096 | 999 |
| Chr01_484648614 | Chr01 | 484648614 | 7.51886 | 65.8463 | 12158 | 999 |
| Chr01_491251729 | Chr01 | 491251729 | 20.8565 | 627.164 | 33725 | 999 |
| Chr01_500814703 | Chr01 | 500814703 | 5.87013 | 48.9039 | 9492 | 999 |
| Chr01_500814714 | Chr01 | 500814714 | 5.88002 | 48.8173 | 9508 | 999 |
| Chr01_500814727 | Chr01 | 500814727 | 5.89549 | 49.0021 | 9533 | 999 |
| Chr01_501841380 | Chr01 | 501841380 | 34.1899 | 1019.87 | 55285 | 999 |
| Chr01_501957185 | Chr01 | 501957185 | 6.10575 | 48.1392 | 9873 | 999 |
| Chr01_503166600 | Chr01 | 503166600 | 14.2127 | 145.632 | 22982 | 999 |
| Chr01_505687950 | Chr01 | 505687950 | 16.9734 | 515.073 | 27446 | 999 |
| Chr02_300679 | Chr02 | 300679 | 13.8015 | 303.856 | 22317 | 999 |
| Chr02_627076 | Chr02 | 627076 | 20.572 | 604.66 | 33265 | 999 |
| Chr02_9740422 | Chr02 | 9740422 | 18.9802 | 545.642 | 30691 | 999 |
| Chr02_12563344 | Chr02 | 12563344 | 7.30674 | 60.2128 | 11815 | 999 |
| Chr02_21225283 | Chr02 | 21225283 | 7.77737 | 73.2511 | 12576 | 999 |
| Chr02_21225321 | Chr02 | 21225321 | 7.47062 | 69.5513 | 12080 | 999 |
| Chr02_47084445 | Chr02 | 47084445 | 11.1045 | 188.346 | 17956 | 999 |
| Chr02_50238642 | Chr02 | 50238642 | 7.80829 | 78.061 | 12626 | 999 |
| Chr02_54233778 | Chr02 | 54233778 | 10.3661 | 85.0391 | 16762 | 999 |
| Chr02_54233784 | Chr02 | 54233784 | 10.4366 | 87.0481 | 16876 | 999 |
| Chr02_54957261 | Chr02 | 54957261 | 10.0303 | 194.154 | 16219 | 999 |
| Chr02_54957262 | Chr02 | 54957262 | 9.99443 | 191.368 | 16161 | 999 |
| Chr02_54957308 | Chr02 | 54957308 | 10.1509 | 197.355 | 16414 | 999 |
| Chr02_64586956 | Chr02 | 64586956 | 14.8275 | 479.652 | 23976 | 999 |
| Chr02_73145739 | Chr02 | 73145739 | 6.53432 | 57.6042 | 10566 | 999 |
| Chr02_73145740 | Chr02 | 73145740 | 6.48237 | 56.6187 | 10482 | 999 |
| Chr02_76093228 | Chr02 | 76093228 | 14.2795 | 310.223 | 23090 | 999 |
| Chr02_76093229 | Chr02 | 76093229 | 14.2288 | 308.013 | 23008 | 999 |
| Chr02_76093236 | Chr02 | 76093236 | 14.2727 | 309.326 | 23079 | 999 |
| Chr02_76093238 | Chr02 | 76093238 | 14.2474 | 308.076 | 23038 | 999 |
| Chr02_80260956 | Chr02 | 80260956 | 8.34199 | 98.9096 | 13489 | 999 |
| Chr02_80260990 | Chr02 | 80260990 | 8.26592 | 97.3327 | 13366 | 999 |
| Chr02_84081854 | Chr02 | 84081854 | 9.20965 | 102.907 | 14892 | 999 |
| Chr02_84978006 | Chr02 | 84978006 | 11.2876 | 143.68 | 18252 | 999 |
| Chr02_96276105 | Chr02 | 96276105 | 10.0359 | 177.457 | 16228 | 999 |
| Chr02_97502824 | Chr02 | 97502824 | 31.9116 | 1913.54 | 51601 | 999 |
| Chr02_101881035 | Chr02 | 101881035 | 13.9802 | 130.151 | 22606 | 999 |
| Chr02_106672569 | Chr02 | 106672569 | 26.6017 | 766.312 | 43015 | 999 |
| Chr02_106672877 | Chr02 | 106672877 | 6.47434 | 49.0849 | 10469 | 999 |
| Chr02_106672900 | Chr02 | 106672900 | 7.1107 | 57.0082 | 11498 | 999 |
| Chr02_106672951 | Chr02 | 106672951 | 7.859 | 66.1893 | 12708 | 999 |
| Chr02_109532915 | Chr02 | 109532915 | 5.97155 | 46.5376 | 9656 | 999 |
| Chr02_109532934 | Chr02 | 109532934 | 6.00804 | 47.0414 | 9715 | 999 |
| Chr02_110093685 | Chr02 | 110093685 | 4.28695 | 23.8174 | 6932 | 999 |
| Chr02_110093686 | Chr02 | 110093686 | 4.25541 | 23.2212 | 6881 | 999 |
| Chr02_110093687 | Chr02 | 110093687 | 4.24119 | 23.0086 | 6858 | 999 |
| Chr02_110093690 | Chr02 | 110093690 | 4.24675 | 23.3258 | 6867 | 999 |
| Chr02_110093692 | Chr02 | 110093692 | 4.282 | 23.6457 | 6924 | 999 |
| Chr02_110094289 | Chr02 | 110094289 | 10.0297 | 104.188 | 16218 | 999 |
| Chr02_110286336 | Chr02 | 110286336 | 9.41558 | 98.9386 | 15225 | 999 |
| Chr02_110342181 | Chr02 | 110342181 | 9.28757 | 103.985 | 15018 | 999 |
| Chr02_122389439 | Chr02 | 122389439 | 10.4756 | 124.538 | 16939 | 999 |
| Chr02_137774983 | Chr02 | 137774983 | 31.6283 | 1541.41 | 51143 | 999 |
| Chr02_137775012 | Chr02 | 137775012 | 31.6729 | 1547.22 | 51215 | 999 |
| Chr02_143643989 | Chr02 | 143643989 | 11.9845 | 279.019 | 19379 | 999 |
| Chr02_148846232 | Chr02 | 148846232 | 13.4168 | 271.711 | 21695 | 999 |
| Chr02_153460670 | Chr02 | 153460670 | 7.22696 | 59.3563 | 11686 | 999 |
| Chr02_155787121 | Chr02 | 155787121 | 5.50711 | 41.0273 | 8905 | 999 |
| Chr02_155787122 | Chr02 | 155787122 | 5.54855 | 41.8938 | 8972 | 999 |
| Chr02_155787147 | Chr02 | 155787147 | 5.5974 | 44.0538 | 9051 | 999 |
| Chr02_155787150 | Chr02 | 155787150 | 5.64131 | 44.7227 | 9122 | 999 |
| Chr02_155787152 | Chr02 | 155787152 | 5.64811 | 44.899 | 9133 | 999 |
| Chr02_159532021 | Chr02 | 159532021 | 34.5844 | 1526.78 | 55923 | 999 |
| Chr02_173618645 | Chr02 | 173618645 | 15.0878 | 266.284 | 24397 | 999 |
| Chr02_173618647 | Chr02 | 173618647 | 15.1058 | 266.778 | 24426 | 999 |
| Chr02_173618678 | Chr02 | 173618678 | 4.71367 | 31.6315 | 7622 | 999 |
| Chr02_174899514 | Chr02 | 174899514 | 11.2876 | 195.818 | 18252 | 999 |
| Chr02_174899520 | Chr02 | 174899520 | 11.2907 | 195.606 | 18257 | 999 |
| Chr02_180438041 | Chr02 | 180438041 | 9.75943 | 81.5194 | 15781 | 999 |
| Chr02_180438050 | Chr02 | 180438050 | 9.7786 | 82.347 | 15812 | 999 |
| Chr02_180438080 | Chr02 | 180438080 | 9.82004 | 82.9831 | 15879 | 999 |
| Chr02_180633892 | Chr02 | 180633892 | 5.6475 | 32.1182 | 9132 | 999 |
| Chr02_180633900 | Chr02 | 180633900 | 5.63389 | 32.0342 | 9110 | 999 |
| Chr02_186372142 | Chr02 | 186372142 | 5.1039 | 32.4583 | 8253 | 999 |
| Chr02_201026964 | Chr02 | 201026964 | 20.3717 | 682.333 | 32941 | 999 |
| Chr02_201026966 | Chr02 | 201026966 | 19.8757 | 643.676 | 32139 | 999 |
| Chr02_201027024 | Chr02 | 201027024 | 20.0142 | 664.602 | 32363 | 999 |
| Chr02_202057091 | Chr02 | 202057091 | 7.79716 | 92.0269 | 12608 | 999 |
| Chr02_202057099 | Chr02 | 202057099 | 9.83426 | 157.506 | 15902 | 999 |
| Chr02_204574647 | Chr02 | 204574647 | 6.38528 | 53.1083 | 10325 | 999 |
| Chr02_230204630 | Chr02 | 230204630 | 24.7143 | 769.538 | 39963 | 999 |
| Chr02_230204654 | Chr02 | 230204654 | 24.8355 | 780.457 | 40159 | 999 |
| Chr02_231587285 | Chr02 | 231587285 | 9.93878 | 137.617 | 16071 | 999 |
| Chr02_240037179 | Chr02 | 240037179 | 6.54174 | 40.9935 | 10578 | 999 |
| Chr02_243549374 | Chr02 | 243549374 | 6.90909 | 58.3711 | 11172 | 999 |
| Chr02_244928091 | Chr02 | 244928091 | 6.3525 | 41.7358 | 10272 | 999 |
| Chr02_244928095 | Chr02 | 244928095 | 6.33148 | 41.5918 | 10238 | 999 |
| Chr02_245307556 | Chr02 | 245307556 | 18.5547 | 590.44 | 30003 | 999 |
| Chr02_246193206 | Chr02 | 246193206 | 7.58503 | 63.7999 | 12265 | 999 |
| Chr02_266434152 | Chr02 | 266434152 | 6.77675 | 56.8022 | 10958 | 999 |
| Chr02_268509252 | Chr02 | 268509252 | 44.4193 | 3148.3 | 71826 | 999 |
| Chr02_279873267 | Chr02 | 279873267 | 19.517 | 570.375 | 31559 | 999 |
| Chr02_289059903 | Chr02 | 289059903 | 15.034 | 222.298 | 24310 | 999 |
| Chr02_297522914 | Chr02 | 297522914 | 14.3562 | 310.984 | 23214 | 999 |
| Chr02_297522919 | Chr02 | 297522919 | 14.2962 | 307.019 | 23117 | 999 |
| Chr02_297857487 | Chr02 | 297857487 | 24.3834 | 1020.87 | 39428 | 999 |
| Chr02_297857499 | Chr02 | 297857499 | 24.133 | 1012 | 39023 | 999 |
| Chr02_300115839 | Chr02 | 300115839 | 5.28139 | 42.3261 | 8540 | 999 |
| Chr02_300333349 | Chr02 | 300333349 | 9.20408 | 124.758 | 14883 | 999 |
| Chr02_300333365 | Chr02 | 300333365 | 9.24613 | 125.205 | 14951 | 999 |
| Chr02_300333382 | Chr02 | 300333382 | 9.17378 | 124.22 | 14834 | 999 |
| Chr02_300333392 | Chr02 | 300333392 | 8.88621 | 119.586 | 14369 | 999 |
| Chr02_300333393 | Chr02 | 300333393 | 8.86827 | 119.305 | 14340 | 999 |
| Chr02_302671643 | Chr02 | 302671643 | 20 | 423.946 | 32340 | 999 |
| Chr02_308461665 | Chr02 | 308461665 | 9.10328 | 86.3897 | 14720 | 999 |
| Chr02_312865733 | Chr02 | 312865733 | 7.49969 | 50.3393 | 12127 | 999 |
| Chr02_312889504 | Chr02 | 312889504 | 23.4533 | 843.184 | 37924 | 999 |
| Chr02_313854344 | Chr02 | 313854344 | 9.01917 | 136.959 | 14584 | 999 |
| Chr02_313854361 | Chr02 | 313854361 | 9.05442 | 139.298 | 14641 | 999 |
| Chr02_319100169 | Chr02 | 319100169 | 5.5603 | 41.5039 | 8991 | 999 |
| Chr02_319100254 | Chr02 | 319100254 | 8.43785 | 114.343 | 13644 | 999 |
| Chr02_322469593 | Chr02 | 322469593 | 13.4181 | 265.703 | 21697 | 999 |
| Chr02_322469596 | Chr02 | 322469596 | 13.4119 | 264.69 | 21687 | 999 |
| Chr02_322469630 | Chr02 | 322469630 | 13.556 | 269.084 | 21920 | 999 |
| Chr02_326737827 | Chr02 | 326737827 | 11.0532 | 166.604 | 17873 | 999 |
| Chr02_327228424 | Chr02 | 327228424 | 10.8058 | 174.632 | 17473 | 999 |
| Chr02_329266707 | Chr02 | 329266707 | 6.72542 | 54.1548 | 10875 | 999 |
| Chr02_329587268 | Chr02 | 329587268 | 9.01484 | 109.76 | 14577 | 999 |
| Chr02_333426402 | Chr02 | 333426402 | 12.4366 | 291.536 | 20110 | 999 |
| Chr02_334554273 | Chr02 | 334554273 | 8.56401 | 118.31 | 13848 | 999 |
| Chr02_334554331 | Chr02 | 334554331 | 9.66048 | 133.137 | 15621 | 999 |
| Chr02_336322362 | Chr02 | 336322362 | 16.6623 | 445.027 | 26943 | 999 |
| Chr02_336385366 | Chr02 | 336385366 | 6.26098 | 64.2994 | 10124 | 999 |
| Chr02_339681068 | Chr02 | 339681068 | 9.32282 | 90.3772 | 15075 | 999 |
| Chr02_347676942 | Chr02 | 347676942 | 7.53741 | 61.0891 | 12188 | 999 |
| Chr02_347676957 | Chr02 | 347676957 | 7.64997 | 61.797 | 12370 | 999 |
| Chr02_347677070 | Chr02 | 347677070 | 10.979 | 111.741 | 17753 | 999 |
| Chr02_350206466 | Chr02 | 350206466 | 8.31416 | 103.947 | 13444 | 999 |
| Chr02_350280582 | Chr02 | 350280582 | 7.21088 | 88.2606 | 11660 | 999 |
| Chr02_350787912 | Chr02 | 350787912 | 8.35003 | 75.7734 | 13502 | 999 |
| Chr02_358145089 | Chr02 | 358145089 | 5.99443 | 47.2914 | 9693 | 999 |
| Chr02_358562297 | Chr02 | 358562297 | 10.2882 | 150.568 | 16636 | 999 |
| Chr02_358562303 | Chr02 | 358562303 | 10.316 | 150.024 | 16681 | 999 |
| Chr02_358562307 | Chr02 | 358562307 | 10.2263 | 148.12 | 16536 | 999 |
| Chr02_358562321 | Chr02 | 358562321 | 10.2127 | 147.607 | 16514 | 999 |
| Chr02_358934040 | Chr02 | 358934040 | 15.4978 | 215.276 | 25060 | 999 |
| Chr02_360222675 | Chr02 | 360222675 | 8.44341 | 66.4363 | 13653 | 999 |
| Chr02_360222683 | Chr02 | 360222683 | 8.41929 | 65.4577 | 13614 | 999 |
| Chr02_360222685 | Chr02 | 360222685 | 8.46877 | 66.2962 | 13694 | 999 |
| Chr02_369405269 | Chr02 | 369405269 | 16.6908 | 459.944 | 26989 | 999 |
| Chr02_369405282 | Chr02 | 369405282 | 16.6506 | 457.172 | 26924 | 999 |
| Chr02_376282452 | Chr02 | 376282452 | 5.9128 | 55.7715 | 9561 | 999 |
| Chr02_379771083 | Chr02 | 379771083 | 9.89796 | 71.3343 | 16005 | 999 |
| Chr02_380052402 | Chr02 | 380052402 | 5.6444 | 46.4706 | 9127 | 999 |
| Chr02_383055488 | Chr02 | 383055488 | 9.73655 | 109.85 | 15744 | 999 |
| Chr02_383055522 | Chr02 | 383055522 | 9.83983 | 111.237 | 15911 | 999 |
| Chr02_383079122 | Chr02 | 383079122 | 12.5906 | 159.481 | 20359 | 999 |
| Chr02_383079131 | Chr02 | 383079131 | 12.6036 | 160.07 | 20380 | 999 |
| Chr02_392692621 | Chr02 | 392692621 | 12.5387 | 232.788 | 20275 | 999 |
| Chr02_393020538 | Chr02 | 393020538 | 10.2771 | 202.304 | 16618 | 999 |
| Chr02_393020553 | Chr02 | 393020553 | 10.1577 | 195.675 | 16425 | 999 |
| Chr02_400543265 | Chr02 | 400543265 | 10.8757 | 147.434 | 17586 | 999 |
| Chr02_400543268 | Chr02 | 400543268 | 10.8077 | 145.124 | 17476 | 999 |
| Chr02_403129817 | Chr02 | 403129817 | 40.5529 | 1767.89 | 65574 | 999 |
| Chr02_407966939 | Chr02 | 407966939 | 17.7532 | 573.295 | 28707 | 999 |
| Chr02_408732506 | Chr02 | 408732506 | 9.86147 | 143.507 | 15946 | 999 |
| Chr02_408732512 | Chr02 | 408732512 | 9.82004 | 141.689 | 15879 | 999 |
| Chr02_413622330 | Chr02 | 413622330 | 7.69388 | 74.4564 | 12441 | 999 |
| Chr02_413838040 | Chr02 | 413838040 | 18.7458 | 401.52 | 30312 | 999 |
| Chr02_414221744 | Chr02 | 414221744 | 6.42857 | 60.271 | 10395 | 999 |
| Chr02_425846034 | Chr02 | 425846034 | 19.227 | 714.043 | 31090 | 999 |
| Chr02_426994711 | Chr02 | 426994711 | 7.21831 | 73.724 | 11672 | 999 |
| Chr02_428713031 | Chr02 | 428713031 | 20.0056 | 391.784 | 32349 | 999 |
| Chr02_428856427 | Chr02 | 428856427 | 7.90909 | 92.5617 | 12789 | 999 |
| Chr02_428856443 | Chr02 | 428856443 | 7.90785 | 92.1951 | 12787 | 999 |
| Chr02_428856444 | Chr02 | 428856444 | 7.93445 | 93.2073 | 12830 | 999 |
| Chr02_429895126 | Chr02 | 429895126 | 11.7755 | 234.182 | 19041 | 999 |
| Chr02_429895141 | Chr02 | 429895141 | 11.7817 | 234.738 | 19051 | 999 |
| Chr02_430323827 | Chr02 | 430323827 | 10.9617 | 123.923 | 17725 | 999 |
| Chr02_431056861 | Chr02 | 431056861 | 7.96042 | 80.2868 | 12872 | 999 |
| Chr02_431247265 | Chr02 | 431247265 | 10.1528 | 108.831 | 16417 | 999 |
| Chr03_7662792 | Chr03 | 7662792 | 23.6976 | 821.956 | 38319 | 999 |
| Chr03_11829226 | Chr03 | 11829226 | 6.46691 | 64.2416 | 10457 | 999 |
| Chr03_11829265 | Chr03 | 11829265 | 6.30674 | 62.2858 | 10198 | 999 |
| Chr03_15328816 | Chr03 | 15328816 | 44.6933 | 2283.3 | 72269 | 999 |
| Chr03_19754376 | Chr03 | 19754376 | 7.31231 | 60.3622 | 11824 | 999 |
| Chr03_20367832 | Chr03 | 20367832 | 15.5411 | 394.72 | 25130 | 999 |
| Chr03_20692774 | Chr03 | 20692774 | 11.2338 | 238.352 | 18165 | 999 |
| Chr03_20692792 | Chr03 | 20692792 | 11.1942 | 235.331 | 18101 | 999 |
| Chr03_20692797 | Chr03 | 20692797 | 11.2301 | 238.837 | 18159 | 999 |
| Chr03_20692844 | Chr03 | 20692844 | 9.59802 | 189.26 | 15520 | 999 |
| Chr03_23289100 | Chr03 | 23289100 | 35.4193 | 1147.25 | 57273 | 999 |
| Chr03_23597734 | Chr03 | 23597734 | 20.2857 | 528.608 | 32802 | 999 |
| Chr03_23597740 | Chr03 | 23597740 | 20.1237 | 518.528 | 32540 | 999 |
| Chr03_23597782 | Chr03 | 23597782 | 19.0711 | 476.977 | 30838 | 999 |
| Chr03_23597785 | Chr03 | 23597785 | 19.0155 | 474.414 | 30748 | 999 |
| Chr03_23597790 | Chr03 | 23597790 | 18.8237 | 464.936 | 30438 | 999 |
| Chr03_34124787 | Chr03 | 34124787 | 14.9833 | 263.03 | 24228 | 999 |
| Chr03_34868708 | Chr03 | 34868708 | 7.28757 | 55.0404 | 11784 | 999 |
| Chr03_38465672 | Chr03 | 38465672 | 20.0476 | 533.8 | 32417 | 999 |
| Chr03_39025226 | Chr03 | 39025226 | 14.128 | 274.244 | 22845 | 999 |
| Chr03_71691670 | Chr03 | 71691670 | 8.68646 | 112.782 | 14046 | 999 |
| Chr03_76249294 | Chr03 | 76249294 | 8.78664 | 125.406 | 14208 | 999 |
| Chr03_86741358 | Chr03 | 86741358 | 7.35993 | 84.1897 | 11901 | 999 |
| Chr03_109505355 | Chr03 | 109505355 | 22.2301 | 914.976 | 35946 | 999 |
| Chr03_114623473 | Chr03 | 114623473 | 9.65615 | 142.931 | 15614 | 999 |
| Chr03_136841402 | Chr03 | 136841402 | 15.6129 | 274.2 | 25246 | 999 |
| Chr03_143853875 | Chr03 | 143853875 | 7.26036 | 50.7397 | 11740 | 999 |
| Chr03_236928352 | Chr03 | 236928352 | 11.8077 | 217.431 | 19093 | 999 |
| Chr03_261904481 | Chr03 | 261904481 | 7.88188 | 62.0943 | 12745 | 999 |
| Chr03_261904485 | Chr03 | 261904485 | 7.93012 | 63.2346 | 12823 | 999 |
| Chr03_322622125 | Chr03 | 322622125 | 10.3241 | 141.331 | 16694 | 999 |
| Chr03_326243636 | Chr03 | 326243636 | 16.4682 | 488.908 | 26629 | 999 |
| Chr03_332168512 | Chr03 | 332168512 | 38.7842 | 2372.61 | 62714 | 999 |
| Chr03_355228782 | Chr03 | 355228782 | 8.32529 | 111.982 | 13462 | 999 |
| Chr03_355228815 | Chr03 | 355228815 | 8.50526 | 118.724 | 13753 | 999 |
| Chr03_357099285 | Chr03 | 357099285 | 26.5974 | 779.122 | 43008 | 999 |
| Chr03_371710659 | Chr03 | 371710659 | 5.81385 | 48.4734 | 9401 | 999 |
| Chr03_371710662 | Chr03 | 371710662 | 5.90724 | 50.052 | 9552 | 999 |
| Chr03_407192306 | Chr03 | 407192306 | 13.4236 | 312.114 | 21706 | 999 |
| Chr03_407192330 | Chr03 | 407192330 | 13.4879 | 315.277 | 21810 | 999 |
| Chr03_416036996 | Chr03 | 416036996 | 11.5758 | 165.889 | 18718 | 999 |
| Chr03_434601979 | Chr03 | 434601979 | 16.6994 | 358.926 | 27003 | 999 |
| Chr03_437752784 | Chr03 | 437752784 | 12.0229 | 138.275 | 19441 | 999 |
| Chr03_442093584 | Chr03 | 442093584 | 9.69882 | 129.181 | 15683 | 999 |
| Chr03_442093592 | Chr03 | 442093592 | 9.75325 | 131.213 | 15771 | 999 |
| Chr03_442357006 | Chr03 | 442357006 | 21.679 | 831.995 | 35055 | 999 |
| Chr03_442357021 | Chr03 | 442357021 | 21.5541 | 822.395 | 34853 | 999 |
| Chr03_445764342 | Chr03 | 445764342 | 12.2839 | 244.134 | 19863 | 999 |
| Chr03_448702862 | Chr03 | 448702862 | 6.30365 | 51.3118 | 10193 | 999 |
| Chr03_452282310 | Chr03 | 452282310 | 15.021 | 255.068 | 24289 | 999 |
| Chr03_452282322 | Chr03 | 452282322 | 14.9647 | 252.934 | 24198 | 999 |
| Chr03_457232942 | Chr03 | 457232942 | 28.0495 | 914.361 | 45356 | 999 |
| Chr03_457233017 | Chr03 | 457233017 | 10.2022 | 100.821 | 16497 | 999 |
| Chr03_460167381 | Chr03 | 460167381 | 5.38157 | 29.7176 | 8702 | 999 |
| Chr03_460492456 | Chr03 | 460492456 | 17.8207 | 524.686 | 28816 | 999 |
| Chr03_460681402 | Chr03 | 460681402 | 13.4391 | 262.944 | 21731 | 999 |
| Chr03_474801192 | Chr03 | 474801192 | 21.4651 | 819.027 | 34709 | 999 |
| Chr03_474931531 | Chr03 | 474931531 | 4.18367 | 20.0721 | 6765 | 999 |
| Chr03_476243032 | Chr03 | 476243032 | 8.95114 | 143.574 | 14474 | 999 |
| Chr03_476243058 | Chr03 | 476243058 | 8.84787 | 144.434 | 14307 | 999 |
| Chr03_477798986 | Chr03 | 477798986 | 26.3908 | 740.091 | 42674 | 999 |
| Chr03_477971579 | Chr03 | 477971579 | 4.65925 | 34.6988 | 7534 | 999 |
| Chr03_479097157 | Chr03 | 479097157 | 9.50649 | 82.5075 | 15372 | 999 |
| Chr03_479748777 | Chr03 | 479748777 | 7.88868 | 98.3861 | 12756 | 999 |
| Chr03_480979230 | Chr03 | 480979230 | 6.02968 | 56.91 | 9750 | 999 |
| Chr03_480979249 | Chr03 | 480979249 | 6.11998 | 58.5252 | 9896 | 999 |
| Chr03_480979255 | Chr03 | 480979255 | 6.06184 | 56.99 | 9802 | 999 |
| Chr03_480979275 | Chr03 | 480979275 | 6.06184 | 57.1645 | 9802 | 999 |
| Chr03_486263710 | Chr03 | 486263710 | 11.7396 | 167.392 | 18983 | 999 |
| Chr03_486984593 | Chr03 | 486984593 | 9.09833 | 82.413 | 14712 | 999 |
| Chr03_488023679 | Chr03 | 488023679 | 27.3469 | 865.604 | 44220 | 999 |
| Chr03_488541013 | Chr03 | 488541013 | 7.27396 | 58.5703 | 11762 | 999 |
| Chr03_490542098 | Chr03 | 490542098 | 19.7446 | 530.286 | 31927 | 999 |
| Chr03_492557148 | Chr03 | 492557148 | 6.93878 | 58.1776 | 11220 | 999 |
| Chr03_493955581 | Chr03 | 493955581 | 12.9041 | 170.196 | 20866 | 999 |
| Chr03_495356596 | Chr03 | 495356596 | 20.3309 | 414.97 | 32875 | 999 |
| Chr03_495356805 | Chr03 | 495356805 | 6.09647 | 44.7543 | 9858 | 999 |
| Chr03_497512039 | Chr03 | 497512039 | 4.6444 | 29.6773 | 7510 | 999 |
| Chr03_497843660 | Chr03 | 497843660 | 7.02412 | 66.802 | 11358 | 999 |
| Chr03_497843716 | Chr03 | 497843716 | 6.84477 | 38.0644 | 11068 | 999 |
| Chr03_503866375 | Chr03 | 503866375 | 7.11936 | 43.8898 | 11512 | 999 |
| Chr03_511863512 | Chr03 | 511863512 | 6.05071 | 39.2883 | 9784 | 999 |
| Chr03_513317803 | Chr03 | 513317803 | 9.74583 | 159.61 | 15759 | 999 |
| Chr03_513590621 | Chr03 | 513590621 | 8.48361 | 79.3749 | 13718 | 999 |
| Chr03_513590639 | Chr03 | 513590639 | 8.55226 | 80.1905 | 13829 | 999 |
| Chr04_1178481 | Chr04 | 1178481 | 36.2536 | 2019.61 | 58622 | 999 |
| Chr04_1923857 | Chr04 | 1923857 | 14.4212 | 294.56 | 23319 | 999 |
| Chr04_1923894 | Chr04 | 1923894 | 46.2709 | 2718.36 | 74820 | 999 |
| Chr04_3457769 | Chr04 | 3457769 | 11.243 | 204.826 | 18180 | 999 |
| Chr04_3523799 | Chr04 | 3523799 | 10.7972 | 146.62 | 17459 | 999 |
| Chr04_6311598 | Chr04 | 6311598 | 14.5702 | 266.031 | 23560 | 999 |
| Chr04_7259369 | Chr04 | 7259369 | 15.4193 | 386.041 | 24933 | 999 |
| Chr04_8791442 | Chr04 | 8791442 | 27.9617 | 1157.86 | 45214 | 999 |
| Chr04_10316214 | Chr04 | 10316214 | 29.7211 | 843.502 | 48059 | 999 |
| Chr04_12903059 | Chr04 | 12903059 | 9.43537 | 120.786 | 15257 | 999 |
| Chr04_12955313 | Chr04 | 12955313 | 13.6778 | 250.972 | 22117 | 999 |
| Chr04_13966106 | Chr04 | 13966106 | 6.89487 | 68.2662 | 11149 | 999 |
| Chr04_16165280 | Chr04 | 16165280 | 12.2016 | 239.432 | 19730 | 999 |
| Chr04_16906109 | Chr04 | 16906109 | 10.3791 | 173.426 | 16783 | 999 |
| Chr04_20643372 | Chr04 | 20643372 | 11.2276 | 164.037 | 18155 | 999 |
| Chr04_24878274 | Chr04 | 24878274 | 12.8899 | 279.629 | 20843 | 999 |
| Chr04_36699900 | Chr04 | 36699900 | 8.03278 | 78.7062 | 12989 | 999 |
| Chr04_36699903 | Chr04 | 36699903 | 8.02659 | 78.9529 | 12979 | 999 |
| Chr04_41708502 | Chr04 | 41708502 | 9.04453 | 90.6403 | 14625 | 999 |
| Chr04_44656172 | Chr04 | 44656172 | 10.898 | 177.8 | 17622 | 999 |
| Chr04_47476593 | Chr04 | 47476593 | 11.282 | 181.701 | 18243 | 999 |
| Chr04_66380268 | Chr04 | 66380268 | 10.162 | 90.2522 | 16432 | 999 |
| Chr04_66852276 | Chr04 | 66852276 | 12.1404 | 273.256 | 19631 | 999 |
| Chr04_68516891 | Chr04 | 68516891 | 5.2987 | 44.55 | 8568 | 999 |
| Chr04_69080285 | Chr04 | 69080285 | 13.6939 | 233.949 | 22143 | 999 |
| Chr04_69080346 | Chr04 | 69080346 | 13.2313 | 227.163 | 21395 | 999 |
| Chr04_86299506 | Chr04 | 86299506 | 11.1348 | 130.504 | 18005 | 999 |
| Chr04_90623835 | Chr04 | 90623835 | 17.5022 | 333.123 | 28301 | 999 |
| Chr04_90623842 | Chr04 | 90623842 | 17.5727 | 336.164 | 28415 | 999 |
| Chr04_90623867 | Chr04 | 90623867 | 17.6494 | 337.144 | 28539 | 999 |
| Chr04_90623869 | Chr04 | 90623869 | 17.6617 | 337.145 | 28559 | 999 |
| Chr04_90623875 | Chr04 | 90623875 | 17.6537 | 337.225 | 28546 | 999 |
| Chr04_99869611 | Chr04 | 99869611 | 13.5442 | 235.645 | 21901 | 999 |
| Chr04_99870528 | Chr04 | 99870528 | 13.3624 | 237.157 | 21607 | 999 |
| Chr04_99870557 | Chr04 | 99870557 | 17.3506 | 365.939 | 28056 | 999 |
| Chr04_101007944 | Chr04 | 101007944 | 8.02288 | 116.228 | 12973 | 999 |
| Chr04_101007986 | Chr04 | 101007986 | 7.78108 | 108.66 | 12582 | 999 |
| Chr04_156282020 | Chr04 | 156282020 | 10.5399 | 192.022 | 17043 | 999 |
| Chr04_167817875 | Chr04 | 167817875 | 15.4372 | 418.483 | 24962 | 999 |
| Chr04_174143590 | Chr04 | 174143590 | 7.95918 | 74.46 | 12870 | 999 |
| Chr04_176001592 | Chr04 | 176001592 | 8.94372 | 98.745 | 14462 | 999 |
| Chr04_176514988 | Chr04 | 176514988 | 15.7483 | 328.365 | 25465 | 999 |
| Chr04_176514991 | Chr04 | 176514991 | 15.6784 | 324.997 | 25352 | 999 |
| Chr04_176515004 | Chr04 | 176515004 | 15.5096 | 316.306 | 25079 | 999 |
| Chr04_186330289 | Chr04 | 186330289 | 12.1317 | 173.179 | 19617 | 999 |
| Chr04_189003822 | Chr04 | 189003822 | 7.86333 | 71.7889 | 12715 | 999 |
| Chr04_199641364 | Chr04 | 199641364 | 9.62523 | 170.403 | 15564 | 999 |
| Chr04_208152278 | Chr04 | 208152278 | 7.75139 | 70.8787 | 12534 | 999 |
| Chr04_208470352 | Chr04 | 208470352 | 11.6735 | 104.017 | 18876 | 999 |
| Chr04_210993052 | Chr04 | 210993052 | 6.22573 | 52.4397 | 10067 | 999 |
| Chr04_211602673 | Chr04 | 211602673 | 20.0482 | 692.318 | 32418 | 999 |
| Chr04_218527076 | Chr04 | 218527076 | 5.84725 | 49.559 | 9455 | 999 |
| Chr04_220417547 | Chr04 | 220417547 | 5.77984 | 30.902 | 9346 | 999 |
| Chr04_223407782 | Chr04 | 223407782 | 8.45393 | 51.9151 | 13670 | 999 |
| Chr04_224029051 | Chr04 | 224029051 | 22.8077 | 867.545 | 36880 | 999 |
| Chr04_226342366 | Chr04 | 226342366 | 17.1812 | 481.908 | 27782 | 999 |
| Chr04_229274944 | Chr04 | 229274944 | 7.76871 | 67.6123 | 12562 | 999 |
| Chr04_245972285 | Chr04 | 245972285 | 46.517 | 2612.34 | 75218 | 999 |
| Chr04_246627694 | Chr04 | 246627694 | 9.10328 | 149.877 | 14720 | 999 |
| Chr04_248564951 | Chr04 | 248564951 | 4.76994 | 34.9495 | 7713 | 999 |
| Chr04_248840096 | Chr04 | 248840096 | 11.658 | 148.127 | 18851 | 999 |
| Chr04_248840101 | Chr04 | 248840101 | 11.6685 | 147.985 | 18868 | 999 |
| Chr04_248840108 | Chr04 | 248840108 | 11.6432 | 146.584 | 18827 | 999 |
| Chr04_250001891 | Chr04 | 250001891 | 13.0563 | 295.553 | 21112 | 999 |
| Chr04_250001921 | Chr04 | 250001921 | 13.504 | 312.496 | 21836 | 999 |
| Chr04_250001929 | Chr04 | 250001929 | 13.5881 | 316.506 | 21972 | 999 |
| Chr04_251053824 | Chr04 | 251053824 | 11.3976 | 145.502 | 18430 | 999 |
| Chr04_255125609 | Chr04 | 255125609 | 5.23871 | 37.9739 | 8471 | 999 |
| Chr04_255125610 | Chr04 | 255125610 | 5.28324 | 38.7712 | 8543 | 999 |
| Chr04_256433670 | Chr04 | 256433670 | 8.22449 | 72.3004 | 13299 | 999 |
| Chr04_257038987 | Chr04 | 257038987 | 9.95114 | 130.112 | 16091 | 999 |
| Chr04_259836329 | Chr04 | 259836329 | 20.7526 | 757.985 | 33557 | 999 |
| Chr04_260935509 | Chr04 | 260935509 | 8.91899 | 114.896 | 14422 | 999 |
| Chr04_260935522 | Chr04 | 260935522 | 9.11317 | 119.506 | 14736 | 999 |
| Chr04_260935532 | Chr04 | 260935532 | 9.0569 | 117.445 | 14645 | 999 |
| Chr04_260935533 | Chr04 | 260935533 | 9.06617 | 117.693 | 14660 | 999 |
| Chr04_260935552 | Chr04 | 260935552 | 9.06926 | 117.219 | 14665 | 999 |
| Chr04_260999160 | Chr04 | 260999160 | 29.2022 | 1315.02 | 47220 | 999 |
| Chr04_260999173 | Chr04 | 260999173 | 29.1645 | 1312.15 | 47159 | 999 |
| Chr04_261635841 | Chr04 | 261635841 | 20.4193 | 446.272 | 33018 | 999 |
| Chr04_261875652 | Chr04 | 261875652 | 10.8071 | 172.979 | 17475 | 999 |
| Chr04_261946603 | Chr04 | 261946603 | 9.40198 | 127.614 | 15203 | 999 |
| Chr04_267218805 | Chr04 | 267218805 | 8.47805 | 95.8858 | 13709 | 999 |
| Chr05_46876326 | Chr05 | 46876326 | 39.0445 | 1881.38 | 63135 | 999 |
| Chr05_46876342 | Chr05 | 46876342 | 39.1571 | 1901.93 | 63317 | 999 |
| Chr05_53664447 | Chr05 | 53664447 | 4.18244 | 23.377 | 6763 | 999 |
| Chr05_58391875 | Chr05 | 58391875 | 10.4174 | 177.289 | 16845 | 999 |
| Chr05_59051821 | Chr05 | 59051821 | 10.2733 | 150.315 | 16612 | 999 |
| Chr05_59051828 | Chr05 | 59051828 | 10.3494 | 151.99 | 16735 | 999 |
| Chr05_59051843 | Chr05 | 59051843 | 10.3803 | 153.394 | 16785 | 999 |
| Chr05_60007269 | Chr05 | 60007269 | 13.8893 | 252.301 | 22459 | 999 |
| Chr05_62981766 | Chr05 | 62981766 | 6.89858 | 45.0145 | 11155 | 999 |
| Chr05_73626244 | Chr05 | 73626244 | 16.4947 | 409.86 | 26672 | 999 |
| Chr05_73692821 | Chr05 | 73692821 | 11.355 | 221.223 | 18361 | 999 |
| Chr05_116474027 | Chr05 | 116474027 | 6.29314 | 61.9054 | 10176 | 999 |
| Chr05_116474049 | Chr05 | 116474049 | 6.36487 | 63.3284 | 10292 | 999 |
| Chr05_120473408 | Chr05 | 120473408 | 8.18182 | 49.5548 | 13230 | 999 |
| Chr05_129291431 | Chr05 | 129291431 | 15.9283 | 323.65 | 25756 | 999 |
| Chr05_154950581 | Chr05 | 154950581 | 10.8998 | 138.754 | 17625 | 999 |
| Chr05_155269040 | Chr05 | 155269040 | 25.175 | 1041.16 | 40708 | 999 |
| Chr05_207965844 | Chr05 | 207965844 | 12.6914 | 145.608 | 20522 | 999 |
| Chr05_229107912 | Chr05 | 229107912 | 6.98887 | 61.1533 | 11301 | 999 |
| Chr05_229107958 | Chr05 | 229107958 | 15.9579 | 449.525 | 25804 | 999 |
| Chr05_229444771 | Chr05 | 229444771 | 19.0291 | 616.534 | 30770 | 999 |
| Chr05_230002967 | Chr05 | 230002967 | 30.7471 | 1398.76 | 49718 | 999 |
| Chr05_231435054 | Chr05 | 231435054 | 12.1305 | 229.199 | 19615 | 999 |
| Chr05_258650016 | Chr05 | 258650016 | 9.08534 | 117.837 | 14691 | 999 |
| Chr05_259186217 | Chr05 | 259186217 | 8.57576 | 92.3991 | 13867 | 999 |
| Chr05_261602958 | Chr05 | 261602958 | 10.3803 | 195.318 | 16785 | 999 |
| Chr05_277608054 | Chr05 | 277608054 | 15.2282 | 268.504 | 24624 | 999 |
| Chr05_279695990 | Chr05 | 279695990 | 12.1039 | 123.815 | 19572 | 999 |
| Chr05_287599094 | Chr05 | 287599094 | 5.14038 | 27.5007 | 8312 | 999 |
| Chr05_287599106 | Chr05 | 287599106 | 5.1274 | 27.2697 | 8291 | 999 |
| Chr05_292182659 | Chr05 | 292182659 | 7.99938 | 60.2457 | 12935 | 999 |
| Chr05_292411789 | Chr05 | 292411789 | 7.52134 | 73.8202 | 12162 | 999 |
| Chr05_292559262 | Chr05 | 292559262 | 8.2987 | 58.0524 | 13419 | 999 |
| Chr05_292559263 | Chr05 | 292559263 | 8.32035 | 58.3268 | 13454 | 999 |
| Chr05_293089678 | Chr05 | 293089678 | 5.91651 | 52.9342 | 9567 | 999 |
| Chr05_296100302 | Chr05 | 296100302 | 11.5535 | 194.83 | 18682 | 999 |
| Chr05_312949300 | Chr05 | 312949300 | 6.20903 | 59.5503 | 10040 | 999 |
| Chr05_312949339 | Chr05 | 312949339 | 6.32715 | 61.3762 | 10231 | 999 |
| Chr05_312949350 | Chr05 | 312949350 | 6.3556 | 62.2231 | 10277 | 999 |
| Chr05_315332044 | Chr05 | 315332044 | 15.9079 | 403.329 | 25723 | 999 |
| Chr05_320731547 | Chr05 | 320731547 | 11.6865 | 275.99 | 18897 | 999 |
| Chr05_322841272 | Chr05 | 322841272 | 10.4712 | 139.333 | 16932 | 999 |
| Chr05_337482308 | Chr05 | 337482308 | 29.1045 | 1023.03 | 47062 | 999 |
| Chr05_340090775 | Chr05 | 340090775 | 8.06061 | 114.524 | 13034 | 999 |
| Chr05_340090793 | Chr05 | 340090793 | 8.00433 | 113.777 | 12943 | 999 |
| Chr05_340472783 | Chr05 | 340472783 | 6.77922 | 71.0471 | 10962 | 999 |
| Chr05_340472816 | Chr05 | 340472816 | 6.64811 | 68.5426 | 10750 | 999 |
| Chr05_347112168 | Chr05 | 347112168 | 25.739 | 1039.83 | 41620 | 999 |
| Chr05_347114556 | Chr05 | 347114556 | 14.483 | 135.413 | 23419 | 999 |
| Chr05_351573203 | Chr05 | 351573203 | 7.86766 | 61.4206 | 12722 | 999 |
| Chr05_352093538 | Chr05 | 352093538 | 12.7458 | 154.076 | 20610 | 999 |
| Chr05_357122594 | Chr05 | 357122594 | 13.4119 | 233.044 | 21687 | 999 |
| Chr05_357122615 | Chr05 | 357122615 | 14.4972 | 276.54 | 23442 | 999 |
| Chr05_357122648 | Chr05 | 357122648 | 14.6865 | 282.282 | 23748 | 999 |
| Chr05_367107527 | Chr05 | 367107527 | 14.0872 | 223.03 | 22779 | 999 |
| Chr05_371987934 | Chr05 | 371987934 | 8.47743 | 85.4093 | 13708 | 999 |
| Chr05_372997364 | Chr05 | 372997364 | 21.7526 | 705.959 | 35174 | 999 |
| Chr05_383061270 | Chr05 | 383061270 | 10.1967 | 211.349 | 16488 | 999 |
| Chr05_383234692 | Chr05 | 383234692 | 8.69079 | 54.9179 | 14053 | 999 |
| Chr05_384266439 | Chr05 | 384266439 | 7.04391 | 49.7982 | 11390 | 999 |
| Chr05_387760232 | Chr05 | 387760232 | 9.19233 | 103.248 | 14864 | 999 |
| Chr05_387760281 | Chr05 | 387760281 | 9.49845 | 107.517 | 15359 | 999 |
| Chr05_387760282 | Chr05 | 387760282 | 9.50093 | 107.522 | 15363 | 999 |
| Chr05_387804373 | Chr05 | 387804373 | 9.80025 | 95.228 | 15847 | 999 |
| Chr05_387804390 | Chr05 | 387804390 | 8.66976 | 63.3661 | 14019 | 999 |
| Chr05_387804406 | Chr05 | 387804406 | 10.3265 | 99.4552 | 16698 | 999 |
| Chr05_387804422 | Chr05 | 387804422 | 11.1385 | 114.928 | 18011 | 999 |
| Chr05_387804438 | Chr05 | 387804438 | 13.5096 | 206.3 | 21845 | 999 |
| Chr05_391016206 | Chr05 | 391016206 | 19.6691 | 704.882 | 31805 | 999 |
| Chr05_392031395 | Chr05 | 392031395 | 26.282 | 908.362 | 42498 | 999 |
| Chr05_399876506 | Chr05 | 399876506 | 7.38343 | 101.172 | 11939 | 999 |
| Chr05_408248305 | Chr05 | 408248305 | 8.24613 | 117.259 | 13334 | 999 |
| Chr05_408466858 | Chr05 | 408466858 | 32.4212 | 1745.2 | 52425 | 999 |
| Chr05_421858366 | Chr05 | 421858366 | 5.4898 | 44.4505 | 8877 | 999 |
| Chr05_434141561 | Chr05 | 434141561 | 11.8695 | 262.312 | 19193 | 999 |
| Chr06_1845863 | Chr06 | 1845863 | 8.56834 | 131.224 | 13855 | 999 |
| Chr06_6464252 | Chr06 | 6464252 | 11.2412 | 182.161 | 18177 | 999 |
| Chr06_6470454 | Chr06 | 6470454 | 15.9091 | 320.489 | 25725 | 999 |
| Chr06_6470471 | Chr06 | 6470471 | 15.9066 | 319.583 | 25721 | 999 |
| Chr06_6470472 | Chr06 | 6470472 | 15.919 | 321.74 | 25741 | 999 |
| Chr06_13361397 | Chr06 | 13361397 | 8.18738 | 64.8368 | 13239 | 999 |
| Chr06_14748223 | Chr06 | 14748223 | 19.9722 | 339.806 | 32295 | 999 |
| Chr06_16384758 | Chr06 | 16384758 | 9.06308 | 78.0208 | 14655 | 999 |
| Chr06_16384760 | Chr06 | 16384760 | 9.04205 | 77.5861 | 14621 | 999 |
| Chr06_16384764 | Chr06 | 16384764 | 9.02165 | 77.0645 | 14588 | 999 |
| Chr06_16384790 | Chr06 | 16384790 | 8.93754 | 76.066 | 14452 | 999 |
| Chr06_17196117 | Chr06 | 17196117 | 6.63698 | 67.3007 | 10732 | 999 |
| Chr06_17196132 | Chr06 | 17196132 | 6.54484 | 65.3793 | 10583 | 999 |
| Chr06_17196133 | Chr06 | 17196133 | 6.61163 | 66.3973 | 10691 | 999 |
| Chr06_22815268 | Chr06 | 22815268 | 8.30303 | 59.7447 | 13426 | 999 |
| Chr06_24582707 | Chr06 | 24582707 | 8.61843 | 70.6681 | 13936 | 999 |
| Chr06_26117788 | Chr06 | 26117788 | 15.684 | 341.205 | 25361 | 999 |
| Chr06_27073118 | Chr06 | 27073118 | 7.10575 | 67.2097 | 11490 | 999 |
| Chr06_27455598 | Chr06 | 27455598 | 15.932 | 252.729 | 25762 | 999 |
| Chr06_30082580 | Chr06 | 30082580 | 9.3055 | 134.449 | 15047 | 999 |
| Chr06_30705657 | Chr06 | 30705657 | 7.90662 | 77.2097 | 12785 | 999 |
| Chr06_30705658 | Chr06 | 30705658 | 7.78293 | 74.3309 | 12585 | 999 |
| Chr06_31988039 | Chr06 | 31988039 | 34.603 | 2128.58 | 55953 | 999 |
| Chr06_33751100 | Chr06 | 33751100 | 9.45887 | 106.52 | 15295 | 999 |
| Chr06_33751132 | Chr06 | 33751132 | 8.58689 | 85.8651 | 13885 | 999 |
| Chr06_42706615 | Chr06 | 42706615 | 5.37229 | 40.1398 | 8687 | 999 |
| Chr06_42859950 | Chr06 | 42859950 | 21.4218 | 632.49 | 34639 | 999 |
| Chr06_42865296 | Chr06 | 42865296 | 10.6271 | 74.5483 | 17184 | 999 |
| Chr06_42939372 | Chr06 | 42939372 | 8.73779 | 76.5562 | 14129 | 999 |
| Chr06_42939520 | Chr06 | 42939520 | 9.46382 | 117.208 | 15303 | 999 |
| Chr06_43658577 | Chr06 | 43658577 | 15.1286 | 197.519 | 24463 | 999 |
| Chr06_52438668 | Chr06 | 52438668 | 7.46753 | 72.0907 | 12075 | 999 |
| Chr06_59235344 | Chr06 | 59235344 | 11.2684 | 159.596 | 18221 | 999 |
| Chr06_60990093 | Chr06 | 60990093 | 7.3624 | 74.975 | 11905 | 999 |
| Chr06_66331058 | Chr06 | 66331058 | 7.95733 | 68.4369 | 12867 | 999 |
| Chr06_70891386 | Chr06 | 70891386 | 7.19728 | 72.7005 | 11638 | 999 |
| Chr06_74362732 | Chr06 | 74362732 | 8.96784 | 88.1029 | 14501 | 999 |
| Chr06_76401030 | Chr06 | 76401030 | 4.55411 | 22.6259 | 7364 | 999 |
| Chr06_76401048 | Chr06 | 76401048 | 4.6444 | 23.4941 | 7510 | 999 |
| Chr06_80876119 | Chr06 | 80876119 | 6.11379 | 51.8447 | 9886 | 999 |
| Chr06_80876155 | Chr06 | 80876155 | 5.85467 | 50.0104 | 9467 | 999 |
| Chr06_80893929 | Chr06 | 80893929 | 6.43723 | 52.4319 | 10409 | 999 |
| Chr06_83749422 | Chr06 | 83749422 | 13.3364 | 250.931 | 21565 | 999 |
| Chr06_83749431 | Chr06 | 83749431 | 13.2437 | 242.236 | 21415 | 999 |
| Chr06_84041098 | Chr06 | 84041098 | 25.1138 | 814.511 | 40609 | 999 |
| Chr06_84645675 | Chr06 | 84645675 | 5.33952 | 37.988 | 8634 | 999 |
| Chr06_84697617 | Chr06 | 84697617 | 11.5176 | 156.159 | 18624 | 999 |
| Chr06_88742902 | Chr06 | 88742902 | 9.25417 | 90.3159 | 14964 | 999 |
| Chr06_91501568 | Chr06 | 91501568 | 7.67038 | 91.7558 | 12403 | 999 |
| Chr06_98458766 | Chr06 | 98458766 | 10.9654 | 146.875 | 17731 | 999 |
| Chr06_99688405 | Chr06 | 99688405 | 4.72975 | 28.0773 | 7648 | 999 |
| Chr06_100542115 | Chr06 | 100542115 | 6.65244 | 52.5982 | 10757 | 999 |
| Chr06_100542142 | Chr06 | 100542142 | 6.71614 | 53.0326 | 10860 | 999 |
| Chr06_106369291 | Chr06 | 106369291 | 9.35931 | 76.3195 | 15134 | 999 |
| Chr06_106369293 | Chr06 | 106369293 | 9.42857 | 77.651 | 15246 | 999 |
| Chr06_118732064 | Chr06 | 118732064 | 7.68151 | 79.591 | 12421 | 999 |
| Chr06_130380357 | Chr06 | 130380357 | 6.51453 | 59.4542 | 10534 | 999 |
| Chr06_130380395 | Chr06 | 130380395 | 6.57452 | 60.7174 | 10631 | 999 |
| Chr06_132936987 | Chr06 | 132936987 | 6.70872 | 60.5531 | 10848 | 999 |
| Chr06_133040137 | Chr06 | 133040137 | 15.9011 | 379.909 | 25712 | 999 |
| Chr06_133040241 | Chr06 | 133040241 | 6.68151 | 41.3211 | 10804 | 999 |
| Chr06_151814792 | Chr06 | 151814792 | 10.9443 | 106.939 | 17697 | 999 |
| Chr06_157016846 | Chr06 | 157016846 | 11.5238 | 104.022 | 18634 | 999 |
| Chr06_183338629 | Chr06 | 183338629 | 8.75015 | 129.05 | 14149 | 999 |
| Chr06_248044770 | Chr06 | 248044770 | 5.87755 | 44.0357 | 9504 | 999 |
| Chr06_249606032 | Chr06 | 249606032 | 10.6308 | 76.462 | 17190 | 999 |
| Chr06_259203822 | Chr06 | 259203822 | 9.04391 | 77.7648 | 14624 | 999 |
| Chr06_259203823 | Chr06 | 259203823 | 8.89116 | 73.9523 | 14377 | 999 |
| Chr06_260251616 | Chr06 | 260251616 | 13.2294 | 153.148 | 21392 | 999 |
| Chr06_261576840 | Chr06 | 261576840 | 10.2096 | 94.3675 | 16509 | 999 |
| Chr06_261576843 | Chr06 | 261576843 | 10.1002 | 91.8934 | 16332 | 999 |
| Chr06_280752100 | Chr06 | 280752100 | 21.4706 | 913.638 | 34718 | 999 |
| Chr06_287473175 | Chr06 | 287473175 | 5.65306 | 40.7713 | 9141 | 999 |
| Chr06_298563718 | Chr06 | 298563718 | 14.7669 | 208.353 | 23878 | 999 |
| Chr06_300899648 | Chr06 | 300899648 | 14.4341 | 235.254 | 23340 | 999 |
| Chr06_311964605 | Chr06 | 311964605 | 5.75015 | 37.471 | 9298 | 999 |
| Chr06_319161693 | Chr06 | 319161693 | 26.6413 | 691.146 | 43079 | 999 |
| Chr06_329106097 | Chr06 | 329106097 | 13.5399 | 156.944 | 21894 | 999 |
| Chr06_336561408 | Chr06 | 336561408 | 12.3395 | 219.49 | 19953 | 999 |
| Chr06_346199360 | Chr06 | 346199360 | 11.6073 | 220.921 | 18769 | 999 |
| Chr06_347258700 | Chr06 | 347258700 | 13.9283 | 269.661 | 22522 | 999 |
| Chr06_347453597 | Chr06 | 347453597 | 23.2733 | 695.284 | 37633 | 999 |
| Chr06_347530487 | Chr06 | 347530487 | 18.1528 | 431.524 | 29353 | 999 |
| Chr06_347530512 | Chr06 | 347530512 | 18.0971 | 428.855 | 29263 | 999 |
| Chr06_350531669 | Chr06 | 350531669 | 10.5325 | 139.571 | 17031 | 999 |
| Chr06_355230765 | Chr06 | 355230765 | 13.4422 | 157.449 | 21736 | 999 |
| Chr06_356650582 | Chr06 | 356650582 | 18.8763 | 588.563 | 30523 | 999 |
| Chr06_359642133 | Chr06 | 359642133 | 10.2251 | 151.23 | 16534 | 999 |
| Chr06_359642144 | Chr06 | 359642144 | 10.1855 | 151.062 | 16470 | 999 |
| Chr06_371559969 | Chr06 | 371559969 | 7.40198 | 77.8074 | 11969 | 999 |
| Chr06_371560004 | Chr06 | 371560004 | 7.24304 | 75.288 | 11712 | 999 |
| Chr06_374478544 | Chr06 | 374478544 | 8.18367 | 97.4149 | 13233 | 999 |
| Chr06_380486983 | Chr06 | 380486983 | 8.19357 | 97.5993 | 13249 | 999 |
| Chr06_381080475 | Chr06 | 381080475 | 16.0049 | 423.674 | 25880 | 999 |
| Chr06_382340729 | Chr06 | 382340729 | 9.85281 | 89.9783 | 15932 | 999 |
| Chr06_382974975 | Chr06 | 382974975 | 6.13791 | 54.9383 | 9925 | 999 |
| Chr06_392281371 | Chr06 | 392281371 | 14.8442 | 237.179 | 24003 | 999 |
| Chr06_392281382 | Chr06 | 392281382 | 14.8361 | 236.588 | 23990 | 999 |
| Chr06_400042019 | Chr06 | 400042019 | 4.89177 | 30.0607 | 7910 | 999 |
| Chr06_410634347 | Chr06 | 410634347 | 4.5603 | 33.3443 | 7374 | 999 |
| Chr06_414494611 | Chr06 | 414494611 | 6.54917 | 73.0386 | 10590 | 999 |
| Chr06_418448708 | Chr06 | 418448708 | 5.59617 | 44.0652 | 9049 | 999 |
| Chr06_418448767 | Chr06 | 418448767 | 5.48052 | 42.1211 | 8862 | 999 |
| Chr06_424161336 | Chr06 | 424161336 | 20.4304 | 625.066 | 33036 | 999 |
| Chr06_427236070 | Chr06 | 427236070 | 8.04638 | 100.64 | 13011 | 999 |
| Chr06_435419420 | Chr06 | 435419420 | 7.02041 | 59.666 | 11352 | 999 |
| Chr06_437644549 | Chr06 | 437644549 | 5.70068 | 49.4957 | 9218 | 999 |
| Chr06_440094280 | Chr06 | 440094280 | 6.46444 | 70.0868 | 10453 | 999 |
| Chr06_445232782 | Chr06 | 445232782 | 9.07421 | 116.696 | 14673 | 999 |
| Chr06_445232815 | Chr06 | 445232815 | 9.0569 | 115.763 | 14645 | 999 |
| Chr06_453853589 | Chr06 | 453853589 | 6.74397 | 60.1968 | 10905 | 999 |
| Chr06_462092891 | Chr06 | 462092891 | 29.624 | 1233.77 | 47902 | 999 |
| Chr06_467887013 | Chr06 | 467887013 | 13.6122 | 310.912 | 22011 | 999 |
| Chr06_470694278 | Chr06 | 470694278 | 12.9443 | 187.963 | 20931 | 999 |
| Chr06_470737283 | Chr06 | 470737283 | 5.09276 | 38.453 | 8235 | 999 |
| Chr06_471561307 | Chr06 | 471561307 | 5.03401 | 36.5948 | 8140 | 999 |
| Chr06_475371190 | Chr06 | 475371190 | 12.5257 | 99.9277 | 20254 | 999 |
| Chr06_475518847 | Chr06 | 475518847 | 11.5343 | 225.68 | 18651 | 999 |
| Chr06_475604794 | Chr06 | 475604794 | 6.90662 | 65.8669 | 11168 | 999 |
| Chr06_476453073 | Chr06 | 476453073 | 8.72171 | 118.137 | 14103 | 999 |
| Chr06_477960649 | Chr06 | 477960649 | 7.18924 | 45.8652 | 11625 | 999 |
| Chr06_479152023 | Chr06 | 479152023 | 7.45516 | 60.4325 | 12055 | 999 |
| Chr06_486338804 | Chr06 | 486338804 | 7.64502 | 66.4593 | 12362 | 999 |
| Chr06_488232575 | Chr06 | 488232575 | 47.8899 | 3505.99 | 77438 | 999 |
| Chr06_490105001 | Chr06 | 490105001 | 14.906 | 464.262 | 24103 | 999 |
| Chr06_495166754 | Chr06 | 495166754 | 8.11379 | 79.9041 | 13120 | 999 |
| Chr06_495166774 | Chr06 | 495166774 | 8.15337 | 80.1881 | 13184 | 999 |
| Chr06_495166785 | Chr06 | 495166785 | 8.02783 | 77.9776 | 12981 | 999 |
| Chr06_495166793 | Chr06 | 495166793 | 8.00742 | 77.9517 | 12948 | 999 |
| Chr06_498533794 | Chr06 | 498533794 | 4.2517 | 25.8494 | 6875 | 999 |
| Chr06_498583075 | Chr06 | 498583075 | 20.8324 | 728.805 | 33686 | 999 |
| Chr06_498583123 | Chr06 | 498583123 | 20.5795 | 716.505 | 33277 | 999 |
| Chr06_499137569 | Chr06 | 499137569 | 14.8689 | 186.01 | 24043 | 999 |
| Chr06_500001320 | Chr06 | 500001320 | 7.13234 | 69.8241 | 11533 | 999 |
| Chr06_500001328 | Chr06 | 500001328 | 7.17192 | 70.864 | 11597 | 999 |
| Chr06_500001349 | Chr06 | 500001349 | 7.13482 | 70.1378 | 11537 | 999 |
| Chr06_500001356 | Chr06 | 500001356 | 7.11317 | 69.7254 | 11502 | 999 |
| Chr06_500001359 | Chr06 | 500001359 | 7.11812 | 70.155 | 11510 | 999 |
| Chr06_500106939 | Chr06 | 500106939 | 10.2678 | 137.394 | 16603 | 999 |
| Chr06_500106951 | Chr06 | 500106951 | 10.2288 | 136.367 | 16540 | 999 |
| Chr06_501159650 | Chr06 | 501159650 | 10.4848 | 154.411 | 16954 | 999 |
| Chr06_505351732 | Chr06 | 505351732 | 6.64811 | 55.3272 | 10750 | 999 |
| Chr06_523749171 | Chr06 | 523749171 | 5.57514 | 41.19 | 9015 | 999 |
| Chr06_524855644 | Chr06 | 524855644 | 9.28324 | 133.617 | 15011 | 999 |
| Chr06_535830607 | Chr06 | 535830607 | 9.51391 | 105.613 | 15384 | 999 |
| Chr06_539938321 | Chr06 | 539938321 | 6.38776 | 54.1658 | 10329 | 999 |
| Chr06_539938344 | Chr06 | 539938344 | 6.26469 | 52.4113 | 10130 | 999 |
| Chr06_540030541 | Chr06 | 540030541 | 7.45578 | 103.732 | 12056 | 999 |
| Chr06_540774592 | Chr06 | 540774592 | 18.2208 | 431.006 | 29463 | 999 |
| Chr06_542371581 | Chr06 | 542371581 | 7.29252 | 61.5586 | 11792 | 999 |
| Chr06_548060500 | Chr06 | 548060500 | 19.94 | 322.079 | 32243 | 999 |
| Chr06_550174463 | Chr06 | 550174463 | 21.9196 | 722.844 | 35444 | 999 |
| Chr06_551597953 | Chr06 | 551597953 | 10.0266 | 120.971 | 16213 | 999 |
| Chr06_555605908 | Chr06 | 555605908 | 5.69697 | 39.741 | 9212 | 999 |
| Chr06_556577741 | Chr06 | 556577741 | 9.77675 | 100.565 | 15809 | 999 |
| Chr06_556577753 | Chr06 | 556577753 | 9.79468 | 100.551 | 15838 | 999 |
| Chr06_556577764 | Chr06 | 556577764 | 9.82313 | 101.693 | 15884 | 999 |
| Chr07_908566 | Chr07 | 908566 | 7.82498 | 92.3239 | 12653 | 999 |
| Chr07_2934144 | Chr07 | 2934144 | 6.3222 | 60.1752 | 10223 | 999 |
| Chr07_9288491 | Chr07 | 9288491 | 8.67285 | 87.1633 | 14024 | 999 |
| Chr07_12897954 | Chr07 | 12897954 | 7.62585 | 76.6477 | 12331 | 999 |
| Chr07_24818244 | Chr07 | 24818244 | 35.6976 | 2361.01 | 57723 | 999 |
| Chr07_26838314 | Chr07 | 26838314 | 13.7297 | 205.827 | 22201 | 999 |
| Chr07_27970574 | Chr07 | 27970574 | 29.7749 | 1304.35 | 48146 | 999 |
| Chr07_31123350 | Chr07 | 31123350 | 8.93939 | 90.3763 | 14455 | 999 |
| Chr07_32672363 | Chr07 | 32672363 | 11.9314 | 182.592 | 19293 | 999 |
| Chr07_32672387 | Chr07 | 32672387 | 12.0025 | 183.663 | 19408 | 999 |
| Chr07_33838149 | Chr07 | 33838149 | 7.37353 | 82.197 | 11923 | 999 |
| Chr07_33865612 | Chr07 | 33865612 | 5.59307 | 48.562 | 9044 | 999 |
| Chr07_39792080 | Chr07 | 39792080 | 7.29128 | 79.5927 | 11790 | 999 |
| Chr07_44287986 | Chr07 | 44287986 | 10.9258 | 168.214 | 17667 | 999 |
| Chr07_48767125 | Chr07 | 48767125 | 12.1571 | 176.556 | 19658 | 999 |
| Chr07_52325227 | Chr07 | 52325227 | 8.61657 | 87.2947 | 13933 | 999 |
| Chr07_54335477 | Chr07 | 54335477 | 15.1138 | 350.244 | 24439 | 999 |
| Chr07_66140981 | Chr07 | 66140981 | 7.32035 | 68.7426 | 11837 | 999 |
| Chr07_66149224 | Chr07 | 66149224 | 24.475 | 480.634 | 39576 | 999 |
| Chr07_125383114 | Chr07 | 125383114 | 6.73779 | 49.2245 | 10895 | 999 |
| Chr07_133038223 | Chr07 | 133038223 | 6.01793 | 44.7416 | 9731 | 999 |
| Chr07_141954787 | Chr07 | 141954787 | 13.4533 | 240.437 | 21754 | 999 |
| Chr07_141954808 | Chr07 | 141954808 | 13.3624 | 235.918 | 21607 | 999 |
| Chr07_146953832 | Chr07 | 146953832 | 6.64688 | 56.465 | 10748 | 999 |
| Chr07_161032714 | Chr07 | 161032714 | 9.74954 | 83.4242 | 15765 | 999 |
| Chr07_161032727 | Chr07 | 161032727 | 9.6778 | 81.9759 | 15649 | 999 |
| Chr07_162110784 | Chr07 | 162110784 | 7.00186 | 34.9622 | 11322 | 999 |
| Chr07_183565298 | Chr07 | 183565298 | 11.3197 | 199.33 | 18304 | 999 |
| Chr07_183565312 | Chr07 | 183565312 | 11.9171 | 222.475 | 19270 | 999 |
| Chr07_185691589 | Chr07 | 185691589 | 15.8454 | 186.689 | 25622 | 999 |
| Chr07_206310171 | Chr07 | 206310171 | 13.9351 | 309.73 | 22533 | 999 |
| Chr07_243033980 | Chr07 | 243033980 | 14.8324 | 354.553 | 23984 | 999 |
| Chr07_273553118 | Chr07 | 273553118 | 6.98083 | 57.3171 | 11288 | 999 |
| Chr07_285099834 | Chr07 | 285099834 | 10.0235 | 208.039 | 16208 | 999 |
| Chr07_285099884 | Chr07 | 285099884 | 9.88683 | 204.952 | 15987 | 999 |
| Chr07_290810503 | Chr07 | 290810503 | 13.094 | 300.969 | 21173 | 999 |
| Chr07_292293221 | Chr07 | 292293221 | 10.7007 | 124.269 | 17303 | 999 |
| Chr07_299808599 | Chr07 | 299808599 | 9.19481 | 56.2931 | 14868 | 999 |
| Chr07_302509525 | Chr07 | 302509525 | 11.6531 | 190.961 | 18843 | 999 |
| Chr07_302509894 | Chr07 | 302509894 | 25.4391 | 1025.31 | 41135 | 999 |
| Chr07_302509933 | Chr07 | 302509933 | 26.0625 | 1115.42 | 42143 | 999 |
| Chr07_302509935 | Chr07 | 302509935 | 25.833 | 1095.92 | 41772 | 999 |
| Chr07_315344001 | Chr07 | 315344001 | 21.0315 | 602.876 | 34008 | 999 |
| Chr07_354033100 | Chr07 | 354033100 | 8.42362 | 85.8173 | 13621 | 999 |
| Chr07_361465597 | Chr07 | 361465597 | 5.57452 | 43.054 | 9014 | 999 |
| Chr07_362087790 | Chr07 | 362087790 | 10.214 | 118.533 | 16516 | 999 |
| Chr07_363608502 | Chr07 | 363608502 | 17.0074 | 455.79 | 27501 | 999 |
| Chr07_373698752 | Chr07 | 373698752 | 17.9153 | 381.856 | 28969 | 999 |
| Chr07_373770034 | Chr07 | 373770034 | 11.2517 | 101.539 | 18194 | 999 |
| Chr07_378400671 | Chr07 | 378400671 | 21.7718 | 831.528 | 35205 | 999 |
| Chr07_385666945 | Chr07 | 385666945 | 7.68893 | 60.4186 | 12433 | 999 |
| Chr07_385666951 | Chr07 | 385666951 | 7.718 | 61.1717 | 12480 | 999 |
| Chr07_388928238 | Chr07 | 388928238 | 12.9314 | 132.595 | 20910 | 999 |
| Chr07_392269958 | Chr07 | 392269958 | 7.40322 | 44.4895 | 11971 | 999 |
| Chr07_392773603 | Chr07 | 392773603 | 20.3111 | 297.499 | 32843 | 999 |
| Chr07_401249218 | Chr07 | 401249218 | 6.84848 | 69.6522 | 11074 | 999 |
| Chr07_402372332 | Chr07 | 402372332 | 10.6883 | 133.416 | 17283 | 999 |
| Chr07_404693603 | Chr07 | 404693603 | 13.4038 | 192.358 | 21674 | 999 |
| Chr07_423012711 | Chr07 | 423012711 | 7.09153 | 79.0226 | 11467 | 999 |
| Chr07_423012743 | Chr07 | 423012743 | 7.17996 | 80.5029 | 11610 | 999 |
| Chr07_423012747 | Chr07 | 423012747 | 7.17378 | 80.5187 | 11600 | 999 |
| Chr07_441284933 | Chr07 | 441284933 | 8.2919 | 115.016 | 13408 | 999 |
| Chr07_445237626 | Chr07 | 445237626 | 21.53 | 561.846 | 34814 | 999 |
| Chr07_453057424 | Chr07 | 453057424 | 7.23067 | 80.4375 | 11692 | 999 |
| Chr07_453057427 | Chr07 | 453057427 | 7.2214 | 80.4893 | 11677 | 999 |
| Chr07_453442794 | Chr07 | 453442794 | 5.63698 | 44.62 | 9115 | 999 |
| Chr07_464573813 | Chr07 | 464573813 | 30.5152 | 1403.57 | 49343 | 999 |
| Chr07_473786661 | Chr07 | 473786661 | 18.5213 | 243.622 | 29949 | 999 |
| Chr07_475218049 | Chr07 | 475218049 | 9.07545 | 109.165 | 14675 | 999 |
| Chr07_480966970 | Chr07 | 480966970 | 13.893 | 339.973 | 22465 | 999 |
| Chr07_487665920 | Chr07 | 487665920 | 14.6889 | 151.801 | 23752 | 999 |
| Chr07_488112844 | Chr07 | 488112844 | 5.68275 | 42.4494 | 9189 | 999 |
| Chr07_488781985 | Chr07 | 488781985 | 31.5368 | 1409.22 | 50995 | 999 |
| Chr07_488781994 | Chr07 | 488781994 | 31.6679 | 1425.06 | 51207 | 999 |
| Chr07_491794633 | Chr07 | 491794633 | 5.73036 | 51.6748 | 9266 | 999 |
| Chr07_491794638 | Chr07 | 491794638 | 5.71552 | 51.2198 | 9242 | 999 |
| Chr07_493427471 | Chr07 | 493427471 | 8.58318 | 79.94 | 13879 | 999 |
| Chr07_494098813 | Chr07 | 494098813 | 16.2696 | 485.091 | 26308 | 999 |
| Chr07_494235806 | Chr07 | 494235806 | 24.107 | 712.368 | 38981 | 999 |
| Chr07_498144953 | Chr07 | 498144953 | 8.0773 | 78.5256 | 13061 | 999 |
| Chr07_503474608 | Chr07 | 503474608 | 6.12925 | 52.5297 | 9911 | 999 |
| Chr07_513140840 | Chr07 | 513140840 | 7.27829 | 69.6775 | 11769 | 999 |
| Chr08_5475321 | Chr08 | 5475321 | 7.54978 | 77.2848 | 12208 | 999 |
| Chr08_20191796 | Chr08 | 20191796 | 17.7236 | 411.922 | 28659 | 999 |
| Chr08_20191803 | Chr08 | 20191803 | 17.5745 | 406.962 | 28418 | 999 |
| Chr08_21706205 | Chr08 | 21706205 | 6.63575 | 57.7837 | 10730 | 999 |
| Chr08_21725019 | Chr08 | 21725019 | 14.2789 | 180.819 | 23089 | 999 |
| Chr08_32940356 | Chr08 | 32940356 | 8.9295 | 91.8502 | 14439 | 999 |
| Chr08_33244406 | Chr08 | 33244406 | 5.70192 | 41.2292 | 9220 | 999 |
| Chr08_38498266 | Chr08 | 38498266 | 7.10513 | 78.812 | 11489 | 999 |
| Chr08_47584980 | Chr08 | 47584980 | 6.28695 | 52.6453 | 10166 | 999 |
| Chr08_47585064 | Chr08 | 47585064 | 8.94496 | 52.5137 | 14464 | 999 |
| Chr08_48079117 | Chr08 | 48079117 | 13.1027 | 141.227 | 21187 | 999 |
| Chr08_48284144 | Chr08 | 48284144 | 7.85652 | 48.8779 | 12704 | 999 |
| Chr08_49694258 | Chr08 | 49694258 | 66.8633 | 5400.28 | 108118 | 999 |
| Chr08_49694260 | Chr08 | 49694260 | 66.8015 | 5387.67 | 108018 | 999 |
| Chr08_53178877 | Chr08 | 53178877 | 12.1398 | 280.807 | 19630 | 999 |
| Chr08_53280473 | Chr08 | 53280473 | 13.5776 | 288.304 | 21955 | 999 |
| Chr08_57318485 | Chr08 | 57318485 | 9.85034 | 126.147 | 15928 | 999 |
| Chr08_57878658 | Chr08 | 57878658 | 14.3358 | 298.58 | 23181 | 999 |
| Chr08_57878684 | Chr08 | 57878684 | 14.3203 | 299.475 | 23156 | 999 |
| Chr08_57878689 | Chr08 | 57878689 | 14.2356 | 296.938 | 23019 | 999 |
| Chr08_63787073 | Chr08 | 63787073 | 31.7341 | 1635.64 | 51314 | 999 |
| Chr08_63896623 | Chr08 | 63896623 | 15.7378 | 268.623 | 25448 | 999 |
| Chr08_65898048 | Chr08 | 65898048 | 18.7149 | 380.485 | 30262 | 999 |
| Chr08_72972927 | Chr08 | 72972927 | 10.7829 | 182.54 | 17436 | 999 |
| Chr08_72972936 | Chr08 | 72972936 | 10.8033 | 182.779 | 17469 | 999 |
| Chr08_72972954 | Chr08 | 72972954 | 11.1237 | 189.225 | 17987 | 999 |
| Chr08_73828412 | Chr08 | 73828412 | 23.2418 | 1138.68 | 37582 | 999 |
| Chr08_75388896 | Chr08 | 75388896 | 26.4447 | 815.844 | 42761 | 999 |
| Chr08_77507687 | Chr08 | 77507687 | 7.80891 | 86.1881 | 12627 | 999 |
| Chr08_78570810 | Chr08 | 78570810 | 3.75758 | 15.982 | 6076 | 999 |
| Chr08_82727124 | Chr08 | 82727124 | 6.41497 | 41.2751 | 10373 | 999 |
| Chr08_82727126 | Chr08 | 82727126 | 6.41435 | 41.3864 | 10372 | 999 |
| Chr08_86168177 | Chr08 | 86168177 | 8.28757 | 72.3944 | 13401 | 999 |
| Chr08_91598746 | Chr08 | 91598746 | 6.54793 | 44.1488 | 10588 | 999 |
| Chr08_94949026 | Chr08 | 94949026 | 8.93445 | 133.117 | 14447 | 999 |
| Chr08_99247256 | Chr08 | 99247256 | 19.525 | 597.349 | 31572 | 999 |
| Chr08_121556107 | Chr08 | 121556107 | 19.5473 | 426.325 | 31608 | 999 |
| Chr08_121556108 | Chr08 | 121556108 | 19.5801 | 429.823 | 31661 | 999 |
| Chr08_122970190 | Chr08 | 122970190 | 8.22573 | 114.399 | 13301 | 999 |
| Chr08_122970216 | Chr08 | 122970216 | 7.98578 | 107.795 | 12913 | 999 |
| Chr08_123172552 | Chr08 | 123172552 | 4.70748 | 34.9794 | 7612 | 999 |
| Chr08_154416824 | Chr08 | 154416824 | 9.82251 | 69.2302 | 15883 | 999 |
| Chr08_188849111 | Chr08 | 188849111 | 7.64317 | 89.4388 | 12359 | 999 |
| Chr08_189601313 | Chr08 | 189601313 | 14.6766 | 298.242 | 23732 | 999 |
| Chr08_191575709 | Chr08 | 191575709 | 8.84354 | 93.918 | 14300 | 999 |
| Chr08_206597582 | Chr08 | 206597582 | 12.4156 | 269.195 | 20076 | 999 |
| Chr08_206597591 | Chr08 | 206597591 | 12.4873 | 272.36 | 20192 | 999 |
| Chr08_213012341 | Chr08 | 213012341 | 11.6209 | 214.726 | 18791 | 999 |
| Chr08_221426187 | Chr08 | 221426187 | 10.4719 | 167.616 | 16933 | 999 |
| Chr08_222378410 | Chr08 | 222378410 | 10.1385 | 149.999 | 16394 | 999 |
| Chr08_227445364 | Chr08 | 227445364 | 10.0544 | 126.871 | 16258 | 999 |
| Chr08_227684771 | Chr08 | 227684771 | 6.35374 | 54.7894 | 10274 | 999 |
| Chr08_238348856 | Chr08 | 238348856 | 15.2758 | 379.762 | 24701 | 999 |
| Chr08_249685614 | Chr08 | 249685614 | 19.0074 | 288.778 | 30735 | 999 |
| Chr08_249685625 | Chr08 | 249685625 | 18.9864 | 289.234 | 30701 | 999 |
| Chr08_257029536 | Chr08 | 257029536 | 7.54731 | 79.8964 | 12204 | 999 |
| Chr08_257029562 | Chr08 | 257029562 | 7.60173 | 80.8834 | 12292 | 999 |
| Chr08_258900654 | Chr08 | 258900654 | 20.0179 | 620.005 | 32369 | 999 |
| Chr08_262645662 | Chr08 | 262645662 | 14.9734 | 202.787 | 24212 | 999 |
| Chr08_274203843 | Chr08 | 274203843 | 12.1948 | 237.529 | 19719 | 999 |
| Chr08_274203916 | Chr08 | 274203916 | 11.1732 | 182.808 | 18067 | 999 |
| Chr08_274906559 | Chr08 | 274906559 | 9.3525 | 156.501 | 15123 | 999 |
| Chr08_276164880 | Chr08 | 276164880 | 7.64997 | 80.2561 | 12370 | 999 |
| Chr08_286257746 | Chr08 | 286257746 | 6.09338 | 32.9461 | 9853 | 999 |
| Chr08_286707814 | Chr08 | 286707814 | 6.36549 | 55.7741 | 10293 | 999 |
| Chr08_287227944 | Chr08 | 287227944 | 4.98454 | 35.5709 | 8060 | 999 |
| Chr08_287227947 | Chr08 | 287227947 | 4.99072 | 35.8075 | 8070 | 999 |
| Chr08_288607421 | Chr08 | 288607421 | 13.9239 | 376.846 | 22515 | 999 |
| Chr08_289848559 | Chr08 | 289848559 | 22.2177 | 912.868 | 35926 | 999 |
| Chr08_301076710 | Chr08 | 301076710 | 7.54051 | 91.8327 | 12193 | 999 |
| Chr08_303295541 | Chr08 | 303295541 | 16.5782 | 482.619 | 26807 | 999 |
| Chr08_303295545 | Chr08 | 303295545 | 16.6104 | 483.151 | 26859 | 999 |
| Chr08_303295577 | Chr08 | 303295577 | 16.851 | 492.504 | 27248 | 999 |
| Chr08_304775152 | Chr08 | 304775152 | 16.2585 | 355.58 | 26290 | 999 |
| Chr08_305145859 | Chr08 | 305145859 | 16.7749 | 425.507 | 27125 | 999 |
| Chr08_314996071 | Chr08 | 314996071 | 10.0637 | 162.552 | 16273 | 999 |
| Chr08_321637788 | Chr08 | 321637788 | 20.522 | 293.768 | 33184 | 999 |
| Chr08_325358772 | Chr08 | 325358772 | 12.5244 | 242.556 | 20252 | 999 |
| Chr08_325358822 | Chr08 | 325358822 | 12.1738 | 231.077 | 19685 | 999 |
| Chr08_325358833 | Chr08 | 325358833 | 11.9196 | 224.555 | 19274 | 999 |
| Chr08_331342668 | Chr08 | 331342668 | 11.4484 | 159.855 | 18512 | 999 |
| Chr08_338223298 | Chr08 | 338223298 | 9.90414 | 106.214 | 16015 | 999 |
| Chr08_349734199 | Chr08 | 349734199 | 17.9555 | 276.336 | 29034 | 999 |
| Chr08_352040323 | Chr08 | 352040323 | 10.8336 | 192.342 | 17518 | 999 |
| Chr08_358577062 | Chr08 | 358577062 | 6.67285 | 49.5915 | 10790 | 999 |
| Chr08_368715989 | Chr08 | 368715989 | 7.34694 | 75.7527 | 11880 | 999 |
| Chr08_368716016 | Chr08 | 368716016 | 4.99072 | 37.5265 | 8070 | 999 |
| Chr08_379834548 | Chr08 | 379834548 | 15.0198 | 387.935 | 24287 | 999 |
| Chr08_385209640 | Chr08 | 385209640 | 16.4731 | 236.763 | 26637 | 999 |
| Chr08_385478188 | Chr08 | 385478188 | 19.9221 | 548.239 | 32214 | 999 |
| Chr08_385478196 | Chr08 | 385478196 | 19.9629 | 547.714 | 32280 | 999 |
| Chr08_388084205 | Chr08 | 388084205 | 8.73593 | 84.2625 | 14126 | 999 |
| Chr08_392125102 | Chr08 | 392125102 | 7.39394 | 47.5322 | 11956 | 999 |
| Chr08_392399442 | Chr08 | 392399442 | 7.5337 | 73.7626 | 12182 | 999 |
| Chr08_392482726 | Chr08 | 392482726 | 6.28695 | 52.5698 | 10166 | 999 |
| Chr08_395699098 | Chr08 | 395699098 | 8.25356 | 102.058 | 13346 | 999 |
| Chr08_395699171 | Chr08 | 395699171 | 12.1033 | 198.012 | 19571 | 999 |
| Chr08_396902457 | Chr08 | 396902457 | 6.56586 | 48.4203 | 10617 | 999 |
| Chr08_408740810 | Chr08 | 408740810 | 9.94125 | 152.793 | 16075 | 999 |
| Chr08_408740814 | Chr08 | 408740814 | 9.95547 | 153.945 | 16098 | 999 |
| Chr08_408740830 | Chr08 | 408740830 | 8.74892 | 109.255 | 14147 | 999 |
| Chr08_408740859 | Chr08 | 408740859 | 8.3624 | 104.111 | 13522 | 999 |
| Chr08_414992896 | Chr08 | 414992896 | 14.423 | 177.15 | 23322 | 999 |
| Chr08_425978032 | Chr08 | 425978032 | 27.0118 | 849.072 | 43678 | 999 |
| Chr08_427762356 | Chr08 | 427762356 | 10.3451 | 154.026 | 16728 | 999 |
| Chr08_427781214 | Chr08 | 427781214 | 8.80458 | 72.0732 | 14237 | 999 |
| Chr08_430630519 | Chr08 | 430630519 | 6.8658 | 71.4281 | 11102 | 999 |
| Chr09_305318 | Chr09 | 305318 | 18.3105 | 421.688 | 29608 | 999 |
| Chr09_13405435 | Chr09 | 13405435 | 14.0767 | 375.92 | 22762 | 999 |
| Chr09_13405447 | Chr09 | 13405447 | 14.0823 | 375.202 | 22771 | 999 |
| Chr09_13405459 | Chr09 | 13405459 | 14.1676 | 382.1 | 22909 | 999 |
| Chr09_13405484 | Chr09 | 13405484 | 13.919 | 372.622 | 22507 | 999 |
| Chr09_20107913 | Chr09 | 20107913 | 11.7248 | 201.759 | 18959 | 999 |
| Chr09_31249366 | Chr09 | 31249366 | 4.52814 | 27.4709 | 7322 | 999 |
| Chr09_31620309 | Chr09 | 31620309 | 5.70439 | 48.2814 | 9224 | 999 |
| Chr09_45971848 | Chr09 | 45971848 | 7.50959 | 74.5768 | 12143 | 999 |
| Chr09_47046939 | Chr09 | 47046939 | 16.9481 | 340.857 | 27405 | 999 |
| Chr09_52724458 | Chr09 | 52724458 | 10.8942 | 199.46 | 17616 | 999 |
| Chr09_55150260 | Chr09 | 55150260 | 7.9295 | 63.7091 | 12822 | 999 |
| Chr09_58969682 | Chr09 | 58969682 | 9.13915 | 64.8748 | 14778 | 999 |
| Chr09_58969705 | Chr09 | 58969705 | 7.65801 | 41.3106 | 12383 | 999 |
| Chr09_61659477 | Chr09 | 61659477 | 8.87755 | 132.108 | 14355 | 999 |
| Chr09_61659478 | Chr09 | 61659478 | 8.84539 | 131.122 | 14303 | 999 |
| Chr09_61659484 | Chr09 | 61659484 | 8.89301 | 132.177 | 14380 | 999 |
| Chr09_61659485 | Chr09 | 61659485 | 9.99072 | 163.654 | 16155 | 999 |
| Chr09_61659497 | Chr09 | 61659497 | 11.6871 | 197.734 | 18898 | 999 |
| Chr09_62140696 | Chr09 | 62140696 | 8.59926 | 88.2452 | 13905 | 999 |
| Chr09_69448040 | Chr09 | 69448040 | 5.18862 | 35.4502 | 8390 | 999 |
| Chr09_78413028 | Chr09 | 78413028 | 8.20532 | 109.776 | 13268 | 999 |
| Chr09_78413098 | Chr09 | 78413098 | 10.4502 | 123.208 | 16898 | 999 |
| Chr09_95449721 | Chr09 | 95449721 | 10.0884 | 117.428 | 16313 | 999 |
| Chr09_95658374 | Chr09 | 95658374 | 11.5362 | 179.536 | 18654 | 999 |
| Chr09_102443198 | Chr09 | 102443198 | 7.3624 | 55.2015 | 11905 | 999 |
| Chr09_106848847 | Chr09 | 106848847 | 31.0909 | 1442.58 | 50274 | 999 |
| Chr09_108249665 | Chr09 | 108249665 | 5.99753 | 53.5371 | 9698 | 999 |
| Chr09_111606128 | Chr09 | 111606128 | 21.5176 | 758.496 | 34794 | 999 |
| Chr09_120754513 | Chr09 | 120754513 | 12.2486 | 218.087 | 19806 | 999 |
| Chr09_120754516 | Chr09 | 120754516 | 12.2492 | 217.519 | 19807 | 999 |
| Chr09_126612427 | Chr09 | 126612427 | 8.42795 | 65.3143 | 13628 | 999 |
| Chr09_126612428 | Chr09 | 126612428 | 8.44465 | 65.7013 | 13655 | 999 |
| Chr09_126612431 | Chr09 | 126612431 | 8.41868 | 65.0789 | 13613 | 999 |
| Chr09_130073698 | Chr09 | 130073698 | 8.75881 | 90.7042 | 14163 | 999 |
| Chr09_130073700 | Chr09 | 130073700 | 8.74521 | 89.9029 | 14141 | 999 |
| Chr09_130073701 | Chr09 | 130073701 | 8.77427 | 90.8444 | 14188 | 999 |
| Chr09_130073719 | Chr09 | 130073719 | 8.76005 | 90.8162 | 14165 | 999 |
| Chr09_130239013 | Chr09 | 130239013 | 6.77118 | 44.0083 | 10949 | 999 |
| Chr09_130239089 | Chr09 | 130239089 | 7.188 | 58.5141 | 11623 | 999 |
| Chr09_144134442 | Chr09 | 144134442 | 15.0829 | 199.192 | 24389 | 999 |
| Chr09_144134448 | Chr09 | 144134448 | 15.0779 | 198.935 | 24381 | 999 |
| Chr09_149740913 | Chr09 | 149740913 | 6.54236 | 60.6196 | 10579 | 999 |
| Chr09_168287488 | Chr09 | 168287488 | 10.2968 | 64.3499 | 16650 | 999 |
| Chr09_194463841 | Chr09 | 194463841 | 5.27335 | 34.3287 | 8527 | 999 |
| Chr09_232496436 | Chr09 | 232496436 | 10.6568 | 131.963 | 17232 | 999 |
| Chr09_232496453 | Chr09 | 232496453 | 11.3797 | 151.197 | 18401 | 999 |
| Chr09_253385897 | Chr09 | 253385897 | 9.01855 | 99.314 | 14583 | 999 |
| Chr09_264822858 | Chr09 | 264822858 | 11.5665 | 145.876 | 18703 | 999 |
| Chr09_265473888 | Chr09 | 265473888 | 12.0928 | 195.241 | 19554 | 999 |
| Chr09_265839242 | Chr09 | 265839242 | 11.2758 | 90.3199 | 18233 | 999 |
| Chr09_265839245 | Chr09 | 265839245 | 11.2709 | 90.7112 | 18225 | 999 |
| Chr09_315677404 | Chr09 | 315677404 | 20.9536 | 817.319 | 33882 | 999 |
| Chr09_331760101 | Chr09 | 331760101 | 25.4205 | 780.525 | 41105 | 999 |
| Chr09_366688059 | Chr09 | 366688059 | 14.2962 | 298.001 | 23117 | 999 |
| Chr09_371651931 | Chr09 | 371651931 | 8.11626 | 95.5323 | 13124 | 999 |
| Chr09_372264225 | Chr09 | 372264225 | 5.23191 | 25.453 | 8460 | 999 |
| Chr09_382927117 | Chr09 | 382927117 | 14.9623 | 261.104 | 24194 | 999 |
| Chr09_383461455 | Chr09 | 383461455 | 17.7464 | 196.765 | 28696 | 999 |
| Chr09_398988456 | Chr09 | 398988456 | 29.9913 | 835.355 | 48496 | 999 |
| Chr09_411084083 | Chr09 | 411084083 | 11.8021 | 238.472 | 19084 | 999 |
| Chr09_412614292 | Chr09 | 412614292 | 16.261 | 405.866 | 26294 | 999 |
| Chr09_412614319 | Chr09 | 412614319 | 20.1911 | 654.889 | 32649 | 999 |
| Chr09_413193702 | Chr09 | 413193702 | 7.96784 | 100.792 | 12884 | 999 |
| Chr09_423583822 | Chr09 | 423583822 | 9.57019 | 123.577 | 15475 | 999 |
| Chr09_424637998 | Chr09 | 424637998 | 6.94558 | 84.4352 | 11231 | 999 |
| Chr09_429275259 | Chr09 | 429275259 | 19.6667 | 506.288 | 31801 | 999 |
| Chr09_430664559 | Chr09 | 430664559 | 6.69388 | 72.2769 | 10824 | 999 |
| Chr09_434824556 | Chr09 | 434824556 | 7.83179 | 104.556 | 12664 | 999 |
| Chr09_437371796 | Chr09 | 437371796 | 8.80272 | 101.852 | 14234 | 999 |
| Chr09_454421369 | Chr09 | 454421369 | 7.66729 | 56.6306 | 12398 | 999 |
| Chr09_460909538 | Chr09 | 460909538 | 6.66357 | 54.7494 | 10775 | 999 |
| Chr09_460909591 | Chr09 | 460909591 | 9.5337 | 81.9817 | 15416 | 999 |
| Chr09_465080303 | Chr09 | 465080303 | 7.97464 | 86.7723 | 12895 | 999 |
| Chr09_466988322 | Chr09 | 466988322 | 19.9765 | 422.202 | 32302 | 999 |
| Chr09_466988332 | Chr09 | 466988332 | 20.0278 | 423.235 | 32385 | 999 |
| Chr09_468357702 | Chr09 | 468357702 | 12.0099 | 190.156 | 19420 | 999 |
| Chr09_468357705 | Chr09 | 468357705 | 12.0155 | 190.588 | 19429 | 999 |
| Chr09_471002560 | Chr09 | 471002560 | 12.0742 | 154.508 | 19524 | 999 |
| Chr10_1510382 | Chr10 | 1510382 | 8.20965 | 104.756 | 13275 | 999 |
| Chr10_1731599 | Chr10 | 1731599 | 8.70377 | 115.176 | 14074 | 999 |
| Chr10_8037066 | Chr10 | 8037066 | 12.0835 | 225.637 | 19539 | 999 |
| Chr10_9177743 | Chr10 | 9177743 | 10.833 | 150.367 | 17517 | 999 |
| Chr10_11010021 | Chr10 | 11010021 | 11.3377 | 129.021 | 18333 | 999 |
| Chr10_22957065 | Chr10 | 22957065 | 6.58071 | 49.2003 | 10641 | 999 |
| Chr10_22957066 | Chr10 | 22957066 | 6.57205 | 49.1497 | 10627 | 999 |
| Chr10_26886356 | Chr10 | 26886356 | 20.3043 | 505.024 | 32832 | 999 |
| Chr10_32435015 | Chr10 | 32435015 | 7.59617 | 88.2619 | 12283 | 999 |
| Chr10_39105189 | Chr10 | 39105189 | 9.01979 | 57.8994 | 14585 | 999 |
| Chr10_39145676 | Chr10 | 39145676 | 6.38404 | 35.1897 | 10323 | 999 |
| Chr10_46238472 | Chr10 | 46238472 | 9.20656 | 116.051 | 14887 | 999 |
| Chr10_56365431 | Chr10 | 56365431 | 6.43228 | 65.4956 | 10401 | 999 |
| Chr10_68757726 | Chr10 | 68757726 | 46.4119 | 3655.23 | 75048 | 999 |
| Chr10_68924908 | Chr10 | 68924908 | 7.88064 | 61.9257 | 12743 | 999 |
| Chr10_71967273 | Chr10 | 71967273 | 6.17687 | 46.866 | 9988 | 999 |
| Chr10_79975783 | Chr10 | 79975783 | 13.7365 | 337.827 | 22212 | 999 |
| Chr10_84734663 | Chr10 | 84734663 | 6.42919 | 49.6276 | 10396 | 999 |
| Chr10_94157170 | Chr10 | 94157170 | 8.87137 | 120.714 | 14345 | 999 |
| Chr10_124678849 | Chr10 | 124678849 | 18.9845 | 640.812 | 30698 | 999 |
| Chr10_124678894 | Chr10 | 124678894 | 19.0884 | 629.743 | 30866 | 999 |
| Chr10_124678898 | Chr10 | 124678898 | 17.7842 | 538.739 | 28757 | 999 |
| Chr10_124678901 | Chr10 | 124678901 | 19.1546 | 634.883 | 30973 | 999 |
| Chr10_124678904 | Chr10 | 124678904 | 19.1892 | 636.993 | 31029 | 999 |
| Chr10_128296816 | Chr10 | 128296816 | 25.4137 | 1222.02 | 41094 | 999 |
| Chr10_133814077 | Chr10 | 133814077 | 6.51701 | 31.2041 | 10538 | 999 |
| Chr10_135268749 | Chr10 | 135268749 | 7.35993 | 84.9904 | 11901 | 999 |
| Chr10_169871177 | Chr10 | 169871177 | 15.5306 | 344.364 | 25113 | 999 |
| Chr10_169871202 | Chr10 | 169871202 | 14.0959 | 278.182 | 22793 | 999 |
| Chr10_169871212 | Chr10 | 169871212 | 14.1051 | 280.258 | 22808 | 999 |
| Chr10_198587146 | Chr10 | 198587146 | 8.5906 | 49.586 | 13891 | 999 |
| Chr10_215171673 | Chr10 | 215171673 | 10.8386 | 136.93 | 17526 | 999 |
| Chr10_215171675 | Chr10 | 215171675 | 10.7669 | 134.45 | 17410 | 999 |
| Chr10_281429026 | Chr10 | 281429026 | 5.50711 | 37.8974 | 8905 | 999 |
| Chr10_281429057 | Chr10 | 281429057 | 5.54855 | 37.7441 | 8972 | 999 |
| Chr10_281429065 | Chr10 | 281429065 | 5.52195 | 37.6259 | 8929 | 999 |
| Chr10_325395008 | Chr10 | 325395008 | 16.0649 | 431.369 | 25977 | 999 |
| Chr10_328294673 | Chr10 | 328294673 | 8.22758 | 109.917 | 13304 | 999 |
| Chr10_349410986 | Chr10 | 349410986 | 6.45269 | 45.2875 | 10434 | 999 |
| Chr10_349410987 | Chr10 | 349410987 | 6.43166 | 45.1341 | 10400 | 999 |
| Chr10_349411007 | Chr10 | 349411007 | 6.43476 | 44.9328 | 10405 | 999 |
| Chr10_367470049 | Chr10 | 367470049 | 8.95424 | 59.5276 | 14479 | 999 |
| Chr10_376260563 | Chr10 | 376260563 | 10.0835 | 120.627 | 16305 | 999 |
| Chr10_382196087 | Chr10 | 382196087 | 5.6376 | 41.605 | 9116 | 999 |
| Chr10_382196098 | Chr10 | 382196098 | 5.624 | 41.939 | 9094 | 999 |
| Chr10_387643494 | Chr10 | 387643494 | 5.14657 | 36.4185 | 8322 | 999 |
| Chr10_387643503 | Chr10 | 387643503 | 5.15894 | 36.6437 | 8342 | 999 |
| Chr10_387643511 | Chr10 | 387643511 | 5.15213 | 36.5932 | 8331 | 999 |
| Chr10_391123670 | Chr10 | 391123670 | 13.7953 | 294.92 | 22307 | 999 |
| Chr10_391880353 | Chr10 | 391880353 | 7.87075 | 70.7054 | 12727 | 999 |
| Chr10_394426064 | Chr10 | 394426064 | 6.58689 | 51.6881 | 10651 | 999 |
| Chr10_394426072 | Chr10 | 394426072 | 6.55906 | 50.7553 | 10606 | 999 |
| Chr10_420414330 | Chr10 | 420414330 | 19.525 | 530.601 | 31572 | 999 |
| Chr10_426354757 | Chr10 | 426354757 | 7.29004 | 74.2914 | 11788 | 999 |
| Chr10_432706991 | Chr10 | 432706991 | 12.0965 | 171.274 | 19560 | 999 |
| Chr10_432706992 | Chr10 | 432706992 | 12.0853 | 170.024 | 19542 | 999 |
| Chr10_437976263 | Chr10 | 437976263 | 8.12307 | 100.732 | 13135 | 999 |
| Chr10_447092888 | Chr10 | 447092888 | 14.7941 | 265.024 | 23922 | 999 |
| Chr10_450806203 | Chr10 | 450806203 | 18.2387 | 318.304 | 29492 | 999 |
| Chr10_450806226 | Chr10 | 450806226 | 18.2313 | 316.955 | 29480 | 999 |
| Chr10_461530693 | Chr10 | 461530693 | 14.4459 | 344.762 | 23359 | 999 |
| Chr10_462047750 | Chr10 | 462047750 | 5.95547 | 52.3854 | 9630 | 999 |
| Chr10_466341670 | Chr10 | 466341670 | 8.72109 | 105.178 | 14102 | 999 |
| Chr10_472016832 | Chr10 | 472016832 | 15.2931 | 440.839 | 24729 | 999 |
| Chr10_477540651 | Chr10 | 477540651 | 11.0526 | 236.998 | 17872 | 999 |
| Chr10_484700108 | Chr10 | 484700108 | 5.32344 | 35.6212 | 8608 | 999 |
| Chr10_484700110 | Chr10 | 484700110 | 5.32715 | 35.7339 | 8614 | 999 |
| Chr10_485523841 | Chr10 | 485523841 | 11.9746 | 173.158 | 19363 | 999 |
| Chr10_485523843 | Chr10 | 485523843 | 12.0161 | 174.961 | 19430 | 999 |
| Chr10_485523848 | Chr10 | 485523848 | 12.5937 | 187.577 | 20364 | 999 |
| Chr10_485523879 | Chr10 | 485523879 | 12.5782 | 187.844 | 20339 | 999 |
| Chr10_485523885 | Chr10 | 485523885 | 11.7638 | 165.718 | 19022 | 999 |
| Chr10_485523886 | Chr10 | 485523886 | 11.7186 | 164.836 | 18949 | 999 |
| Chr10_491616251 | Chr10 | 491616251 | 10.7341 | 182.445 | 17357 | 999 |
| Chr10_491616254 | Chr10 | 491616254 | 10.8541 | 186.123 | 17551 | 999 |
| Chr10_491616255 | Chr10 | 491616255 | 10.8769 | 188.924 | 17588 | 999 |
| Chr10_494311213 | Chr10 | 494311213 | 10.12 | 137.051 | 16364 | 999 |
| Chr10_494311215 | Chr10 | 494311215 | 10.1787 | 139.23 | 16459 | 999 |
| Chr10_494311217 | Chr10 | 494311217 | 10.1317 | 137.19 | 16383 | 999 |
| Chr10_495683442 | Chr10 | 495683442 | 7.78169 | 80.6448 | 12583 | 999 |
| Chr10_497965322 | Chr10 | 497965322 | 14.2709 | 267.662 | 23076 | 999 |
| Chr10_497965325 | Chr10 | 497965325 | 14.0365 | 254.851 | 22697 | 999 |
| Chr10_497965329 | Chr10 | 497965329 | 14.2709 | 266.263 | 23076 | 999 |
| Chr10_501789184 | Chr10 | 501789184 | 9.93816 | 148.474 | 16070 | 999 |
| Chr10_503408984 | Chr10 | 503408984 | 7.35807 | 59.7139 | 11898 | 999 |
| Chr10_503409061 | Chr10 | 503409061 | 6.9431 | 52.7319 | 11227 | 999 |
| Chr10_503457667 | Chr10 | 503457667 | 8.49165 | 69.437 | 13731 | 999 |
| Chr10_507088417 | Chr10 | 507088417 | 33.1293 | 1936.95 | 53570 | 999 |
| Chr10_511064391 | Chr10 | 511064391 | 6.30798 | 56.9546 | 10200 | 999 |
| Chr10_511064397 | Chr10 | 511064397 | 6.29128 | 56.365 | 10173 | 999 |
| Chr10_511064400 | Chr10 | 511064400 | 6.28695 | 56.4139 | 10166 | 999 |
| Chr10_511064443 | Chr10 | 511064443 | 6.41064 | 58.3053 | 10366 | 999 |
| Chr10_512266500 | Chr10 | 512266500 | 14.5677 | 383.133 | 23556 | 999 |
| Chr11_620775 | Chr11 | 620775 | 20.428 | 468.182 | 33032 | 999 |
| Chr11_5920497 | Chr11 | 5920497 | 6.18738 | 50.7142 | 10005 | 999 |
| Chr11_5920829 | Chr11 | 5920829 | 23.7118 | 741.632 | 38342 | 999 |
| Chr11_5927927 | Chr11 | 5927927 | 9.21274 | 130.343 | 14897 | 999 |
| Chr11_6138034 | Chr11 | 6138034 | 8.3692 | 118.51 | 13533 | 999 |
| Chr11_6880122 | Chr11 | 6880122 | 11.175 | 84.3054 | 18070 | 999 |
| Chr11_6893248 | Chr11 | 6893248 | 7.74644 | 96.2451 | 12526 | 999 |
| Chr11_6893288 | Chr11 | 6893288 | 7.79716 | 101.847 | 12608 | 999 |
| Chr11_8392065 | Chr11 | 8392065 | 8.21954 | 90.7185 | 13291 | 999 |
| Chr11_9157674 | Chr11 | 9157674 | 11.1503 | 165.868 | 18030 | 999 |
| Chr11_9157715 | Chr11 | 9157715 | 12.6444 | 181.509 | 20446 | 999 |
| Chr11_9903763 | Chr11 | 9903763 | 6.99567 | 43.2853 | 11312 | 999 |
| Chr11_10926084 | Chr11 | 10926084 | 30.8763 | 1506.7 | 49927 | 999 |
| Chr11_11538300 | Chr11 | 11538300 | 35.1447 | 2108.96 | 56829 | 999 |
| Chr11_11538331 | Chr11 | 11538331 | 35.0841 | 2258.2 | 56731 | 999 |
| Chr11_12161529 | Chr11 | 12161529 | 31.3711 | 1079.99 | 50727 | 999 |
| Chr11_12161538 | Chr11 | 12161538 | 31.3259 | 1075.03 | 50654 | 999 |
| Chr11_13107376 | Chr11 | 13107376 | 9.10575 | 96.2357 | 14724 | 999 |
| Chr11_13716272 | Chr11 | 13716272 | 14.7767 | 268.239 | 23894 | 999 |
| Chr11_23032056 | Chr11 | 23032056 | 5.92084 | 55.287 | 9574 | 999 |
| Chr11_23239206 | Chr11 | 23239206 | 7.1274 | 72.3464 | 11525 | 999 |
| Chr11_23239222 | Chr11 | 23239222 | 7.16141 | 72.305 | 11580 | 999 |
| Chr11_23239231 | Chr11 | 23239231 | 7.282 | 74.4774 | 11775 | 999 |
| Chr11_23239240 | Chr11 | 23239240 | 7.30179 | 75.0858 | 11807 | 999 |
| Chr11_23239247 | Chr11 | 23239247 | 7.22882 | 73.1258 | 11689 | 999 |
| Chr11_25900856 | Chr11 | 25900856 | 8.11317 | 85.3517 | 13119 | 999 |
| Chr11_26067490 | Chr11 | 26067490 | 5.49165 | 43.9493 | 8880 | 999 |
| Chr11_28543360 | Chr11 | 28543360 | 5.06741 | 40.5778 | 8194 | 999 |
| Chr11_28574582 | Chr11 | 28574582 | 7.23377 | 98.1471 | 11697 | 999 |
| Chr11_33742410 | Chr11 | 33742410 | 9.39827 | 63.715 | 15197 | 999 |
| Chr11_34860897 | Chr11 | 34860897 | 31.7588 | 1577.25 | 51354 | 999 |
| Chr11_38219273 | Chr11 | 38219273 | 6.59431 | 49.8984 | 10663 | 999 |
| Chr11_39726852 | Chr11 | 39726852 | 4.73346 | 30.3379 | 7654 | 999 |
| Chr11_42741419 | Chr11 | 42741419 | 26.5213 | 839.321 | 42885 | 999 |
| Chr11_50974977 | Chr11 | 50974977 | 9.5368 | 94.6176 | 15421 | 999 |
| Chr11_51587854 | Chr11 | 51587854 | 18.0322 | 575.442 | 29158 | 999 |
| Chr11_59342969 | Chr11 | 59342969 | 9.15213 | 88.9905 | 14799 | 999 |
| Chr11_63757166 | Chr11 | 63757166 | 20.0736 | 626.952 | 32459 | 999 |
| Chr11_73816728 | Chr11 | 73816728 | 26.2987 | 700.165 | 42525 | 999 |
| Chr11_79081657 | Chr11 | 79081657 | 13.3686 | 249.44 | 21617 | 999 |
| Chr11_81608245 | Chr11 | 81608245 | 14.3006 | 274.773 | 23124 | 999 |
| Chr11_144497312 | Chr11 | 144497312 | 7.60359 | 47.68 | 12295 | 999 |
| Chr11_182759940 | Chr11 | 182759940 | 9.51082 | 81.5854 | 15379 | 999 |
| Chr11_189094369 | Chr11 | 189094369 | 7.40816 | 81.1068 | 11979 | 999 |
| Chr11_189094372 | Chr11 | 189094372 | 7.36487 | 79.8433 | 11909 | 999 |
| Chr11_189094422 | Chr11 | 189094422 | 7.52876 | 81.6813 | 12174 | 999 |
| Chr11_191635924 | Chr11 | 191635924 | 4.53309 | 26.9991 | 7330 | 999 |
| Chr11_191635950 | Chr11 | 191635950 | 4.34261 | 23.1127 | 7022 | 999 |
| Chr11_198149644 | Chr11 | 198149644 | 19.0414 | 619.307 | 30790 | 999 |
| Chr11_198149646 | Chr11 | 198149646 | 19.0451 | 619.486 | 30796 | 999 |
| Chr11_198149654 | Chr11 | 198149654 | 19.0309 | 618.292 | 30773 | 999 |
| Chr11_205355477 | Chr11 | 205355477 | 13.0037 | 260.282 | 21027 | 999 |
| Chr11_205978256 | Chr11 | 205978256 | 9.19975 | 118.531 | 14876 | 999 |
| Chr11_206129435 | Chr11 | 206129435 | 7.77922 | 59.4345 | 12579 | 999 |
| Chr11_206129443 | Chr11 | 206129443 | 7.84045 | 60.2703 | 12678 | 999 |
| Chr11_209007297 | Chr11 | 209007297 | 7.49907 | 47.27 | 12126 | 999 |
| Chr11_210287484 | Chr11 | 210287484 | 8.39641 | 92.6763 | 13577 | 999 |
| Chr11_210287498 | Chr11 | 210287498 | 8.28077 | 90.7986 | 13390 | 999 |
| Chr11_210287501 | Chr11 | 210287501 | 8.29437 | 91.0074 | 13412 | 999 |
| Chr11_210287505 | Chr11 | 210287505 | 8.33333 | 92.2087 | 13475 | 999 |
| Chr11_210989306 | Chr11 | 210989306 | 11.9493 | 194.541 | 19322 | 999 |
| Chr11_211700100 | Chr11 | 211700100 | 17.3828 | 400.459 | 28108 | 999 |
| Chr11_211702441 | Chr11 | 211702441 | 7.57452 | 66.0033 | 12248 | 999 |
| Chr11_212950611 | Chr11 | 212950611 | 10.2604 | 184.047 | 16591 | 999 |
| Chr11_216240491 | Chr11 | 216240491 | 6.45702 | 32.2656 | 10441 | 999 |
| Chr11_219657894 | Chr11 | 219657894 | 7.09338 | 56.9498 | 11470 | 999 |
| Chr11_219657897 | Chr11 | 219657897 | 7.09709 | 57.339 | 11476 | 999 |
| Chr11_219657920 | Chr11 | 219657920 | 7.05071 | 56.7499 | 11401 | 999 |
| Chr11_219657928 | Chr11 | 219657928 | 7.03401 | 56.8262 | 11374 | 999 |
| Chr11_225969796 | Chr11 | 225969796 | 12.7143 | 278.156 | 20559 | 999 |
| Chr11_225969807 | Chr11 | 225969807 | 12.6395 | 274.468 | 20438 | 999 |
| Chr11_227076563 | Chr11 | 227076563 | 8.98516 | 112.793 | 14529 | 999 |
| Chr11_228740879 | Chr11 | 228740879 | 20.1002 | 546.141 | 32502 | 999 |
| Chr11_229203242 | Chr11 | 229203242 | 20.1391 | 333.21 | 32565 | 999 |
| Chr11_229203327 | Chr11 | 229203327 | 7.62771 | 51.3291 | 12334 | 999 |
| Chr11_229203462 | Chr11 | 229203462 | 23.4069 | 640.286 | 37849 | 999 |
| Chr11_229736767 | Chr11 | 229736767 | 6.38343 | 58.4482 | 10322 | 999 |
| Chr11_229840987 | Chr11 | 229840987 | 5.58998 | 44.1851 | 9039 | 999 |
| Chr11_230735493 | Chr11 | 230735493 | 10.3074 | 165.817 | 16667 | 999 |
| Chr11_230818043 | Chr11 | 230818043 | 10.9412 | 176.839 | 17692 | 999 |
| Chr11_233494858 | Chr11 | 233494858 | 13.9678 | 235.452 | 22586 | 999 |
| Chr11_233494872 | Chr11 | 233494872 | 14.0482 | 235.249 | 22716 | 999 |
| Chr11_233494940 | Chr11 | 233494940 | 10.2622 | 88.8025 | 16594 | 999 |
| Chr11_233569802 | Chr11 | 233569802 | 29.6809 | 728.757 | 47994 | 999 |
| Chr11_233695399 | Chr11 | 233695399 | 17.2505 | 257.088 | 27894 | 999 |
| Chr11_234942656 | Chr11 | 234942656 | 16.5294 | 418.85 | 26728 | 999 |
| Chr11_238097236 | Chr11 | 238097236 | 4.0068 | 17.0068 | 6479 | 999 |
| Chr11_238500093 | Chr11 | 238500093 | 8.65739 | 108.333 | 13999 | 999 |
| Chr11_238500094 | Chr11 | 238500094 | 8.62832 | 107.308 | 13952 | 999 |
| Chr11_240506877 | Chr11 | 240506877 | 16.8707 | 375.327 | 27280 | 999 |
| Chr11_243816718 | Chr11 | 243816718 | 21.919 | 905.829 | 35443 | 999 |
| Chr11_245378847 | Chr11 | 245378847 | 11.1113 | 147.385 | 17967 | 999 |
| Chr11_245665632 | Chr11 | 245665632 | 5.98639 | 54.6285 | 9680 | 999 |
| Chr11_248363020 | Chr11 | 248363020 | 9.63451 | 130.881 | 15579 | 999 |
| Chr11_248363039 | Chr11 | 248363039 | 9.66914 | 130.983 | 15635 | 999 |
| Chr11_248363045 | Chr11 | 248363045 | 9.63636 | 130.343 | 15582 | 999 |
| Chr11_248363046 | Chr11 | 248363046 | 9.64502 | 130.496 | 15596 | 999 |
| Chr11_248363049 | Chr11 | 248363049 | 9.63698 | 129.714 | 15583 | 999 |
| Chr12_1201293 | Chr12 | 1201293 | 9.84477 | 103.554 | 15919 | 999 |
| Chr12_3787383 | Chr12 | 3787383 | 7.20037 | 52.8522 | 11643 | 999 |
| Chr12_5422122 | Chr12 | 5422122 | 7.4632 | 66.4295 | 12068 | 999 |
| Chr12_5422128 | Chr12 | 5422128 | 7.43352 | 65.6121 | 12020 | 999 |
| Chr12_8589631 | Chr12 | 8589631 | 11.5424 | 164.928 | 18664 | 999 |
| Chr12_11208523 | Chr12 | 11208523 | 16.107 | 208.49 | 26045 | 999 |
| Chr12_11495640 | Chr12 | 11495640 | 16.5813 | 337.668 | 26812 | 999 |
| Chr12_11495660 | Chr12 | 11495660 | 16.7409 | 345.769 | 27070 | 999 |
| Chr12_13701831 | Chr12 | 13701831 | 7.51948 | 84.4577 | 12159 | 999 |
| Chr12_15977973 | Chr12 | 15977973 | 7.95609 | 83.7858 | 12865 | 999 |
| Chr12_18986282 | Chr12 | 18986282 | 6.27273 | 60.6316 | 10143 | 999 |
| Chr12_22429849 | Chr12 | 22429849 | 5.49969 | 41.0695 | 8893 | 999 |
| Chr12_22429880 | Chr12 | 22429880 | 5.77118 | 43.0912 | 9332 | 999 |
| Chr12_22429885 | Chr12 | 22429885 | 5.73779 | 42.2777 | 9278 | 999 |
| Chr12_22429897 | Chr12 | 22429897 | 5.77056 | 42.5383 | 9331 | 999 |
| Chr12_59289113 | Chr12 | 59289113 | 24.5374 | 756.71 | 39677 | 999 |
| Chr12_76188028 | Chr12 | 76188028 | 8.88002 | 103.248 | 14359 | 999 |
| Chr12_76466631 | Chr12 | 76466631 | 11.4416 | 151.849 | 18501 | 999 |
| Chr12_76636350 | Chr12 | 76636350 | 20.6197 | 787.42 | 33342 | 999 |
| Chr12_78171793 | Chr12 | 78171793 | 7.40012 | 63.937 | 11966 | 999 |
| Chr12_78171827 | Chr12 | 78171827 | 7.27025 | 62.409 | 11756 | 999 |
| Chr12_78465433 | Chr12 | 78465433 | 25.0291 | 724.241 | 40472 | 999 |
| Chr12_87898328 | Chr12 | 87898328 | 5.12678 | 35.2048 | 8290 | 999 |
| Chr12_99261608 | Chr12 | 99261608 | 9.08658 | 118.75 | 14693 | 999 |
| Chr12_116984399 | Chr12 | 116984399 | 6.19109 | 44.5656 | 10011 | 999 |
| Chr12_122371025 | Chr12 | 122371025 | 12.0792 | 211.146 | 19532 | 999 |
| Chr12_125176316 | Chr12 | 125176316 | 6.01422 | 51.5672 | 9725 | 999 |
| Chr12_130288048 | Chr12 | 130288048 | 18.7087 | 561.16 | 30252 | 999 |
| Chr12_169452160 | Chr12 | 169452160 | 25.7409 | 1281.54 | 41623 | 999 |
| Chr12_169452161 | Chr12 | 169452161 | 18.624 | 660.443 | 30115 | 999 |
| Chr12_169452199 | Chr12 | 169452199 | 17.6729 | 589.105 | 28577 | 999 |
| Chr12_194128544 | Chr12 | 194128544 | 17.4892 | 257.093 | 28280 | 999 |
| Chr12_207927483 | Chr12 | 207927483 | 16.5009 | 361.869 | 26682 | 999 |
| Chr12_212160644 | Chr12 | 212160644 | 7.97588 | 73.9072 | 12897 | 999 |
| Chr12_237700576 | Chr12 | 237700576 | 10.1831 | 143.075 | 16466 | 999 |
| Chr12_237801588 | Chr12 | 237801588 | 16.9617 | 351.206 | 27427 | 999 |
| Chr12_257156724 | Chr12 | 257156724 | 6.35189 | 51.5723 | 10271 | 999 |
| Chr12_262258813 | Chr12 | 262258813 | 7.84601 | 64.842 | 12687 | 999 |
| Chr12_274205099 | Chr12 | 274205099 | 9.84539 | 90.08 | 15920 | 999 |
| Chr12_274205134 | Chr12 | 274205134 | 9.87199 | 90.8753 | 15963 | 999 |
| Chr12_281889110 | Chr12 | 281889110 | 9.48176 | 115.223 | 15332 | 999 |
| Chr12_283846018 | Chr12 | 283846018 | 11.7662 | 228.141 | 19026 | 999 |
| Chr12_285057211 | Chr12 | 285057211 | 7.04143 | 78.6561 | 11386 | 999 |
| Chr12_295308393 | Chr12 | 295308393 | 17.8367 | 486.805 | 28842 | 999 |
| Chr12_299949244 | Chr12 | 299949244 | 6.61039 | 42.5907 | 10689 | 999 |
| Chr12_301250756 | Chr12 | 301250756 | 4.82066 | 24.0978 | 7795 | 999 |
| Chr12_312685450 | Chr12 | 312685450 | 15.8769 | 377.255 | 25673 | 999 |
| Chr12_320550434 | Chr12 | 320550434 | 7.4867 | 64.0656 | 12106 | 999 |
| Chr12_332665747 | Chr12 | 332665747 | 9.34199 | 137.907 | 15106 | 999 |
| Chr12_333963262 | Chr12 | 333963262 | 9.58874 | 122.585 | 15505 | 999 |
| Chr12_337188891 | Chr12 | 337188891 | 6.08472 | 50.3028 | 9839 | 999 |
| Chr12_337188896 | Chr12 | 337188896 | 6.10513 | 51.0273 | 9872 | 999 |
| Chr12_341101817 | Chr12 | 341101817 | 6.18677 | 42.923 | 10004 | 999 |
| Chr12_342691933 | Chr12 | 342691933 | 15.3717 | 309.017 | 24856 | 999 |
| Chr12_343015352 | Chr12 | 343015352 | 6.87508 | 75.1973 | 11117 | 999 |
| Chr12_343015374 | Chr12 | 343015374 | 4.87322 | 33.6095 | 7880 | 999 |
| Chr12_343015384 | Chr12 | 343015384 | 7.11441 | 84.7895 | 11504 | 999 |
| Chr12_343640731 | Chr12 | 343640731 | 9.1478 | 146.007 | 14792 | 999 |
| Chr12_343640764 | Chr12 | 343640764 | 9.06679 | 144.84 | 14661 | 999 |
| Chr12_343640768 | Chr12 | 343640768 | 9.06246 | 144.486 | 14654 | 999 |
| Chr12_343640782 | Chr12 | 343640782 | 8.97959 | 142.506 | 14520 | 999 |
| Chr12_344039705 | Chr12 | 344039705 | 7.95857 | 57.4964 | 12869 | 999 |
| Chr12_344039717 | Chr12 | 344039717 | 7.96784 | 57.7465 | 12884 | 999 |
| Chr12_344164657 | Chr12 | 344164657 | 22.1293 | 575.322 | 35783 | 999 |
| Chr12_351928258 | Chr12 | 351928258 | 4.9833 | 38.7083 | 8058 | 999 |
| Chr12_351928266 | Chr12 | 351928266 | 4.89549 | 37.5157 | 7916 | 999 |
| Chr12_367040960 | Chr12 | 367040960 | 30.88 | 1595.89 | 49933 | 999 |
| Chr12_376181919 | Chr12 | 376181919 | 8.87322 | 124.48 | 14348 | 999 |
| Chr12_376181944 | Chr12 | 376181944 | 8.89425 | 124.164 | 14382 | 999 |
| Chr12_376181954 | Chr12 | 376181954 | 8.90971 | 124.718 | 14407 | 999 |
| Chr12_377713682 | Chr12 | 377713682 | 7.22449 | 68.3512 | 11682 | 999 |
| Chr12_377713704 | Chr12 | 377713704 | 7.21336 | 67.7632 | 11664 | 999 |
| Chr12_382145228 | Chr12 | 382145228 | 11.3135 | 182.926 | 18294 | 999 |
| Chr12_389983806 | Chr12 | 389983806 | 10.6252 | 141.234 | 17181 | 999 |
| Chr12_389983808 | Chr12 | 389983808 | 10.5368 | 138.253 | 17038 | 999 |
| Chr12_393240819 | Chr12 | 393240819 | 5.77613 | 47.0266 | 9340 | 999 |
| Chr12_403312315 | Chr12 | 403312315 | 17.752 | 625.776 | 28705 | 999 |
| Chr12_403312382 | Chr12 | 403312382 | 23.6673 | 585.374 | 38270 | 999 |
| Chr12_406382593 | Chr12 | 406382593 | 15.7829 | 199.1 | 25521 | 999 |
| Chr12_413651456 | Chr12 | 413651456 | 13.2344 | 254.991 | 21400 | 999 |
| Chr12_415298613 | Chr12 | 415298613 | 5.12183 | 40.1875 | 8282 | 999 |
| Chr12_415361083 | Chr12 | 415361083 | 4.86518 | 33.9261 | 7867 | 999 |
| Chr12_415657564 | Chr12 | 415657564 | 5.56215 | 41.0891 | 8994 | 999 |
| Chr12_422672688 | Chr12 | 422672688 | 12.4409 | 221.175 | 20117 | 999 |
| Chr12_429183433 | Chr12 | 429183433 | 8.38899 | 113.083 | 13565 | 999 |
| Chr12_429183458 | Chr12 | 429183458 | 8.60792 | 116.8 | 13919 | 999 |
| Chr12_429183475 | Chr12 | 429183475 | 8.69573 | 119.156 | 14061 | 999 |
| Chr12_429183477 | Chr12 | 429183477 | 8.75077 | 121.033 | 14150 | 999 |
| Chr13_1606077 | Chr13 | 1606077 | 4.6945 | 28.6628 | 7591 | 999 |
| Chr13_1870414 | Chr13 | 1870414 | 50.7032 | 2604.85 | 81987 | 999 |
| Chr13_1974819 | Chr13 | 1974819 | 5.36054 | 42.055 | 8668 | 999 |
| Chr13_2229094 | Chr13 | 2229094 | 10.3482 | 119.122 | 16733 | 999 |
| Chr13_7125199 | Chr13 | 7125199 | 26.1775 | 499.017 | 42329 | 999 |
| Chr13_7125492 | Chr13 | 7125492 | 14.5819 | 220.553 | 23579 | 999 |
| Chr13_7977100 | Chr13 | 7977100 | 6.7149 | 68.2943 | 10858 | 999 |
| Chr13_8692712 | Chr13 | 8692712 | 12.1688 | 201.723 | 19677 | 999 |
| Chr13_8739816 | Chr13 | 8739816 | 7.33271 | 60.69 | 11857 | 999 |
| Chr13_8739831 | Chr13 | 8739831 | 7.31169 | 60.0303 | 11823 | 999 |
| Chr13_8956875 | Chr13 | 8956875 | 10.9357 | 135.157 | 17683 | 999 |
| Chr13_8956910 | Chr13 | 8956910 | 12.1163 | 160.295 | 19592 | 999 |
| Chr13_8957396 | Chr13 | 8957396 | 15.9678 | 276.531 | 25820 | 999 |
| Chr13_9887200 | Chr13 | 9887200 | 14.2109 | 318.184 | 22979 | 999 |
| Chr13_11289202 | Chr13 | 11289202 | 13.2047 | 139.419 | 21352 | 999 |
| Chr13_20418330 | Chr13 | 20418330 | 5.79468 | 34.1397 | 9370 | 999 |
| Chr13_20418331 | Chr13 | 20418331 | 5.79468 | 34.2152 | 9370 | 999 |
| Chr13_20721053 | Chr13 | 20721053 | 6.12678 | 53.893 | 9907 | 999 |
| Chr13_29312865 | Chr13 | 29312865 | 9.95733 | 131.074 | 16101 | 999 |
| Chr13_29628130 | Chr13 | 29628130 | 26.1237 | 975.552 | 42242 | 999 |
| Chr13_29628145 | Chr13 | 29628145 | 26.1138 | 967.606 | 42226 | 999 |
| Chr13_30167445 | Chr13 | 30167445 | 7.28386 | 52.2863 | 11778 | 999 |
| Chr13_30695545 | Chr13 | 30695545 | 7.95795 | 70.4562 | 12868 | 999 |
| Chr13_30740013 | Chr13 | 30740013 | 5.34385 | 50.3099 | 8641 | 999 |
| Chr13_30740021 | Chr13 | 30740021 | 5.35127 | 50.207 | 8653 | 999 |
| Chr13_34031251 | Chr13 | 34031251 | 4.45578 | 25.8942 | 7205 | 999 |
| Chr13_35012685 | Chr13 | 35012685 | 14.4712 | 231.675 | 23400 | 999 |
| Chr13_53149074 | Chr13 | 53149074 | 39.786 | 1160.77 | 64334 | 999 |
| Chr13_53149116 | Chr13 | 53149116 | 36.9493 | 1324.14 | 59747 | 999 |
| Chr13_58628273 | Chr13 | 58628273 | 11.0711 | 223.807 | 17902 | 999 |
| Chr13_58962026 | Chr13 | 58962026 | 5.70192 | 45.6883 | 9220 | 999 |
| Chr13_58962048 | Chr13 | 58962048 | 5.67718 | 46.3202 | 9180 | 999 |
| Chr13_69484778 | Chr13 | 69484778 | 7.35498 | 71.869 | 11893 | 999 |
| Chr13_69484782 | Chr13 | 69484782 | 7.36364 | 71.7749 | 11907 | 999 |
| Chr13_69484789 | Chr13 | 69484789 | 7.41929 | 71.8204 | 11997 | 999 |
| Chr13_69484791 | Chr13 | 69484791 | 7.40136 | 71.5016 | 11968 | 999 |
| Chr13_69484797 | Chr13 | 69484797 | 7.42672 | 71.8549 | 12009 | 999 |
| Chr13_72288200 | Chr13 | 72288200 | 11.1651 | 180.977 | 18054 | 999 |
| Chr13_72864583 | Chr13 | 72864583 | 15.9549 | 381.695 | 25799 | 999 |
| Chr13_72864584 | Chr13 | 72864584 | 15.9382 | 379.515 | 25772 | 999 |
| Chr13_72864588 | Chr13 | 72864588 | 16.0087 | 383.277 | 25886 | 999 |
| Chr13_79700704 | Chr13 | 79700704 | 11.0989 | 221.595 | 17947 | 999 |
| Chr13_87008949 | Chr13 | 87008949 | 9.75015 | 131.795 | 15766 | 999 |
| Chr13_99597662 | Chr13 | 99597662 | 6.35003 | 64.7462 | 10268 | 999 |
| Chr13_99597691 | Chr13 | 99597691 | 6.21831 | 62.6683 | 10055 | 999 |
| Chr13_99597697 | Chr13 | 99597697 | 6.14719 | 60.8013 | 9940 | 999 |
| Chr13_99597709 | Chr13 | 99597709 | 6.06803 | 59.7219 | 9812 | 999 |
| Chr13_100698770 | Chr13 | 100698770 | 20.0489 | 693.059 | 32419 | 999 |
| Chr13_105166264 | Chr13 | 105166264 | 5.21212 | 33.2551 | 8428 | 999 |
| Chr13_105166344 | Chr13 | 105166344 | 5.17563 | 32.7798 | 8369 | 999 |
| Chr13_126281220 | Chr13 | 126281220 | 7.60544 | 61.5831 | 12298 | 999 |
| Chr13_127499040 | Chr13 | 127499040 | 22.3587 | 674.017 | 36154 | 999 |
| Chr13_127581246 | Chr13 | 127581246 | 6.8992 | 40.931 | 11156 | 999 |
| Chr13_127581249 | Chr13 | 127581249 | 6.94063 | 41.5992 | 11223 | 999 |
| Chr13_127840003 | Chr13 | 127840003 | 7.9462 | 89.0646 | 12849 | 999 |
| Chr13_127946488 | Chr13 | 127946488 | 10.9406 | 206.066 | 17691 | 999 |
| Chr13_127946490 | Chr13 | 127946490 | 9.15213 | 139.724 | 14799 | 999 |
| Chr13_128050590 | Chr13 | 128050590 | 22.504 | 821.542 | 36389 | 999 |
| Chr13_129991479 | Chr13 | 129991479 | 8.65801 | 81.9838 | 14000 | 999 |
| Chr13_131152970 | Chr13 | 131152970 | 8.49598 | 105.29 | 13738 | 999 |
| Chr13_135077392 | Chr13 | 135077392 | 13.3284 | 280.847 | 21552 | 999 |
| Chr13_135077415 | Chr13 | 135077415 | 13.0705 | 272.991 | 21135 | 999 |
| Chr13_135391195 | Chr13 | 135391195 | 11.0315 | 144.26 | 17838 | 999 |
| Chr13_135391206 | Chr13 | 135391206 | 11.1014 | 142.628 | 17951 | 999 |
| Chr13_135391210 | Chr13 | 135391210 | 11.1385 | 144.066 | 18011 | 999 |
| Chr13_162969795 | Chr13 | 162969795 | 5.09462 | 31.1105 | 8238 | 999 |
| Chr13_168736199 | Chr13 | 168736199 | 6.0167 | 32.2045 | 9729 | 999 |
| Chr13_173516181 | Chr13 | 173516181 | 9.55349 | 137.84 | 15448 | 999 |
| Chr13_174497493 | Chr13 | 174497493 | 11.0711 | 129.436 | 17902 | 999 |
| Chr13_209539856 | Chr13 | 209539856 | 24.3568 | 934.282 | 39385 | 999 |
| Chr13_214807331 | Chr13 | 214807331 | 5.99753 | 45.8948 | 9698 | 999 |
| Chr13_214970308 | Chr13 | 214970308 | 7.77427 | 58.8234 | 12571 | 999 |
| Chr13_219487073 | Chr13 | 219487073 | 10.9375 | 87.1848 | 17686 | 999 |
| Chr13_219487074 | Chr13 | 219487074 | 10.9499 | 87.6974 | 17706 | 999 |
| Chr13_219487100 | Chr13 | 219487100 | 10.8714 | 85.804 | 17579 | 999 |
| Chr13_219995626 | Chr13 | 219995626 | 4.00495 | 19.3812 | 6476 | 999 |
| Chr13_222303585 | Chr13 | 222303585 | 22.8615 | 638.217 | 36967 | 999 |
| Chr13_222303622 | Chr13 | 222303622 | 22.7953 | 635.007 | 36860 | 999 |
| Chr13_224651981 | Chr13 | 224651981 | 15.2177 | 403.511 | 24607 | 999 |
| Chr13_227609541 | Chr13 | 227609541 | 8.36735 | 92.3811 | 13530 | 999 |
| Chr13_228493062 | Chr13 | 228493062 | 24.457 | 646.527 | 39547 | 999 |
| Chr13_230311532 | Chr13 | 230311532 | 24.8528 | 938.106 | 40187 | 999 |
| Chr13_233658092 | Chr13 | 233658092 | 25.6889 | 1117.56 | 41539 | 999 |
| Chr13_234522757 | Chr13 | 234522757 | 16.7396 | 537.987 | 27068 | 999 |
| Chr13_235533793 | Chr13 | 235533793 | 8.61286 | 127.205 | 13927 | 999 |
| Chr13_235930483 | Chr13 | 235930483 | 3.88992 | 19.7874 | 6290 | 999 |
| Chr13_239849370 | Chr13 | 239849370 | 8.27706 | 72.141 | 13384 | 999 |
| Chr13_243015683 | Chr13 | 243015683 | 8.56463 | 99.7423 | 13849 | 999 |
| Chr13_246184836 | Chr13 | 246184836 | 10.3976 | 185.622 | 16813 | 999 |
| Chr13_246399984 | Chr13 | 246399984 | 10 | 95.0223 | 16170 | 999 |
| Chr13_249211867 | Chr13 | 249211867 | 10.6586 | 121.598 | 17235 | 999 |
| Chr13_250329699 | Chr13 | 250329699 | 4.40322 | 25.2606 | 7120 | 999 |
| Chr13_250449663 | Chr13 | 250449663 | 8.84045 | 91.8792 | 14295 | 999 |
| Chr13_250449674 | Chr13 | 250449674 | 8.89363 | 93.2473 | 14381 | 999 |
| Chr13_251535421 | Chr13 | 251535421 | 5.76314 | 41.3665 | 9319 | 999 |
| Chr13_256203661 | Chr13 | 256203661 | 5.12307 | 38.4211 | 8284 | 999 |
| Chr13_256206559 | Chr13 | 256206559 | 14.7978 | 345.861 | 23928 | 999 |
| Chr13_262290673 | Chr13 | 262290673 | 48.2319 | 3276.85 | 77991 | 999 |
| Chr13_267809433 | Chr13 | 267809433 | 12.8602 | 307.451 | 20795 | 999 |
| Chr13_272249896 | Chr13 | 272249896 | 20.094 | 389.359 | 32492 | 999 |
| Chr13_274191278 | Chr13 | 274191278 | 7.37353 | 79.5757 | 11923 | 999 |
| Chr13_275893141 | Chr13 | 275893141 | 8.91156 | 68.447 | 14410 | 999 |
| Chr13_289099232 | Chr13 | 289099232 | 5.47557 | 42.1283 | 8854 | 999 |
| Chr13_289155612 | Chr13 | 289155612 | 11.1985 | 188.11 | 18108 | 999 |
| Chr13_289155613 | Chr13 | 289155613 | 11.2066 | 189.012 | 18121 | 999 |
| Chr13_293291420 | Chr13 | 293291420 | 5.53309 | 35.1835 | 8947 | 999 |
| Chr13_305195389 | Chr13 | 305195389 | 20.7774 | 636.452 | 33597 | 999 |
| Chr13_311444949 | Chr13 | 311444949 | 6.73222 | 59.716 | 10886 | 999 |
| Chr13_311605151 | Chr13 | 311605151 | 12.1169 | 224.384 | 19593 | 999 |
| Chr13_314665479 | Chr13 | 314665479 | 18.4595 | 344.891 | 29849 | 999 |
| Chr13_314665484 | Chr13 | 314665484 | 18.76 | 360.909 | 30335 | 999 |
| Chr13_317432621 | Chr13 | 317432621 | 5.35436 | 38.9802 | 8658 | 999 |
| Chr13_320902463 | Chr13 | 320902463 | 11.1812 | 104.918 | 18080 | 999 |
| Chr13_321895414 | Chr13 | 321895414 | 8.03463 | 90.2067 | 12992 | 999 |
| Chr13_327317082 | Chr13 | 327317082 | 7.7786 | 100.8 | 12578 | 999 |
| Chr13_328361863 | Chr13 | 328361863 | 13.6574 | 177.064 | 22084 | 999 |
| Chr13_337684579 | Chr13 | 337684579 | 10.679 | 186.097 | 17268 | 999 |
| Chr13_338206222 | Chr13 | 338206222 | 10.7891 | 103.387 | 17446 | 999 |
| Chr13_339323932 | Chr13 | 339323932 | 14.906 | 367.88 | 24103 | 999 |
| Chr13_345158927 | Chr13 | 345158927 | 25.0606 | 1043.06 | 40523 | 999 |
| Chr13_346845665 | Chr13 | 346845665 | 8.24428 | 89.4867 | 13331 | 999 |
| Chr13_350096358 | Chr13 | 350096358 | 4.70192 | 29.4767 | 7603 | 999 |
| Chr13_351649823 | Chr13 | 351649823 | 4.72727 | 26.4423 | 7644 | 999 |
| Chr13_355660887 | Chr13 | 355660887 | 8.92517 | 107.259 | 14432 | 999 |
| Chr13_360917959 | Chr13 | 360917959 | 7.54978 | 50.02 | 12208 | 999 |
| Chr13_362372563 | Chr13 | 362372563 | 22.9388 | 642.14 | 37092 | 999 |
| Chr13_362372590 | Chr13 | 362372590 | 22.7916 | 623.288 | 36854 | 999 |
| Chr13_362372592 | Chr13 | 362372592 | 22.872 | 628.531 | 36984 | 999 |
| Chr13_365199993 | Chr13 | 365199993 | 5.81694 | 32.7103 | 9406 | 999 |
| Chr13_367659199 | Chr13 | 367659199 | 11.7835 | 80.8195 | 19054 | 999 |
| Chr13_370444119 | Chr13 | 370444119 | 17.7613 | 294.354 | 28720 | 999 |
| Chr13_372474476 | Chr13 | 372474476 | 9.2214 | 124.874 | 14911 | 999 |
| Chr13_382393775 | Chr13 | 382393775 | 7.22573 | 77.4509 | 11684 | 999 |
| Chr13_385563667 | Chr13 | 385563667 | 4.20841 | 19.7294 | 6805 | 999 |
| Chr14_3254895 | Chr14 | 3254895 | 10.7347 | 126.844 | 17358 | 999 |
| Chr14_3340566 | Chr14 | 3340566 | 4.52319 | 25.5318 | 7314 | 999 |
| Chr14_3348886 | Chr14 | 3348886 | 13.5993 | 304.394 | 21990 | 999 |
| Chr14_3866419 | Chr14 | 3866419 | 10.1565 | 117.96 | 16423 | 999 |
| Chr14_5957872 | Chr14 | 5957872 | 15.4447 | 423.049 | 24974 | 999 |
| Chr14_10856845 | Chr14 | 10856845 | 9.2282 | 100.742 | 14922 | 999 |
| Chr14_11708884 | Chr14 | 11708884 | 6.70872 | 55.0977 | 10848 | 999 |
| Chr14_11708886 | Chr14 | 11708886 | 6.65739 | 53.9989 | 10765 | 999 |
| Chr14_16578061 | Chr14 | 16578061 | 16.4137 | 321.541 | 26541 | 999 |
| Chr14_16578073 | Chr14 | 16578073 | 16.3531 | 318.075 | 26443 | 999 |
| Chr14_24486567 | Chr14 | 24486567 | 4.98639 | 23.5072 | 8063 | 999 |
| Chr14_24486568 | Chr14 | 24486568 | 4.99567 | 23.6206 | 8078 | 999 |
| Chr14_36522396 | Chr14 | 36522396 | 12.603 | 327.813 | 20379 | 999 |
| Chr14_37021902 | Chr14 | 37021902 | 14.7823 | 319.896 | 23903 | 999 |
| Chr14_45106614 | Chr14 | 45106614 | 12.6388 | 286.981 | 20437 | 999 |
| Chr14_50146679 | Chr14 | 50146679 | 5.97588 | 41.2847 | 9663 | 999 |
| Chr14_52404651 | Chr14 | 52404651 | 10.7508 | 105.669 | 17384 | 999 |
| Chr14_52424052 | Chr14 | 52424052 | 7.41744 | 67.0466 | 11994 | 999 |
| Chr14_63463237 | Chr14 | 63463237 | 7.64502 | 87.2514 | 12362 | 999 |
| Chr14_72733616 | Chr14 | 72733616 | 8.70748 | 109.731 | 14080 | 999 |
| Chr14_75804016 | Chr14 | 75804016 | 5.06803 | 34.3679 | 8195 | 999 |
| Chr14_96075549 | Chr14 | 96075549 | 20.3142 | 595.988 | 32848 | 999 |
| Chr14_111719662 | Chr14 | 111719662 | 8.76623 | 82.3513 | 14175 | 999 |
| Chr14_112810964 | Chr14 | 112810964 | 14.2808 | 321.305 | 23092 | 999 |
| Chr14_112810982 | Chr14 | 112810982 | 14.8435 | 345.627 | 24002 | 999 |
| Chr14_112810983 | Chr14 | 112810983 | 14.7372 | 339.669 | 23830 | 999 |
| Chr14_112810985 | Chr14 | 112810985 | 14.8046 | 344.032 | 23939 | 999 |
| Chr14_131229438 | Chr14 | 131229438 | 7.90414 | 99.8305 | 12781 | 999 |
| Chr14_134844233 | Chr14 | 134844233 | 8.77056 | 95.6026 | 14182 | 999 |
| Chr14_136180731 | Chr14 | 136180731 | 22.8262 | 880.163 | 36910 | 999 |
| Chr14_136180800 | Chr14 | 136180800 | 8.48485 | 97.8217 | 13720 | 999 |
| Chr14_136180814 | Chr14 | 136180814 | 9.64255 | 129.411 | 15592 | 999 |
| Chr14_150380043 | Chr14 | 150380043 | 7.28819 | 53.6236 | 11785 | 999 |
| Chr14_150390638 | Chr14 | 150390638 | 8.13791 | 44.9432 | 13159 | 999 |
| Chr14_195239200 | Chr14 | 195239200 | 8.8992 | 88.79 | 14390 | 999 |
| Chr14_216704869 | Chr14 | 216704869 | 5.81818 | 56.2479 | 9408 | 999 |
| Chr14_244993739 | Chr14 | 244993739 | 5.36425 | 38.7119 | 8674 | 999 |
| Chr14_251493501 | Chr14 | 251493501 | 8.52505 | 103.146 | 13785 | 999 |
| Chr14_251493554 | Chr14 | 251493554 | 15.9691 | 281.484 | 25822 | 999 |
| Chr14_256831443 | Chr14 | 256831443 | 8.20037 | 97.8509 | 13260 | 999 |
| Chr14_256831464 | Chr14 | 256831464 | 8.13667 | 97.5772 | 13157 | 999 |
| Chr14_266740799 | Chr14 | 266740799 | 10.2944 | 154.049 | 16646 | 999 |
| Chr14_268222376 | Chr14 | 268222376 | 7.60853 | 77.4649 | 12303 | 999 |
| Chr14_289261972 | Chr14 | 289261972 | 7.64193 | 79.3599 | 12357 | 999 |
| Chr14_304155077 | Chr14 | 304155077 | 8.1039 | 78.4063 | 13104 | 999 |
| Chr14_305606652 | Chr14 | 305606652 | 7.94682 | 68.4836 | 12850 | 999 |
| Chr14_309075456 | Chr14 | 309075456 | 10.564 | 82.0975 | 17082 | 999 |
| Chr14_311682121 | Chr14 | 311682121 | 9.76005 | 120.205 | 15782 | 999 |
| Chr14_311682129 | Chr14 | 311682129 | 9.88497 | 124.167 | 15984 | 999 |
| Chr14_311682148 | Chr14 | 311682148 | 9.83797 | 122.621 | 15908 | 999 |
| Chr14_326908638 | Chr14 | 326908638 | 5.60482 | 41.556 | 9063 | 999 |
| Chr14_328236172 | Chr14 | 328236172 | 12.5009 | 274.307 | 20214 | 999 |
| Chr14_330022747 | Chr14 | 330022747 | 8.3055 | 66.393 | 13430 | 999 |
| Chr14_335457030 | Chr14 | 335457030 | 10.9549 | 137.304 | 17714 | 999 |
| Chr14_335638266 | Chr14 | 335638266 | 5.82746 | 53.3929 | 9423 | 999 |
| Chr14_339206835 | Chr14 | 339206835 | 12.8942 | 233.263 | 20850 | 999 |
| Chr14_343028596 | Chr14 | 343028596 | 5.27335 | 36.7742 | 8527 | 999 |
| Chr14_344475867 | Chr14 | 344475867 | 4.4799 | 28.7337 | 7244 | 999 |
| Chr14_345957692 | Chr14 | 345957692 | 5.15894 | 40.0385 | 8342 | 999 |
| Chr14_345957694 | Chr14 | 345957694 | 5.14533 | 39.7233 | 8320 | 999 |
| Chr14_345957703 | Chr14 | 345957703 | 5.16821 | 40.1363 | 8357 | 999 |
| Chr14_345957716 | Chr14 | 345957716 | 5.10761 | 38.8436 | 8259 | 999 |
| Chr14_346520764 | Chr14 | 346520764 | 9.52257 | 155.777 | 15398 | 999 |
| Chr14_346934503 | Chr14 | 346934503 | 15.9796 | 366.401 | 25839 | 999 |
| Chr14_346934532 | Chr14 | 346934532 | 14.5795 | 289.869 | 23575 | 999 |
| Chr14_346934553 | Chr14 | 346934553 | 15.6382 | 351.052 | 25287 | 999 |
| Chr14_348565982 | Chr14 | 348565982 | 9.68769 | 155.591 | 15665 | 999 |
| Chr14_348566006 | Chr14 | 348566006 | 7.4397 | 87.6104 | 12030 | 999 |
| Chr14_348566039 | Chr14 | 348566039 | 7.24675 | 84.7058 | 11718 | 999 |
| Chr14_348566054 | Chr14 | 348566054 | 7.1713 | 83.9057 | 11596 | 999 |
| Chr14_352511946 | Chr14 | 352511946 | 7.04267 | 69.1362 | 11388 | 999 |
| Chr14_352512034 | Chr14 | 352512034 | 12.786 | 144.738 | 20675 | 999 |
| Chr14_362660949 | Chr14 | 362660949 | 16.214 | 424.312 | 26218 | 999 |
| Chr14_365296973 | Chr14 | 365296973 | 9.27025 | 113.91 | 14990 | 999 |
| Chr14_365393802 | Chr14 | 365393802 | 10.1868 | 121.487 | 16472 | 999 |
| Chr14_372342056 | Chr14 | 372342056 | 6.27767 | 26.7923 | 10151 | 999 |
| Chr14_381950786 | Chr14 | 381950786 | 9.36982 | 72.727 | 15151 | 999 |
| Chr14_398403874 | Chr14 | 398403874 | 13.4403 | 152.857 | 21733 | 999 |
| Chr14_407352303 | Chr14 | 407352303 | 5.09833 | 29.257 | 8244 | 999 |
| Chr14_407352304 | Chr14 | 407352304 | 5.07978 | 29.1032 | 8214 | 999 |
| Chr14_407352307 | Chr14 | 407352307 | 5.09215 | 29.2174 | 8234 | 999 |
| Chr14_411804187 | Chr14 | 411804187 | 15.8726 | 273.189 | 25666 | 999 |
| Chr14_414202850 | Chr14 | 414202850 | 21.7854 | 720.323 | 35227 | 999 |
| Chr14_425006437 | Chr14 | 425006437 | 7.27829 | 50.8445 | 11769 | 999 |
| Chr14_430424717 | Chr14 | 430424717 | 7.41682 | 58.0724 | 11993 | 999 |
| Chr14_432026827 | Chr14 | 432026827 | 14.569 | 300.926 | 23558 | 999 |
| Chr14_432026832 | Chr14 | 432026832 | 14.5714 | 302.241 | 23562 | 999 |
| Chr14_432026835 | Chr14 | 432026835 | 13.7458 | 268.249 | 22227 | 999 |
| Chr14_432026853 | Chr14 | 432026853 | 13.5114 | 263.824 | 21848 | 999 |
| Chr14_434263691 | Chr14 | 434263691 | 5.8757 | 43.6151 | 9501 | 999 |
| Chr14_437921126 | Chr14 | 437921126 | 6.20223 | 44.6082 | 10029 | 999 |
| Chr14_437921127 | Chr14 | 437921127 | 6.26778 | 46.0155 | 10135 | 999 |
| Chr14_438625459 | Chr14 | 438625459 | 5.30241 | 30.0601 | 8574 | 999 |
| Chr14_438625467 | Chr14 | 438625467 | 5.29746 | 29.9443 | 8566 | 999 |
| Chr14_442761066 | Chr14 | 442761066 | 13.2622 | 202.036 | 21445 | 999 |
| Chr14_447708583 | Chr14 | 447708583 | 11.5176 | 147.263 | 18624 | 999 |
| Chr14_447708600 | Chr14 | 447708600 | 11.8745 | 151.943 | 19201 | 999 |
| Chr14_447708628 | Chr14 | 447708628 | 11.5887 | 147.234 | 18739 | 999 |
| Chr14_447708630 | Chr14 | 447708630 | 11.7019 | 151.32 | 18922 | 999 |
| Chr14_449051812 | Chr14 | 449051812 | 21.543 | 789.625 | 34835 | 999 |
| Chr14_449051862 | Chr14 | 449051862 | 20.6512 | 732.674 | 33393 | 999 |
| Chr14_449051929 | Chr14 | 449051929 | 8.23871 | 89.417 | 13322 | 999 |
| Chr14_450608904 | Chr14 | 450608904 | 11.0315 | 179.09 | 17838 | 999 |
| Chr14_450632159 | Chr14 | 450632159 | 24.4601 | 575.235 | 39552 | 999 |
| Chr14_450632199 | Chr14 | 450632199 | 38.2028 | 814.568 | 61774 | 999 |
| Chr14_452853733 | Chr14 | 452853733 | 8.1645 | 91.619 | 13202 | 999 |
| Chr14_458185021 | Chr14 | 458185021 | 30.41 | 1169.04 | 49173 | 999 |
| Chr14_458669757 | Chr14 | 458669757 | 29.585 | 960.66 | 47839 | 999 |
| Chr14_471604078 | Chr14 | 471604078 | 27.1497 | 717.046 | 43901 | 999 |
| Chr14_473156569 | Chr14 | 473156569 | 12.5788 | 189.717 | 20340 | 999 |
| Chr14_487217113 | Chr14 | 487217113 | 9.44403 | 128.48 | 15271 | 999 |
| Chr14_489112447 | Chr14 | 489112447 | 18.9839 | 486.495 | 30697 | 999 |
| Chr14_491900799 | Chr14 | 491900799 | 8.29252 | 99.8358 | 13409 | 999 |
| Chr14_496475493 | Chr14 | 496475493 | 11.2709 | 178.107 | 18225 | 999 |
| Chr14_505543577 | Chr14 | 505543577 | 8.718 | 120.965 | 14097 | 999 |
| Chr14_510915915 | Chr14 | 510915915 | 6.79716 | 67.0331 | 10991 | 999 |
| Chr14_510915928 | Chr14 | 510915928 | 6.78973 | 66.7899 | 10979 | 999 |
| Chr14_511253440 | Chr14 | 511253440 | 7.02907 | 62.6112 | 11366 | 999 |
| Chr14_511253452 | Chr14 | 511253452 | 6.98763 | 62.5048 | 11299 | 999 |
| Chr14_511253465 | Chr14 | 511253465 | 6.86456 | 61.038 | 11100 | 999 |
| Chr14_511253478 | Chr14 | 511253478 | 6.75201 | 60.3252 | 10918 | 999 |
| Chr14_511253489 | Chr14 | 511253489 | 6.60544 | 58.6338 | 10681 | 999 |
| Chr14_513312164 | Chr14 | 513312164 | 9.70934 | 161.304 | 15700 | 999 |
| Chr14_513521123 | Chr14 | 513521123 | 14.9536 | 249.429 | 24180 | 999 |
| Chr14_519253984 | Chr14 | 519253984 | 8.03649 | 84.6812 | 12995 | 999 |
| Chr14_521991233 | Chr14 | 521991233 | 10.4682 | 135.041 | 16927 | 999 |
| Chr15_5540893 | Chr15 | 5540893 | 13.7471 | 261.458 | 22229 | 999 |
| Chr15_5540895 | Chr15 | 5540895 | 13.6772 | 258.734 | 22116 | 999 |
| Chr15_5540956 | Chr15 | 5540956 | 13.3123 | 252.99 | 21526 | 999 |
| Chr15_7950726 | Chr15 | 7950726 | 31.2888 | 1435.88 | 50594 | 999 |
| Chr15_11508076 | Chr15 | 11508076 | 10.2845 | 110.636 | 16630 | 999 |
| Chr15_12524194 | Chr15 | 12524194 | 5.96908 | 53.7367 | 9652 | 999 |
| Chr15_12882141 | Chr15 | 12882141 | 7.64873 | 62.0882 | 12368 | 999 |
| Chr15_13334960 | Chr15 | 13334960 | 8.51082 | 64.8874 | 13762 | 999 |
| Chr15_13334965 | Chr15 | 13334965 | 8.5368 | 65.453 | 13804 | 999 |
| Chr15_21307530 | Chr15 | 21307530 | 26.2771 | 1183.53 | 42490 | 999 |
| Chr15_21307551 | Chr15 | 21307551 | 26.1781 | 1174.38 | 42330 | 999 |
| Chr15_21307573 | Chr15 | 21307573 | 25.342 | 1131.26 | 40978 | 999 |
| Chr15_24533362 | Chr15 | 24533362 | 7.40383 | 80.9674 | 11972 | 999 |
| Chr15_28358616 | Chr15 | 28358616 | 5.47062 | 34.05 | 8846 | 999 |
| Chr15_28358629 | Chr15 | 28358629 | 5.41806 | 32.9712 | 8761 | 999 |
| Chr15_38609004 | Chr15 | 38609004 | 7.25788 | 64.2447 | 11736 | 999 |
| Chr15_42893999 | Chr15 | 42893999 | 8.11503 | 90.5994 | 13122 | 999 |
| Chr15_43300447 | Chr15 | 43300447 | 4.91837 | 27.4079 | 7953 | 999 |
| Chr15_61589352 | Chr15 | 61589352 | 6.20594 | 29.0634 | 10035 | 999 |
| Chr15_61589354 | Chr15 | 61589354 | 6.21212 | 29.0781 | 10045 | 999 |
| Chr15_61756705 | Chr15 | 61756705 | 8.48052 | 60.8277 | 13713 | 999 |
| Chr15_61803165 | Chr15 | 61803165 | 10.5955 | 118.589 | 17133 | 999 |
| Chr15_65143994 | Chr15 | 65143994 | 16.791 | 396.342 | 27151 | 999 |
| Chr15_67449430 | Chr15 | 67449430 | 5.67842 | 38.6391 | 9182 | 999 |
| Chr15_75201622 | Chr15 | 75201622 | 11.6382 | 229.922 | 18819 | 999 |
| Chr15_76735397 | Chr15 | 76735397 | 6.49474 | 48.286 | 10502 | 999 |
| Chr15_79177224 | Chr15 | 79177224 | 8.68275 | 99.4804 | 14040 | 999 |
| Chr15_79190826 | Chr15 | 79190826 | 17.5114 | 382.459 | 28316 | 999 |
| Chr15_91776441 | Chr15 | 91776441 | 5.21583 | 36.8909 | 8434 | 999 |
| Chr15_99567289 | Chr15 | 99567289 | 6.44774 | 39.8427 | 10426 | 999 |
| Chr15_100784782 | Chr15 | 100784782 | 7.66357 | 61.6132 | 12392 | 999 |
| Chr15_100784787 | Chr15 | 100784787 | 7.66976 | 61.5443 | 12402 | 999 |
| Chr15_103122312 | Chr15 | 103122312 | 4.20903 | 24.4192 | 6806 | 999 |
| Chr15_103122380 | Chr15 | 103122380 | 4.72665 | 29.5304 | 7643 | 999 |
| Chr15_103877466 | Chr15 | 103877466 | 14.2795 | 250.147 | 23090 | 999 |
| Chr15_103877467 | Chr15 | 103877467 | 14.1008 | 242.419 | 22801 | 999 |
| Chr15_125378235 | Chr15 | 125378235 | 10.9264 | 120.659 | 17668 | 999 |
| Chr15_138804196 | Chr15 | 138804196 | 16.4626 | 291.399 | 26620 | 999 |
| Chr15_138804233 | Chr15 | 138804233 | 8.28077 | 87.7565 | 13390 | 999 |
| Chr15_140111391 | Chr15 | 140111391 | 5.6679 | 43.7615 | 9165 | 999 |
| Chr15_141808470 | Chr15 | 141808470 | 7.66419 | 79.1724 | 12393 | 999 |
| Chr15_141808487 | Chr15 | 141808487 | 7.50526 | 74.7439 | 12136 | 999 |
| Chr15_142313388 | Chr15 | 142313388 | 4.46197 | 23.6237 | 7215 | 999 |
| Chr15_154349174 | Chr15 | 154349174 | 10.0866 | 153.645 | 16310 | 999 |
| Chr15_156274592 | Chr15 | 156274592 | 7.38404 | 70.1303 | 11940 | 999 |
| Chr15_156274594 | Chr15 | 156274594 | 7.4261 | 71.4279 | 12008 | 999 |
| Chr15_171628724 | Chr15 | 171628724 | 4.78108 | 26.4607 | 7731 | 999 |
| Chr15_189488194 | Chr15 | 189488194 | 12.462 | 203.741 | 20151 | 999 |
| Chr15_189529416 | Chr15 | 189529416 | 6.00928 | 44.3805 | 9717 | 999 |
| Chr15_211370162 | Chr15 | 211370162 | 9.88435 | 112.751 | 15983 | 999 |
| Chr15_211754052 | Chr15 | 211754052 | 15.9524 | 375.247 | 25795 | 999 |
| Chr15_211754054 | Chr15 | 211754054 | 16.1132 | 384.319 | 26055 | 999 |
| Chr15_233753443 | Chr15 | 233753443 | 14.8806 | 240.001 | 24062 | 999 |
| Chr15_233753445 | Chr15 | 233753445 | 14.9116 | 240.848 | 24112 | 999 |
| Chr15_233753451 | Chr15 | 233753451 | 14.8769 | 239.837 | 24056 | 999 |
| Chr15_233753455 | Chr15 | 233753455 | 14.8961 | 240.758 | 24087 | 999 |
| Chr15_236364927 | Chr15 | 236364927 | 4.62585 | 28.4806 | 7480 | 999 |
| Chr15_257326938 | Chr15 | 257326938 | 11.1824 | 202.779 | 18082 | 999 |
| Chr15_257326952 | Chr15 | 257326952 | 11.1571 | 202.022 | 18041 | 999 |
| Chr15_258694930 | Chr15 | 258694930 | 9.01299 | 114.519 | 14574 | 999 |
| Chr15_259418428 | Chr15 | 259418428 | 8.8893 | 100.07 | 14374 | 999 |
| Chr15_259418438 | Chr15 | 259418438 | 8.87817 | 99.4288 | 14356 | 999 |
| Chr15_259418453 | Chr15 | 259418453 | 8.76809 | 97.073 | 14178 | 999 |
| Chr15_267787327 | Chr15 | 267787327 | 14.7013 | 294.456 | 23772 | 999 |
| Chr15_315531561 | Chr15 | 315531561 | 14.5362 | 268.177 | 23505 | 999 |
| Chr15_324140245 | Chr15 | 324140245 | 7.39765 | 49.2285 | 11962 | 999 |
| Chr15_341799927 | Chr15 | 341799927 | 12.2152 | 171.471 | 19752 | 999 |
| Chr15_347817620 | Chr15 | 347817620 | 16.4768 | 233.175 | 26643 | 999 |
| Chr15_347817632 | Chr15 | 347817632 | 16.3785 | 230.05 | 26484 | 999 |
| Chr15_347817633 | Chr15 | 347817633 | 16.3797 | 230.831 | 26486 | 999 |
| Chr15_347817663 | Chr15 | 347817663 | 15.8466 | 225.624 | 25624 | 999 |
| Chr15_350600647 | Chr15 | 350600647 | 11.8126 | 202.689 | 19101 | 999 |
| Chr15_353943265 | Chr15 | 353943265 | 13.2931 | 168.185 | 21495 | 999 |
| Chr15_370867208 | Chr15 | 370867208 | 13.3859 | 257.134 | 21645 | 999 |
| Chr15_373526000 | Chr15 | 373526000 | 9.15399 | 116.554 | 14802 | 999 |
| Chr15_373526020 | Chr15 | 373526020 | 9.08782 | 115.032 | 14695 | 999 |
| Chr15_375151688 | Chr15 | 375151688 | 9.32406 | 129.413 | 15077 | 999 |
| Chr15_375151705 | Chr15 | 375151705 | 9.48609 | 132.494 | 15339 | 999 |
| Chr15_383528551 | Chr15 | 383528551 | 7.90167 | 104.449 | 12777 | 999 |
| Chr15_384414433 | Chr15 | 384414433 | 18.2331 | 553.674 | 29483 | 999 |
| Chr15_388500965 | Chr15 | 388500965 | 26.5288 | 1214.4 | 42897 | 999 |
| Chr15_388629855 | Chr15 | 388629855 | 10.3605 | 165.259 | 16753 | 999 |
| Chr15_388629862 | Chr15 | 388629862 | 10.4131 | 168.062 | 16838 | 999 |
| Chr15_402998602 | Chr15 | 402998602 | 4.62152 | 26.9978 | 7473 | 999 |
| Chr15_410714361 | Chr15 | 410714361 | 16.9728 | 516.504 | 27445 | 999 |
| Chr15_415764147 | Chr15 | 415764147 | 5.10204 | 31.8776 | 8250 | 999 |
| Chr15_418849616 | Chr15 | 418849616 | 12.4094 | 190.106 | 20066 | 999 |
| Chr15_426169578 | Chr15 | 426169578 | 11.8052 | 249.801 | 19089 | 999 |
| Chr15_427773423 | Chr15 | 427773423 | 18.2888 | 445.42 | 29573 | 999 |
| Chr15_432090946 | Chr15 | 432090946 | 6.60297 | 56.622 | 10677 | 999 |
| Chr15_439429178 | Chr15 | 439429178 | 18.308 | 590.676 | 29604 | 999 |
| Chr15_443816104 | Chr15 | 443816104 | 6.9295 | 50.875 | 11205 | 999 |
| Chr15_450788027 | Chr15 | 450788027 | 7.59493 | 80.7733 | 12281 | 999 |
| Chr15_452858761 | Chr15 | 452858761 | 9.55968 | 147.367 | 15458 | 999 |
| Chr15_452858797 | Chr15 | 452858797 | 9.45949 | 145.247 | 15296 | 999 |
| Chr15_453252569 | Chr15 | 453252569 | 22.4545 | 952.346 | 36309 | 999 |
| Chr15_456695202 | Chr15 | 456695202 | 26.5677 | 1182.85 | 42960 | 999 |
| Chr15_457890816 | Chr15 | 457890816 | 15.2987 | 203.539 | 24738 | 999 |
| Chr15_458358354 | Chr15 | 458358354 | 6.63451 | 37.1863 | 10728 | 999 |
| Chr15_458681762 | Chr15 | 458681762 | 4.49969 | 25.4544 | 7276 | 999 |
| Chr15_458681774 | Chr15 | 458681774 | 4.51268 | 25.5483 | 7297 | 999 |
| Chr15_458681784 | Chr15 | 458681784 | 4.56834 | 25.9608 | 7387 | 999 |
| Chr15_458681822 | Chr15 | 458681822 | 4.67532 | 28.9446 | 7560 | 999 |
| Chr15_459766459 | Chr15 | 459766459 | 6.12987 | 57.4076 | 9912 | 999 |
| Chr15_460439947 | Chr15 | 460439947 | 11.2511 | 98.9394 | 18193 | 999 |
| Chr15_461149548 | Chr15 | 461149548 | 7.90353 | 64.7444 | 12780 | 999 |
| Chr15_465429144 | Chr15 | 465429144 | 5.79963 | 41.966 | 9378 | 999 |
| Chr15_475215926 | Chr15 | 475215926 | 15.7941 | 147.551 | 25539 | 999 |
| Chr15_478140651 | Chr15 | 478140651 | 4.51577 | 28.4603 | 7302 | 999 |
| Chr15_478870282 | Chr15 | 478870282 | 6.27396 | 49.5555 | 10145 | 999 |
| Chr15_481480366 | Chr15 | 481480366 | 7.18058 | 50.1555 | 11611 | 999 |
| Chr15_482004660 | Chr15 | 482004660 | 6.59802 | 56.4188 | 10669 | 999 |
| Chr15_487022107 | Chr15 | 487022107 | 12.6283 | 283.846 | 20420 | 999 |
| Chr15_487022112 | Chr15 | 487022112 | 6.26778 | 63.1182 | 10135 | 999 |
| Chr15_487022115 | Chr15 | 487022115 | 5.28139 | 39.9202 | 8540 | 999 |
| Chr15_487022116 | Chr15 | 487022116 | 5.21954 | 38.9289 | 8440 | 999 |
| Chr15_490873274 | Chr15 | 490873274 | 11.7761 | 259.06 | 19042 | 999 |
| Chr15_490873277 | Chr15 | 490873277 | 11.9598 | 269.66 | 19339 | 999 |
| Chr15_493643310 | Chr15 | 493643310 | 6.17687 | 51.1197 | 9988 | 999 |
| Chr15_493643345 | Chr15 | 493643345 | 6.0872 | 50.9868 | 9843 | 999 |
| Chr15_499145566 | Chr15 | 499145566 | 10.9796 | 198.239 | 17754 | 999 |
| Chr15_499669996 | Chr15 | 499669996 | 27.6456 | 885.028 | 44703 | 999 |
| Chr15_505708324 | Chr15 | 505708324 | 10.3748 | 108.983 | 16776 | 999 |
| Chr15_506013436 | Chr15 | 506013436 | 6.86642 | 50.643 | 11103 | 999 |
| Chr15_506013462 | Chr15 | 506013462 | 6.82931 | 49.6491 | 11043 | 999 |
| Chr15_506013476 | Chr15 | 506013476 | 6.80272 | 49.979 | 11000 | 999 |
| Chr15_506962005 | Chr15 | 506962005 | 7.92022 | 69.0809 | 12807 | 999 |
| Chr15_507689606 | Chr15 | 507689606 | 8.20284 | 85.1507 | 13264 | 999 |
| Chr15_509740265 | Chr15 | 509740265 | 7.51577 | 82.594 | 12153 | 999 |
| Chr15_509939971 | Chr15 | 509939971 | 14.2041 | 282.93 | 22968 | 999 |
| Chr15_512223267 | Chr15 | 512223267 | 5.80767 | 52.4908 | 9391 | 999 |
| Chr15_514887351 | Chr15 | 514887351 | 11.1855 | 156.607 | 18087 | 999 |
| Chr15_514887371 | Chr15 | 514887371 | 11.8163 | 181.773 | 19107 | 999 |
| Chr15_514887385 | Chr15 | 514887385 | 11.7514 | 181.114 | 19002 | 999 |
| Chr15_518228875 | Chr15 | 518228875 | 12.9122 | 304.068 | 20879 | 999 |
| Chr15_521971228 | Chr15 | 521971228 | 7.54174 | 87.111 | 12195 | 999 |
| Chr15_524767527 | Chr15 | 524767527 | 21.1076 | 300.616 | 34131 | 999 |
| Chr15_525388053 | Chr15 | 525388053 | 11.5164 | 200.882 | 18622 | 999 |
| Chr15_534501342 | Chr15 | 534501342 | 11.791 | 163.032 | 19066 | 999 |
| Chr15_536194200 | Chr15 | 536194200 | 7.7248 | 91.4211 | 12491 | 999 |
| Chr15_538682110 | Chr15 | 538682110 | 10.282 | 110.592 | 16626 | 999 |
| Chr15_540439138 | Chr15 | 540439138 | 4.82622 | 30.2687 | 7804 | 999 |
| Chr15_540619940 | Chr15 | 540619940 | 13.5869 | 205.834 | 21970 | 999 |
| Chr15_540779924 | Chr15 | 540779924 | 6.57885 | 65.0583 | 10638 | 999 |
| Chr15_540779930 | Chr15 | 540779930 | 6.5906 | 65.1603 | 10657 | 999 |
| Chr15_563744816 | Chr15 | 563744816 | 15.4014 | 337.705 | 24904 | 999 |
| Chr15_589767878 | Chr15 | 589767878 | 8.7415 | 93.3589 | 14135 | 999 |
| Chr15_589768071 | Chr15 | 589768071 | 14.7767 | 323.421 | 23894 | 999 |
| Chr15_589768085 | Chr15 | 589768085 | 14.731 | 324.522 | 23820 | 999 |
| Chr15_591238203 | Chr15 | 591238203 | 25.4552 | 932.676 | 41161 | 999 |
| Chr15_602281922 | Chr15 | 602281922 | 14.9085 | 394.98 | 24107 | 999 |
| Chr15_602281942 | Chr15 | 602281942 | 14.833 | 389.201 | 23985 | 999 |
| Chr15_606361228 | Chr15 | 606361228 | 19.1905 | 426.664 | 31031 | 999 |
| Chr15_606361233 | Chr15 | 606361233 | 19.4014 | 433.917 | 31372 | 999 |
| Chr15_606361269 | Chr15 | 606361269 | 19.7192 | 445.27 | 31886 | 999 |
| Chr15_606860022 | Chr15 | 606860022 | 27.0891 | 1051.55 | 43803 | 999 |
| Chr15_606860023 | Chr15 | 606860023 | 26.9301 | 1036.84 | 43546 | 999 |
| Chr15_606860061 | Chr15 | 606860061 | 25.1509 | 968.134 | 40669 | 999 |
| Chr15_607652150 | Chr15 | 607652150 | 10.1014 | 114.456 | 16334 | 999 |
| Chr15_607938771 | Chr15 | 607938771 | 5.6376 | 40.9527 | 9116 | 999 |
| Chr15_611405217 | Chr15 | 611405217 | 9.21274 | 147.35 | 14897 | 999 |
| Chr16_10354158 | Chr16 | 10354158 | 7.43414 | 79.8411 | 12021 | 999 |
| Chr16_10354184 | Chr16 | 10354184 | 7.37291 | 78.8565 | 11922 | 999 |
| Chr16_12183406 | Chr16 | 12183406 | 6.60111 | 42.4912 | 10674 | 999 |
| Chr16_13073830 | Chr16 | 13073830 | 6.30365 | 59.8972 | 10193 | 999 |
| Chr16_18496435 | Chr16 | 18496435 | 5.33952 | 35.7801 | 8634 | 999 |
| Chr16_31206780 | Chr16 | 31206780 | 19.107 | 809.145 | 30896 | 999 |
| Chr16_31842624 | Chr16 | 31842624 | 12.6147 | 269.669 | 20398 | 999 |
| Chr16_31865607 | Chr16 | 31865607 | 7.71985 | 72.8454 | 12483 | 999 |
| Chr16_47471843 | Chr16 | 47471843 | 8.80272 | 120.379 | 14234 | 999 |
| Chr16_49871201 | Chr16 | 49871201 | 5.29128 | 37.5927 | 8556 | 999 |
| Chr16_51225353 | Chr16 | 51225353 | 9.31787 | 95.6204 | 15067 | 999 |
| Chr16_51240678 | Chr16 | 51240678 | 4.8188 | 29.5742 | 7792 | 999 |
| Chr16_51491649 | Chr16 | 51491649 | 7.55597 | 82.4562 | 12218 | 999 |
| Chr16_51491687 | Chr16 | 51491687 | 7.45455 | 80.9684 | 12054 | 999 |
| Chr16_51763636 | Chr16 | 51763636 | 15.5857 | 305.529 | 25202 | 999 |
| Chr16_52423801 | Chr16 | 52423801 | 21.603 | 697.789 | 34932 | 999 |
| Chr16_53460762 | Chr16 | 53460762 | 37.9913 | 1071.03 | 61432 | 999 |
| Chr16_57774659 | Chr16 | 57774659 | 12.53 | 154.478 | 20261 | 999 |
| Chr16_57785975 | Chr16 | 57785975 | 18.3327 | 492.723 | 29644 | 999 |
| Chr16_59118108 | Chr16 | 59118108 | 37.8466 | 2734.37 | 61198 | 999 |
| Chr16_60725619 | Chr16 | 60725619 | 26.3908 | 1249.06 | 42674 | 999 |
| Chr16_60727672 | Chr16 | 60727672 | 9.01237 | 96.0667 | 14573 | 999 |
| Chr16_61601076 | Chr16 | 61601076 | 6.35127 | 50.2379 | 10270 | 999 |
| Chr16_61605747 | Chr16 | 61605747 | 6.37662 | 61.1953 | 10311 | 999 |
| Chr16_65032619 | Chr16 | 65032619 | 6.55102 | 65.4109 | 10593 | 999 |
| Chr16_73669588 | Chr16 | 73669588 | 24.5949 | 726.559 | 39770 | 999 |
| Chr16_75232719 | Chr16 | 75232719 | 7.58998 | 105.048 | 12273 | 999 |
| Chr16_76188739 | Chr16 | 76188739 | 12.7124 | 201.912 | 20556 | 999 |
| Chr16_90086280 | Chr16 | 90086280 | 6.93135 | 69.4501 | 11208 | 999 |
| Chr16_90096530 | Chr16 | 90096530 | 6.37662 | 55.4886 | 10311 | 999 |
| Chr16_91285014 | Chr16 | 91285014 | 8.13482 | 68.9707 | 13154 | 999 |
| Chr16_91285017 | Chr16 | 91285017 | 8.06246 | 67.2739 | 13037 | 999 |
| Chr16_92138434 | Chr16 | 92138434 | 10.3853 | 92.2692 | 16793 | 999 |
| Chr16_92138435 | Chr16 | 92138435 | 10.3729 | 91.9951 | 16773 | 999 |
| Chr16_93076859 | Chr16 | 93076859 | 15.5232 | 331.777 | 25101 | 999 |
| Chr16_95907033 | Chr16 | 95907033 | 10.9289 | 134.481 | 17672 | 999 |
| Chr16_123834159 | Chr16 | 123834159 | 8.18738 | 76.1264 | 13239 | 999 |
| Chr16_125675747 | Chr16 | 125675747 | 11.1781 | 104.26 | 18075 | 999 |
| Chr16_132626331 | Chr16 | 132626331 | 7.38219 | 64.0618 | 11937 | 999 |
| Chr16_152594655 | Chr16 | 152594655 | 10.8503 | 111.223 | 17545 | 999 |
| Chr16_168979633 | Chr16 | 168979633 | 4.10761 | 19.976 | 6642 | 999 |
| Chr16_168979664 | Chr16 | 168979664 | 3.96351 | 19.4337 | 6409 | 999 |
| Chr16_168980209 | Chr16 | 168980209 | 9.3556 | 77.4669 | 15128 | 999 |
| Chr16_169019500 | Chr16 | 169019500 | 8.65182 | 90.4882 | 13990 | 999 |
| Chr16_169019504 | Chr16 | 169019504 | 8.63575 | 90.2515 | 13964 | 999 |
| Chr16_187357894 | Chr16 | 187357894 | 25.2294 | 1164.71 | 40796 | 999 |
| Chr16_194326666 | Chr16 | 194326666 | 12.3581 | 253.714 | 19983 | 999 |
| Chr16_200888074 | Chr16 | 200888074 | 9.32839 | 141.593 | 15084 | 999 |
| Chr16_209789747 | Chr16 | 209789747 | 6.96413 | 90.917 | 11261 | 999 |
| Chr16_211807931 | Chr16 | 211807931 | 15.7953 | 440.245 | 25541 | 999 |
| Chr16_221793546 | Chr16 | 221793546 | 4.70872 | 23.4566 | 7614 | 999 |
| Chr16_231387319 | Chr16 | 231387319 | 12.3896 | 236.533 | 20034 | 999 |
| Chr16_231387338 | Chr16 | 231387338 | 11.8924 | 219.082 | 19230 | 999 |
| Chr16_244349484 | Chr16 | 244349484 | 9.18615 | 70.5959 | 14854 | 999 |
| Chr16_250448416 | Chr16 | 250448416 | 8.99443 | 128.903 | 14544 | 999 |
| Chr16_250448439 | Chr16 | 250448439 | 9.42548 | 143.012 | 15241 | 999 |
| Chr16_250589266 | Chr16 | 250589266 | 6.08163 | 49.5317 | 9834 | 999 |
| Chr16_263001852 | Chr16 | 263001852 | 11.2208 | 153.584 | 18144 | 999 |
| Chr16_263002123 | Chr16 | 263002123 | 11.8058 | 131.445 | 19090 | 999 |
| Chr16_263002131 | Chr16 | 263002131 | 11.8627 | 131.974 | 19182 | 999 |
| Chr16_263002159 | Chr16 | 263002159 | 11.9165 | 133.474 | 19269 | 999 |
| Chr16_266495035 | Chr16 | 266495035 | 22.914 | 675.462 | 37052 | 999 |
| Chr16_269952338 | Chr16 | 269952338 | 11.4075 | 124.471 | 18446 | 999 |
| Chr16_270632318 | Chr16 | 270632318 | 6.22387 | 50.6937 | 10064 | 999 |
| Chr16_270668800 | Chr16 | 270668800 | 7.95238 | 74.8969 | 12859 | 999 |
| Chr16_270668834 | Chr16 | 270668834 | 8.10328 | 77.7201 | 13103 | 999 |
| Chr16_280752428 | Chr16 | 280752428 | 10.6475 | 135.831 | 17217 | 999 |
| Chr16_284865296 | Chr16 | 284865296 | 7.71119 | 68.5162 | 12469 | 999 |
| Chr16_296781475 | Chr16 | 296781475 | 19.59 | 483.001 | 31677 | 999 |
| Chr16_296781515 | Chr16 | 296781515 | 22.389 | 631.4 | 36203 | 999 |
| Chr16_307761673 | Chr16 | 307761673 | 30.9474 | 782.681 | 50042 | 999 |
| Chr16_312692615 | Chr16 | 312692615 | 7.05751 | 57.2114 | 11412 | 999 |
| Chr16_312692616 | Chr16 | 312692616 | 7.0538 | 56.9556 | 11406 | 999 |
| Chr16_320552165 | Chr16 | 320552165 | 7.7953 | 84.0602 | 12605 | 999 |
| Chr16_320552170 | Chr16 | 320552170 | 7.77365 | 83.1703 | 12570 | 999 |
| Chr16_327943710 | Chr16 | 327943710 | 4.21769 | 21.7533 | 6820 | 999 |
| Chr16_334440368 | Chr16 | 334440368 | 9.71985 | 108.344 | 15717 | 999 |
| Chr16_336629845 | Chr16 | 336629845 | 9.03463 | 78.1448 | 14609 | 999 |
| Chr16_340188600 | Chr16 | 340188600 | 14.1206 | 296.786 | 22833 | 999 |
| Chr16_346120540 | Chr16 | 346120540 | 24.6085 | 1179.29 | 39792 | 999 |
| Chr16_352718841 | Chr16 | 352718841 | 9.51763 | 71.8711 | 15390 | 999 |
| Chr16_354370625 | Chr16 | 354370625 | 7.80767 | 78.1282 | 12625 | 999 |
| Chr16_355544491 | Chr16 | 355544491 | 14.9722 | 317.912 | 24210 | 999 |
| Chr16_356943138 | Chr16 | 356943138 | 11.0557 | 211.272 | 17877 | 999 |
| Chr16_356943139 | Chr16 | 356943139 | 11.0124 | 209.216 | 17807 | 999 |
| Chr16_357370264 | Chr16 | 357370264 | 4.28262 | 23.0086 | 6925 | 999 |
| Chr16_360808262 | Chr16 | 360808262 | 5.2115 | 44.8104 | 8427 | 999 |
| Chr16_364953581 | Chr16 | 364953581 | 8.28942 | 103.714 | 13404 | 999 |
| Chr16_364953603 | Chr16 | 364953603 | 8.32282 | 102.513 | 13458 | 999 |
| Chr16_364953625 | Chr16 | 364953625 | 8.40631 | 104.339 | 13593 | 999 |
| Chr16_371835395 | Chr16 | 371835395 | 8.35374 | 78.8921 | 13508 | 999 |
| Chr16_371835418 | Chr16 | 371835418 | 8.46506 | 81.3764 | 13688 | 999 |
| Chr16_371835431 | Chr16 | 371835431 | 8.41002 | 80.1084 | 13599 | 999 |
| Chr16_377637132 | Chr16 | 377637132 | 11.0668 | 190.029 | 17895 | 999 |
| Chr16_386030919 | Chr16 | 386030919 | 14.6543 | 134.99 | 23696 | 999 |
| Chr16_386689837 | Chr16 | 386689837 | 14.1206 | 178.416 | 22833 | 999 |
| Chr16_388899714 | Chr16 | 388899714 | 9.37229 | 77.0482 | 15155 | 999 |
| Chr16_388935948 | Chr16 | 388935948 | 23.3321 | 816.512 | 37728 | 999 |
| Chr16_388935952 | Chr16 | 388935952 | 23.261 | 811.119 | 37613 | 999 |
| Chr16_388935954 | Chr16 | 388935954 | 19.744 | 569.738 | 31926 | 999 |
| Chr16_388936018 | Chr16 | 388936018 | 5.29623 | 30.6876 | 8564 | 999 |
| Chr16_388936039 | Chr16 | 388936039 | 5.20161 | 30.317 | 8411 | 999 |
| Chr16_394511623 | Chr16 | 394511623 | 6.34756 | 46.0103 | 10264 | 999 |
| Chr16_394511631 | Chr16 | 394511631 | 6.35931 | 46.1425 | 10283 | 999 |
| Chr16_394511636 | Chr16 | 394511636 | 6.36673 | 46.3425 | 10295 | 999 |
| Chr16_395073387 | Chr16 | 395073387 | 15.7817 | 347.137 | 25519 | 999 |
| Chr16_395785826 | Chr16 | 395785826 | 8.57019 | 91.744 | 13858 | 999 |
| Chr16_399008245 | Chr16 | 399008245 | 9.33952 | 172.811 | 15102 | 999 |
| Chr16_399008259 | Chr16 | 399008259 | 9.52072 | 178.99 | 15395 | 999 |
| Chr16_399085294 | Chr16 | 399085294 | 9.68955 | 190.473 | 15668 | 999 |
| Chr16_399244042 | Chr16 | 399244042 | 7.29437 | 64.7276 | 11795 | 999 |
| Chr17_8646432 | Chr17 | 8646432 | 8.56401 | 78.7225 | 13848 | 999 |
| Chr17_10742923 | Chr17 | 10742923 | 9.96042 | 148.216 | 16106 | 999 |
| Chr17_10742924 | Chr17 | 10742924 | 9.97835 | 148.741 | 16135 | 999 |
| Chr17_10910087 | Chr17 | 10910087 | 10.517 | 182.057 | 17006 | 999 |
| Chr17_10910152 | Chr17 | 10910152 | 9.75943 | 152.55 | 15781 | 999 |
| Chr17_11178657 | Chr17 | 11178657 | 8.53741 | 85.0507 | 13805 | 999 |
| Chr17_11178659 | Chr17 | 11178659 | 8.52566 | 84.6653 | 13786 | 999 |
| Chr17_13535956 | Chr17 | 13535956 | 15.927 | 197.091 | 25754 | 999 |
| Chr17_14065131 | Chr17 | 14065131 | 7.06432 | 75.481 | 11423 | 999 |
| Chr17_14065132 | Chr17 | 14065132 | 7.07174 | 75.9305 | 11435 | 999 |
| Chr17_14680025 | Chr17 | 14680025 | 12.9357 | 166.053 | 20917 | 999 |
| Chr17_14680066 | Chr17 | 14680066 | 12.047 | 153.244 | 19480 | 999 |
| Chr17_18337303 | Chr17 | 18337303 | 9.41187 | 85.5902 | 15219 | 999 |
| Chr17_18656965 | Chr17 | 18656965 | 7.81262 | 107.712 | 12633 | 999 |
| Chr17_19404341 | Chr17 | 19404341 | 17.4947 | 580.066 | 28289 | 999 |
| Chr17_19404363 | Chr17 | 19404363 | 17.7347 | 590.054 | 28677 | 999 |
| Chr17_19404385 | Chr17 | 19404385 | 17.7879 | 592.233 | 28763 | 999 |
| Chr17_19404389 | Chr17 | 19404389 | 17.8479 | 599.415 | 28860 | 999 |
| Chr17_19424964 | Chr17 | 19424964 | 9.65182 | 90.7407 | 15607 | 999 |
| Chr17_21243698 | Chr17 | 21243698 | 10.4001 | 173.029 | 16817 | 999 |
| Chr17_25479345 | Chr17 | 25479345 | 8.01113 | 75.6447 | 12954 | 999 |
| Chr17_25479353 | Chr17 | 25479353 | 8.34941 | 85.4824 | 13501 | 999 |
| Chr17_25479367 | Chr17 | 25479367 | 8.26592 | 85.6953 | 13366 | 999 |
| Chr17_25479370 | Chr17 | 25479370 | 8.23995 | 84.628 | 13324 | 999 |
| Chr17_25479372 | Chr17 | 25479372 | 8.17069 | 83.482 | 13212 | 999 |
| Chr17_25479377 | Chr17 | 25479377 | 8.20161 | 84.7242 | 13262 | 999 |
| Chr17_25479380 | Chr17 | 25479380 | 8.2449 | 85.9944 | 13332 | 999 |
| Chr17_25479389 | Chr17 | 25479389 | 8.13544 | 83.5813 | 13155 | 999 |
| Chr17_25479397 | Chr17 | 25479397 | 7.96599 | 80.695 | 12881 | 999 |
| Chr17_25479405 | Chr17 | 25479405 | 7.93321 | 81.2975 | 12828 | 999 |
| Chr17_27063529 | Chr17 | 27063529 | 7.62214 | 71.5991 | 12325 | 999 |
| Chr17_29387790 | Chr17 | 29387790 | 10.8132 | 190.992 | 17485 | 999 |
| Chr17_30112537 | Chr17 | 30112537 | 10.8472 | 174.683 | 17540 | 999 |
| Chr17_30209443 | Chr17 | 30209443 | 15.9672 | 310.804 | 25819 | 999 |
| Chr17_30209457 | Chr17 | 30209457 | 16.0074 | 310.469 | 25884 | 999 |
| Chr17_30209493 | Chr17 | 30209493 | 13.4292 | 225.609 | 21715 | 999 |
| Chr17_30209507 | Chr17 | 30209507 | 13.397 | 224.615 | 21663 | 999 |
| Chr17_30363556 | Chr17 | 30363556 | 11.2542 | 211.304 | 18198 | 999 |
| Chr17_33554473 | Chr17 | 33554473 | 5.44403 | 30.4834 | 8803 | 999 |
| Chr17_41652287 | Chr17 | 41652287 | 8.22449 | 135.105 | 13299 | 999 |
| Chr17_43148415 | Chr17 | 43148415 | 15.1707 | 278.039 | 24531 | 999 |
| Chr17_43528098 | Chr17 | 43528098 | 14.3166 | 281.506 | 23150 | 999 |
| Chr17_43528103 | Chr17 | 43528103 | 14.0482 | 267.711 | 22716 | 999 |
| Chr17_43528114 | Chr17 | 43528114 | 14.4552 | 287.038 | 23374 | 999 |
| Chr17_43528121 | Chr17 | 43528121 | 14.2659 | 273.809 | 23068 | 999 |
| Chr17_43571781 | Chr17 | 43571781 | 6.96475 | 40.1132 | 11262 | 999 |
| Chr17_45550764 | Chr17 | 45550764 | 6.55844 | 36.7381 | 10605 | 999 |
| Chr17_46698699 | Chr17 | 46698699 | 13.3605 | 180.757 | 21604 | 999 |
| Chr17_46855298 | Chr17 | 46855298 | 5.65739 | 34.5744 | 9148 | 999 |
| Chr17_47017253 | Chr17 | 47017253 | 4.49536 | 24.109 | 7269 | 999 |
| Chr17_47017263 | Chr17 | 47017263 | 4.48114 | 23.7944 | 7246 | 999 |
| Chr17_47186687 | Chr17 | 47186687 | 6.58442 | 58.0413 | 10647 | 999 |
| Chr17_47720429 | Chr17 | 47720429 | 6.62028 | 46.732 | 10705 | 999 |
| Chr17_47720482 | Chr17 | 47720482 | 6.3791 | 44.9558 | 10315 | 999 |
| Chr17_47933414 | Chr17 | 47933414 | 8.59988 | 91.8342 | 13906 | 999 |
| Chr17_54254033 | Chr17 | 54254033 | 8.10575 | 71.1231 | 13107 | 999 |
| Chr17_54254056 | Chr17 | 54254056 | 8.19481 | 72.0555 | 13251 | 999 |
| Chr17_58017726 | Chr17 | 58017726 | 15.5683 | 485.53 | 25174 | 999 |
| Chr17_62223159 | Chr17 | 62223159 | 7.35065 | 66.2377 | 11886 | 999 |
| Chr17_65241599 | Chr17 | 65241599 | 6.89549 | 61.0281 | 11150 | 999 |
| Chr17_79845235 | Chr17 | 79845235 | 7.67594 | 63.3256 | 12412 | 999 |
| Chr17_79845253 | Chr17 | 79845253 | 7.67904 | 63.5374 | 12417 | 999 |
| Chr17_80562247 | Chr17 | 80562247 | 10.3278 | 139.734 | 16700 | 999 |
| Chr17_80562251 | Chr17 | 80562251 | 10.3173 | 138.69 | 16683 | 999 |
| Chr17_81683948 | Chr17 | 81683948 | 12.9728 | 186.769 | 20977 | 999 |
| Chr17_81683951 | Chr17 | 81683951 | 12.9017 | 184.148 | 20862 | 999 |
| Chr17_81683964 | Chr17 | 81683964 | 12.9456 | 184.387 | 20933 | 999 |
| Chr17_96387798 | Chr17 | 96387798 | 5.79654 | 46.8602 | 9373 | 999 |
| Chr17_131018659 | Chr17 | 131018659 | 5.35683 | 47.6987 | 8662 | 999 |
| Chr17_131018680 | Chr17 | 131018680 | 5.50835 | 49.6387 | 8907 | 999 |
| Chr17_200299872 | Chr17 | 200299872 | 12.2752 | 142.243 | 19849 | 999 |
| Chr17_206771472 | Chr17 | 206771472 | 8.32777 | 69.7031 | 13466 | 999 |
| Chr17_213557598 | Chr17 | 213557598 | 7.54855 | 59.2602 | 12206 | 999 |
| Chr17_214166031 | Chr17 | 214166031 | 21.8027 | 756.231 | 35255 | 999 |
| Chr17_217236328 | Chr17 | 217236328 | 6.30303 | 34.048 | 10192 | 999 |
| Chr17_241561354 | Chr17 | 241561354 | 9.59617 | 115.547 | 15517 | 999 |
| Chr17_255864920 | Chr17 | 255864920 | 22.3173 | 354.978 | 36087 | 999 |
| Chr17_255864940 | Chr17 | 255864940 | 16.9926 | 197.984 | 27477 | 999 |
| Chr17_255864960 | Chr17 | 255864960 | 16.9116 | 196.979 | 27346 | 999 |
| Chr17_259952955 | Chr17 | 259952955 | 6.671 | 72.6504 | 10787 | 999 |
| Chr17_264261264 | Chr17 | 264261264 | 7.02226 | 47.9327 | 11355 | 999 |
| Chr17_265332347 | Chr17 | 265332347 | 31.0934 | 1443.82 | 50278 | 999 |
| Chr17_271396013 | Chr17 | 271396013 | 19.0618 | 533.589 | 30823 | 999 |
| Chr17_271513694 | Chr17 | 271513694 | 10.8417 | 175.114 | 17531 | 999 |
| Chr17_273226718 | Chr17 | 273226718 | 13.5813 | 182.425 | 21961 | 999 |
| Chr17_273309330 | Chr17 | 273309330 | 10.12 | 97.4534 | 16364 | 999 |
| Chr17_279198557 | Chr17 | 279198557 | 7.39208 | 72.465 | 11953 | 999 |
| Chr17_282706126 | Chr17 | 282706126 | 14.1008 | 342.044 | 22801 | 999 |
| Chr17_285315129 | Chr17 | 285315129 | 6.48732 | 61.0124 | 10490 | 999 |
| Chr17_295258499 | Chr17 | 295258499 | 7.71243 | 68.2075 | 12471 | 999 |
| Chr17_295258525 | Chr17 | 295258525 | 7.71861 | 68.2593 | 12481 | 999 |
| Chr17_295535735 | Chr17 | 295535735 | 13.3599 | 322.978 | 21603 | 999 |
| Chr17_295535739 | Chr17 | 295535739 | 13.4873 | 329.193 | 21809 | 999 |
| Chr17_297941441 | Chr17 | 297941441 | 14.9629 | 337.427 | 24195 | 999 |
| Chr17_300103726 | Chr17 | 300103726 | 7.59926 | 83.546 | 12288 | 999 |
| Chr17_303808921 | Chr17 | 303808921 | 6.3624 | 56.9206 | 10288 | 999 |
| Chr17_304018231 | Chr17 | 304018231 | 32.7706 | 1989.23 | 52990 | 999 |
| Chr17_304173832 | Chr17 | 304173832 | 6.48361 | 45.1496 | 10484 | 999 |
| Chr17_304173841 | Chr17 | 304173841 | 6.47372 | 44.8485 | 10468 | 999 |
| Chr17_304675487 | Chr17 | 304675487 | 10.9524 | 187.724 | 17710 | 999 |
| Chr17_304902691 | Chr17 | 304902691 | 19.4694 | 376.884 | 31482 | 999 |
| Chr17_304902743 | Chr17 | 304902743 | 18.0062 | 350.798 | 29116 | 999 |
| Chr17_314542423 | Chr17 | 314542423 | 9.21645 | 105.449 | 14903 | 999 |
| Chr17_314542429 | Chr17 | 314542429 | 9.25974 | 106.884 | 14973 | 999 |
| Chr17_314542445 | Chr17 | 314542445 | 9.24181 | 106.071 | 14944 | 999 |
| Chr17_314542451 | Chr17 | 314542451 | 9.4731 | 109.258 | 15318 | 999 |
| Chr17_314542453 | Chr17 | 314542453 | 9.31293 | 104.329 | 15059 | 999 |
| Chr17_316186528 | Chr17 | 316186528 | 6.671 | 59.3867 | 10787 | 999 |
| Chr17_316632524 | Chr17 | 316632524 | 11.0489 | 179.138 | 17866 | 999 |
| Chr17_316907984 | Chr17 | 316907984 | 7.5603 | 51.1549 | 12225 | 999 |
| Chr17_319781231 | Chr17 | 319781231 | 6.04947 | 52.6337 | 9782 | 999 |
| Chr17_319781264 | Chr17 | 319781264 | 5.97588 | 50.3552 | 9663 | 999 |
| Chr17_320151726 | Chr17 | 320151726 | 16.3816 | 417.899 | 26489 | 999 |
| Chr17_320151779 | Chr17 | 320151779 | 16.7681 | 431.281 | 27114 | 999 |
| Chr17_324778448 | Chr17 | 324778448 | 7.00371 | 66.1423 | 11325 | 999 |
| Chr17_329983264 | Chr17 | 329983264 | 30.7532 | 1041.31 | 49728 | 999 |
| Chr17_335013495 | Chr17 | 335013495 | 17.4688 | 355.534 | 28247 | 999 |
| Chr18_6537418 | Chr18 | 6537418 | 13.3525 | 246.629 | 21591 | 999 |
| Chr18_6828915 | Chr18 | 6828915 | 13.2468 | 287.655 | 21420 | 999 |
| Chr18_6828924 | Chr18 | 6828924 | 13.2517 | 288.807 | 21428 | 999 |
| Chr18_10040085 | Chr18 | 10040085 | 8.03401 | 88.0799 | 12991 | 999 |
| Chr18_17030492 | Chr18 | 17030492 | 9.28262 | 137.372 | 15010 | 999 |
| Chr18_18028017 | Chr18 | 18028017 | 9.38652 | 129.665 | 15178 | 999 |
| Chr18_19253772 | Chr18 | 19253772 | 22.3649 | 565.242 | 36164 | 999 |
| Chr18_19412579 | Chr18 | 19412579 | 11.2251 | 173.46 | 18151 | 999 |
| Chr18_19412591 | Chr18 | 19412591 | 11.1503 | 171.896 | 18030 | 999 |
| Chr18_19412626 | Chr18 | 19412626 | 11.0167 | 170.985 | 17814 | 999 |
| Chr18_19444364 | Chr18 | 19444364 | 11.9505 | 167.858 | 19324 | 999 |
| Chr18_19575918 | Chr18 | 19575918 | 14.6667 | 331.92 | 23716 | 999 |
| Chr18_19575941 | Chr18 | 19575941 | 14.8479 | 344.38 | 24009 | 999 |
| Chr18_19575980 | Chr18 | 19575980 | 7.66914 | 78.7686 | 12401 | 999 |
| Chr18_20045321 | Chr18 | 20045321 | 8.98887 | 95.5085 | 14535 | 999 |
| Chr18_20045389 | Chr18 | 20045389 | 11.2554 | 199.687 | 18200 | 999 |
| Chr18_20045490 | Chr18 | 20045490 | 5.71614 | 41.6898 | 9243 | 999 |
| Chr18_21052622 | Chr18 | 21052622 | 13.7483 | 310.06 | 22231 | 999 |
| Chr18_21052627 | Chr18 | 21052627 | 13.859 | 315.663 | 22410 | 999 |
| Chr18_21052628 | Chr18 | 21052628 | 13.7582 | 310.091 | 22247 | 999 |
| Chr18_21052632 | Chr18 | 21052632 | 13.7032 | 307.411 | 22158 | 999 |
| Chr18_21052633 | Chr18 | 21052633 | 13.6852 | 308.835 | 22129 | 999 |
| Chr18_30692145 | Chr18 | 30692145 | 6.92084 | 67.5432 | 11191 | 999 |
| Chr18_33540624 | Chr18 | 33540624 | 11.7205 | 132.133 | 18952 | 999 |
| Chr18_33540628 | Chr18 | 33540628 | 11.6889 | 130.077 | 18901 | 999 |
| Chr18_33540641 | Chr18 | 33540641 | 11.4898 | 123.953 | 18579 | 999 |
| Chr18_34298305 | Chr18 | 34298305 | 10.7941 | 193.722 | 17454 | 999 |
| Chr18_34298309 | Chr18 | 34298309 | 11.3488 | 204.706 | 18351 | 999 |
| Chr18_34298324 | Chr18 | 34298324 | 11.3284 | 205.512 | 18318 | 999 |
| Chr18_34885160 | Chr18 | 34885160 | 9.2616 | 109.367 | 14976 | 999 |
| Chr18_35876661 | Chr18 | 35876661 | 7.41682 | 86.8695 | 11993 | 999 |
| Chr18_35876707 | Chr18 | 35876707 | 8.3055 | 114.967 | 13430 | 999 |
| Chr18_35876710 | Chr18 | 35876710 | 8.29808 | 114.978 | 13418 | 999 |
| Chr18_36724510 | Chr18 | 36724510 | 11.7069 | 184.608 | 18930 | 999 |
| Chr18_41360647 | Chr18 | 41360647 | 10.5009 | 162.541 | 16980 | 999 |
| Chr18_43279348 | Chr18 | 43279348 | 11.6289 | 143.443 | 18804 | 999 |
| Chr18_44104789 | Chr18 | 44104789 | 15.7328 | 411.029 | 25440 | 999 |
| Chr18_44439589 | Chr18 | 44439589 | 11.6772 | 208.489 | 18882 | 999 |
| Chr18_48253637 | Chr18 | 48253637 | 5.10328 | 36.7325 | 8252 | 999 |
| Chr18_50878498 | Chr18 | 50878498 | 5.85714 | 35.9889 | 9471 | 999 |
| Chr18_50972176 | Chr18 | 50972176 | 13.8905 | 373.152 | 22461 | 999 |
| Chr18_50972217 | Chr18 | 50972217 | 12.1039 | 259.226 | 19572 | 999 |
| Chr18_51327239 | Chr18 | 51327239 | 6.96599 | 53.174 | 11264 | 999 |
| Chr18_52297736 | Chr18 | 52297736 | 7.66852 | 85.1586 | 12400 | 999 |
| Chr18_58298079 | Chr18 | 58298079 | 25.6685 | 1256.54 | 41506 | 999 |
| Chr18_58330818 | Chr18 | 58330818 | 7.54978 | 106.694 | 12208 | 999 |
| Chr18_58330826 | Chr18 | 58330826 | 8.05009 | 124.956 | 13017 | 999 |
| Chr18_58330862 | Chr18 | 58330862 | 8.18615 | 133.053 | 13237 | 999 |
| Chr18_63398463 | Chr18 | 63398463 | 11.1806 | 145.372 | 18079 | 999 |
| Chr18_63398485 | Chr18 | 63398485 | 11.1571 | 145.203 | 18041 | 999 |
| Chr18_64304357 | Chr18 | 64304357 | 9.30303 | 132.6 | 15043 | 999 |
| Chr18_64304389 | Chr18 | 64304389 | 9.20037 | 130.795 | 14877 | 999 |
| Chr18_69798217 | Chr18 | 69798217 | 15.5764 | 354.767 | 25187 | 999 |
| Chr18_69798220 | Chr18 | 69798220 | 15.7211 | 362.373 | 25421 | 999 |
| Chr18_75322511 | Chr18 | 75322511 | 8.38157 | 54.8586 | 13553 | 999 |
| Chr18_75576716 | Chr18 | 75576716 | 8.67285 | 118.416 | 14024 | 999 |
| Chr18_76961188 | Chr18 | 76961188 | 14.4576 | 406.287 | 23378 | 999 |
| Chr18_89698168 | Chr18 | 89698168 | 14.1639 | 164.635 | 22903 | 999 |
| Chr18_99548176 | Chr18 | 99548176 | 8.07483 | 53.719 | 13057 | 999 |
| Chr18_99728024 | Chr18 | 99728024 | 19.9802 | 618.122 | 32308 | 999 |
| Chr18_101809842 | Chr18 | 101809842 | 20.047 | 648.662 | 32416 | 999 |
| Chr18_111491335 | Chr18 | 111491335 | 7.10946 | 67.3946 | 11496 | 999 |
| Chr18_133903651 | Chr18 | 133903651 | 6.37972 | 51.175 | 10316 | 999 |
| Chr18_133903660 | Chr18 | 133903660 | 6.32406 | 50.0583 | 10226 | 999 |
| Chr18_157100079 | Chr18 | 157100079 | 5.31911 | 33.3461 | 8601 | 999 |
| Chr18_170459413 | Chr18 | 170459413 | 19.757 | 714.928 | 31947 | 999 |
| Chr18_183704500 | Chr18 | 183704500 | 4.47062 | 27.3495 | 7229 | 999 |
| Chr18_183852245 | Chr18 | 183852245 | 14.9406 | 199.573 | 24159 | 999 |
| Chr18_221889090 | Chr18 | 221889090 | 8.71119 | 77.7922 | 14086 | 999 |
| Chr18_247659152 | Chr18 | 247659152 | 7.46691 | 69.3283 | 12074 | 999 |
| Chr18_250969815 | Chr18 | 250969815 | 5.62276 | 46.7858 | 9092 | 999 |
| Chr18_253256835 | Chr18 | 253256835 | 9.43228 | 93.7901 | 15252 | 999 |
| Chr18_253256845 | Chr18 | 253256845 | 9.41126 | 92.4341 | 15218 | 999 |
| Chr18_253256867 | Chr18 | 253256867 | 9.43105 | 93.2838 | 15250 | 999 |
| Chr18_253256872 | Chr18 | 253256872 | 9.38528 | 92.2469 | 15176 | 999 |
| Chr18_267021512 | Chr18 | 267021512 | 12.0823 | 253.949 | 19537 | 999 |
| Chr18_278531605 | Chr18 | 278531605 | 18.1633 | 575.56 | 29370 | 999 |
| Chr18_282150836 | Chr18 | 282150836 | 7.41064 | 71.1803 | 11983 | 999 |
| Chr18_282150838 | Chr18 | 282150838 | 7.36116 | 69.7915 | 11903 | 999 |
| Chr18_285426927 | Chr18 | 285426927 | 19.5764 | 426.981 | 31655 | 999 |
| Chr18_285426942 | Chr18 | 285426942 | 19.7805 | 440.624 | 31985 | 999 |
| Chr18_285426951 | Chr18 | 285426951 | 19.7421 | 437.725 | 31923 | 999 |
| Chr18_285426954 | Chr18 | 285426954 | 19.6302 | 431.978 | 31742 | 999 |
| Chr18_296087679 | Chr18 | 296087679 | 8.5974 | 82.0662 | 13902 | 999 |
| Chr18_302814381 | Chr18 | 302814381 | 14.4378 | 361.181 | 23346 | 999 |
| Chr18_302814388 | Chr18 | 302814388 | 13.4409 | 299.905 | 21734 | 999 |
| Chr18_303391850 | Chr18 | 303391850 | 11.7959 | 207.582 | 19074 | 999 |
| Chr18_303391865 | Chr18 | 303391865 | 11.6902 | 206.197 | 18903 | 999 |
| Chr18_305609202 | Chr18 | 305609202 | 6.32158 | 39.2195 | 10222 | 999 |
| Chr18_315438196 | Chr18 | 315438196 | 7.15152 | 47.5754 | 11564 | 999 |
| Chr18_360765903 | Chr18 | 360765903 | 18.8695 | 394.3 | 30512 | 999 |
| Chr18_360765925 | Chr18 | 360765925 | 19.4416 | 425.896 | 31437 | 999 |
| Chr18_373489798 | Chr18 | 373489798 | 10.8361 | 121.292 | 17522 | 999 |
| Chr18_379473328 | Chr18 | 379473328 | 9.78479 | 104.945 | 15822 | 999 |
| Chr18_390622413 | Chr18 | 390622413 | 20.5102 | 488.651 | 33165 | 999 |
| Chr18_390622416 | Chr18 | 390622416 | 20.5127 | 489.158 | 33169 | 999 |
| Chr18_404451787 | Chr18 | 404451787 | 10.1039 | 161.834 | 16338 | 999 |
| Chr18_405327406 | Chr18 | 405327406 | 17.188 | 259.351 | 27793 | 999 |
| Chr18_407367603 | Chr18 | 407367603 | 5.27953 | 35.1433 | 8537 | 999 |
| Chr18_407384852 | Chr18 | 407384852 | 3.6141 | 14.9587 | 5844 | 999 |
| Chr18_408114542 | Chr18 | 408114542 | 11.2839 | 113.613 | 18246 | 999 |
| Chr18_408909837 | Chr18 | 408909837 | 8.54545 | 124.674 | 13818 | 999 |
| Chr18_408909840 | Chr18 | 408909840 | 8.54607 | 124.275 | 13819 | 999 |
| Chr18_417402616 | Chr18 | 417402616 | 8.92764 | 117.898 | 14436 | 999 |
| Chr18_423620376 | Chr18 | 423620376 | 12.1484 | 164.081 | 19644 | 999 |
| Chr18_427456631 | Chr18 | 427456631 | 7.18429 | 59.4413 | 11617 | 999 |
| Chr18_429999797 | Chr18 | 429999797 | 12.7372 | 200.454 | 20596 | 999 |
| Chr18_431599578 | Chr18 | 431599578 | 9.15894 | 103.215 | 14810 | 999 |
| Chr18_431713480 | Chr18 | 431713480 | 10.3271 | 136.142 | 16699 | 999 |
| Chr18_431713504 | Chr18 | 431713504 | 10.0754 | 127.845 | 16292 | 999 |
| Chr18_438792035 | Chr18 | 438792035 | 9.11441 | 126.889 | 14738 | 999 |
| Chr18_438792068 | Chr18 | 438792068 | 9.57699 | 142.648 | 15486 | 999 |
| Chr18_442482704 | Chr18 | 442482704 | 4.624 | 28.6221 | 7477 | 999 |
| Chr18_442482706 | Chr18 | 442482706 | 4.70748 | 29.7046 | 7612 | 999 |
| Chr18_442482737 | Chr18 | 442482737 | 4.72109 | 29.8646 | 7634 | 999 |
| Chr18_442482747 | Chr18 | 442482747 | 4.75634 | 30.3515 | 7691 | 999 |
| Chr18_442482754 | Chr18 | 442482754 | 4.75201 | 30.1718 | 7684 | 999 |
| Chr18_457907614 | Chr18 | 457907614 | 14.3408 | 277.126 | 23189 | 999 |
| Chr18_463910496 | Chr18 | 463910496 | 4.49969 | 19.343 | 7276 | 999 |
| Chr18_463910534 | Chr18 | 463910534 | 4.88992 | 21.1995 | 7907 | 999 |
| Chr18_463910540 | Chr18 | 463910540 | 4.87384 | 21.0917 | 7881 | 999 |
| Chr18_470423250 | Chr18 | 470423250 | 8.30241 | 97.2804 | 13425 | 999 |
| Chr18_473218145 | Chr18 | 473218145 | 14.0823 | 408.875 | 22771 | 999 |
| Chr18_475719714 | Chr18 | 475719714 | 15.9598 | 499.414 | 25807 | 999 |
| Chr18_485729897 | Chr18 | 485729897 | 7.00804 | 60.9213 | 11332 | 999 |
| Chr18_488726372 | Chr18 | 488726372 | 9.60606 | 224.925 | 15533 | 999 |
| Chr18_489830502 | Chr18 | 489830502 | 7.14409 | 60.4774 | 11552 | 999 |
| Chr18_492032392 | Chr18 | 492032392 | 9.69202 | 141.364 | 15672 | 999 |
| Chr18_493241151 | Chr18 | 493241151 | 9.01793 | 68.5114 | 14582 | 999 |
| Chr18_499438433 | Chr18 | 499438433 | 7.24366 | 55.6597 | 11713 | 999 |
| Chr18_499449590 | Chr18 | 499449590 | 21.9746 | 670.208 | 35533 | 999 |
| Chr18_501454051 | Chr18 | 501454051 | 10.4923 | 95.5211 | 16966 | 999 |
| Chr18_502665014 | Chr18 | 502665014 | 7.69079 | 104.943 | 12436 | 999 |
| Chr18_504576121 | Chr18 | 504576121 | 5.24923 | 37.3172 | 8488 | 999 |
| Chr18_504576146 | Chr18 | 504576146 | 5.07978 | 36.8705 | 8214 | 999 |
| Chr18_517967561 | Chr18 | 517967561 | 6.85034 | 53.2598 | 11077 | 999 |
| Chr18_518854721 | Chr18 | 518854721 | 6.01855 | 49.4365 | 9732 | 999 |
| Chr18_531368669 | Chr18 | 531368669 | 6.81323 | 59.1668 | 11017 | 999 |
| Chr18_533305980 | Chr18 | 533305980 | 9.13853 | 126.022 | 14777 | 999 |
| Chr18_539437672 | Chr18 | 539437672 | 8.61039 | 114.713 | 13923 | 999 |
| Chr18_539437676 | Chr18 | 539437676 | 9.56648 | 144.782 | 15469 | 999 |
| Chr18_540735168 | Chr18 | 540735168 | 5.55906 | 52.8283 | 8989 | 999 |
| Chr18_540735175 | Chr18 | 540735175 | 5.64935 | 54.7724 | 9135 | 999 |
| Chr18_540735188 | Chr18 | 540735188 | 5.59988 | 52.5508 | 9055 | 999 |
| Chr18_540735198 | Chr18 | 540735198 | 5.67842 | 54.3879 | 9182 | 999 |
| Chr18_540769402 | Chr18 | 540769402 | 5.6209 | 45.2677 | 9089 | 999 |
| Chr18_551073798 | Chr18 | 551073798 | 19.8565 | 439.744 | 32108 | 999 |
| Chr18_554021022 | Chr18 | 554021022 | 5.87941 | 38.1173 | 9507 | 999 |
| Chr18_554021029 | Chr18 | 554021029 | 5.89177 | 38.2896 | 9527 | 999 |
| Chr19_14788033 | Chr19 | 14788033 | 20.8782 | 507.863 | 33760 | 999 |
| Chr19_24523974 | Chr19 | 24523974 | 8.45949 | 114.433 | 13679 | 999 |
| Chr19_24523999 | Chr19 | 24523999 | 8.67718 | 117.12 | 14031 | 999 |
| Chr19_24524002 | Chr19 | 24524002 | 8.62276 | 116.087 | 13943 | 999 |
| Chr19_26371266 | Chr19 | 26371266 | 14.0513 | 236.8 | 22721 | 999 |
| Chr19_26371332 | Chr19 | 26371332 | 11.3884 | 126.777 | 18415 | 999 |
| Chr19_29503024 | Chr19 | 29503024 | 5.64131 | 29.5136 | 9122 | 999 |
| Chr19_29877455 | Chr19 | 29877455 | 11.0581 | 136.191 | 17881 | 999 |
| Chr19_29877456 | Chr19 | 29877456 | 11.0835 | 137.121 | 17922 | 999 |
| Chr19_36151965 | Chr19 | 36151965 | 8.51082 | 93.0941 | 13762 | 999 |
| Chr19_41354272 | Chr19 | 41354272 | 12.0823 | 202.089 | 19537 | 999 |
| Chr19_44559738 | Chr19 | 44559738 | 8.45887 | 76.6024 | 13678 | 999 |
| Chr19_47123538 | Chr19 | 47123538 | 4.95857 | 28.7699 | 8018 | 999 |
| Chr19_47410875 | Chr19 | 47410875 | 13.8658 | 165.139 | 22421 | 999 |
| Chr19_47888068 | Chr19 | 47888068 | 5.93445 | 56.3757 | 9596 | 999 |
| Chr19_54054575 | Chr19 | 54054575 | 10.7644 | 211.699 | 17406 | 999 |
| Chr19_54054577 | Chr19 | 54054577 | 10.7965 | 212.713 | 17458 | 999 |
| Chr19_56101683 | Chr19 | 56101683 | 6.00124 | 62.5235 | 9704 | 999 |
| Chr19_63863784 | Chr19 | 63863784 | 34.3401 | 2221.49 | 55528 | 999 |
| Chr19_65111424 | Chr19 | 65111424 | 5.14657 | 37.0472 | 8322 | 999 |
| Chr19_66425994 | Chr19 | 66425994 | 7.50773 | 62.9518 | 12140 | 999 |
| Chr19_69923391 | Chr19 | 69923391 | 7.11503 | 58.79 | 11505 | 999 |
| Chr19_70010813 | Chr19 | 70010813 | 9.36054 | 141.561 | 15136 | 999 |
| Chr19_70166782 | Chr19 | 70166782 | 8.72542 | 95.1634 | 14109 | 999 |
| Chr19_70166794 | Chr19 | 70166794 | 8.80952 | 96.9946 | 14245 | 999 |
| Chr19_70746830 | Chr19 | 70746830 | 12.081 | 260.233 | 19535 | 999 |
| Chr19_70746840 | Chr19 | 70746840 | 12.0761 | 260.044 | 19527 | 999 |
| Chr19_73384447 | Chr19 | 73384447 | 27.4075 | 830.641 | 44318 | 999 |
| Chr19_78253132 | Chr19 | 78253132 | 15.4694 | 403.002 | 25014 | 999 |
| Chr19_83939793 | Chr19 | 83939793 | 22.4527 | 655.112 | 36306 | 999 |
| Chr19_83939823 | Chr19 | 83939823 | 18.0779 | 441.211 | 29232 | 999 |
| Chr19_85593766 | Chr19 | 85593766 | 35.5547 | 1526.91 | 57492 | 999 |
| Chr19_87285367 | Chr19 | 87285367 | 8.37291 | 87.0422 | 13539 | 999 |
| Chr19_87312336 | Chr19 | 87312336 | 6.16079 | 57.3627 | 9962 | 999 |
| Chr19_87312358 | Chr19 | 87312358 | 6.16698 | 57.409 | 9972 | 999 |
| Chr19_91261208 | Chr19 | 91261208 | 5.94682 | 60.7719 | 9616 | 999 |
| Chr19_99838811 | Chr19 | 99838811 | 8.49165 | 99.109 | 13731 | 999 |
| Chr19_100199603 | Chr19 | 100199603 | 7.36425 | 76.5362 | 11908 | 999 |
| Chr19_103514250 | Chr19 | 103514250 | 4.05318 | 21.0714 | 6554 | 999 |
| Chr19_104328619 | Chr19 | 104328619 | 6.41868 | 61.251 | 10379 | 999 |
| Chr19_104956756 | Chr19 | 104956756 | 7.24428 | 55.9025 | 11714 | 999 |
| Chr19_105614968 | Chr19 | 105614968 | 8.52381 | 117.783 | 13783 | 999 |
| Chr19_105614969 | Chr19 | 105614969 | 8.49969 | 117.035 | 13744 | 999 |
| Chr19_110231555 | Chr19 | 110231555 | 16.1064 | 351.875 | 26044 | 999 |
| Chr19_111448137 | Chr19 | 111448137 | 5.80148 | 37.1815 | 9381 | 999 |
| Chr19_114575641 | Chr19 | 114575641 | 10.4978 | 106.568 | 16975 | 999 |
| Chr19_115880922 | Chr19 | 115880922 | 21.3958 | 423.498 | 34597 | 999 |
| Chr19_121352039 | Chr19 | 121352039 | 10.8244 | 169.098 | 17503 | 999 |
| Chr19_121352068 | Chr19 | 121352068 | 11 | 172.537 | 17787 | 999 |
| Chr19_121358159 | Chr19 | 121358159 | 9.36797 | 63.5978 | 15148 | 999 |
| Chr19_122873076 | Chr19 | 122873076 | 7.42486 | 68.6108 | 12006 | 999 |
| Chr19_122873091 | Chr19 | 122873091 | 7.29375 | 65.7583 | 11794 | 999 |
| Chr19_130587502 | Chr19 | 130587502 | 15.0798 | 274.26 | 24384 | 999 |
| Chr19_130888543 | Chr19 | 130888543 | 6.59246 | 52.499 | 10660 | 999 |
| Chr19_130888562 | Chr19 | 130888562 | 6.56092 | 51.6264 | 10609 | 999 |
| Chr19_131376191 | Chr19 | 131376191 | 15.9276 | 354.886 | 25755 | 999 |
| Chr19_132935619 | Chr19 | 132935619 | 7.48052 | 71.6446 | 12096 | 999 |
| Chr19_136973381 | Chr19 | 136973381 | 8.10823 | 85.0594 | 13111 | 999 |
| Chr19_136973387 | Chr19 | 136973387 | 8.10575 | 84.6021 | 13107 | 999 |
| Chr19_137196212 | Chr19 | 137196212 | 7.90538 | 84.5387 | 12783 | 999 |
| Chr19_138185191 | Chr19 | 138185191 | 9.4026 | 87.8793 | 15204 | 999 |
| Chr19_138207186 | Chr19 | 138207186 | 12.8881 | 168.242 | 20840 | 999 |
| Chr19_138217702 | Chr19 | 138217702 | 22.4088 | 838.623 | 36235 | 999 |
| Chr19_138816657 | Chr19 | 138816657 | 5.1713 | 38.1507 | 8362 | 999 |
| Chr19_138816660 | Chr19 | 138816660 | 5.2585 | 39.7042 | 8503 | 999 |
| Chr19_138816709 | Chr19 | 138816709 | 5.41435 | 40.848 | 8755 | 999 |
| Chr19_142399592 | Chr19 | 142399592 | 9.8256 | 117.137 | 15888 | 999 |
| Chr19_144572698 | Chr19 | 144572698 | 20.8479 | 598.417 | 33711 | 999 |
| Chr19_146758725 | Chr19 | 146758725 | 14.3457 | 297.006 | 23197 | 999 |
| Chr19_153275094 | Chr19 | 153275094 | 5.80396 | 44.9411 | 9385 | 999 |
| Chr19_160855135 | Chr19 | 160855135 | 12.6815 | 225.434 | 20506 | 999 |
| Chr19_160869063 | Chr19 | 160869063 | 10.1305 | 128.291 | 16381 | 999 |
| Chr19_169305186 | Chr19 | 169305186 | 8.57885 | 81.9989 | 13872 | 999 |
| Chr19_169305200 | Chr19 | 169305200 | 8.57761 | 82.1179 | 13870 | 999 |
| Chr19_187126473 | Chr19 | 187126473 | 21.4199 | 851.771 | 34636 | 999 |
| Chr19_187126483 | Chr19 | 187126483 | 21.4626 | 853.891 | 34705 | 999 |
| Chr19_193290335 | Chr19 | 193290335 | 6.71923 | 54.8976 | 10865 | 999 |
| Chr19_193498691 | Chr19 | 193498691 | 11.603 | 155.008 | 18762 | 999 |
| Chr19_202071301 | Chr19 | 202071301 | 15.0872 | 205.696 | 24396 | 999 |
| Chr19_215827285 | Chr19 | 215827285 | 14.4657 | 271.119 | 23391 | 999 |
| Chr19_216602208 | Chr19 | 216602208 | 5.27829 | 30.5933 | 8535 | 999 |
| Chr19_220304114 | Chr19 | 220304114 | 16.7613 | 248.172 | 27103 | 999 |
| Chr19_232861300 | Chr19 | 232861300 | 7.43414 | 75.0713 | 12021 | 999 |
| Chr19_273148896 | Chr19 | 273148896 | 6.69821 | 51.9175 | 10831 | 999 |
| Chr19_284985486 | Chr19 | 284985486 | 28.1286 | 703.268 | 45484 | 999 |
| Chr19_291933611 | Chr19 | 291933611 | 6.38219 | 62.3179 | 10320 | 999 |
| Chr19_301630923 | Chr19 | 301630923 | 11.6302 | 217.264 | 18806 | 999 |
| Chr19_310807699 | Chr19 | 310807699 | 10.7656 | 116.105 | 17408 | 999 |
| Chr19_348076752 | Chr19 | 348076752 | 5.79963 | 36.8645 | 9378 | 999 |
| Chr19_356123485 | Chr19 | 356123485 | 27.7532 | 1285.52 | 44877 | 999 |
| Chr19_356123495 | Chr19 | 356123495 | 28.5417 | 1365.57 | 46152 | 999 |
| Chr19_358680257 | Chr19 | 358680257 | 12.3321 | 120.076 | 19941 | 999 |
| Chr19_395277747 | Chr19 | 395277747 | 6.07854 | 48.6603 | 9829 | 999 |
| Chr19_395277777 | Chr19 | 395277777 | 6.29808 | 51.2861 | 10184 | 999 |
| Chr19_402926408 | Chr19 | 402926408 | 11.7038 | 168.3 | 18925 | 999 |
| Chr19_433055725 | Chr19 | 433055725 | 4.62028 | 25.4906 | 7471 | 999 |
| Chr19_433055728 | Chr19 | 433055728 | 4.65244 | 25.8754 | 7523 | 999 |
| Chr19_433055744 | Chr19 | 433055744 | 4.53556 | 25.1524 | 7334 | 999 |
| Chr19_447006883 | Chr19 | 447006883 | 13.18 | 222.865 | 21312 | 999 |
| Chr19_482345530 | Chr19 | 482345530 | 20.0427 | 385.547 | 32409 | 999 |
| Chr19_507695492 | Chr19 | 507695492 | 4.20965 | 22.2141 | 6807 | 999 |
| Chr19_518297795 | Chr19 | 518297795 | 15.0662 | 294.147 | 24362 | 999 |
| Chr19_519918374 | Chr19 | 519918374 | 7.39579 | 69.1687 | 11959 | 999 |
| Chr19_525278254 | Chr19 | 525278254 | 28.5009 | 783.501 | 46086 | 999 |
| Chr19_540414037 | Chr19 | 540414037 | 9.80891 | 167.391 | 15861 | 999 |
| Chr19_542194753 | Chr19 | 542194753 | 16.5993 | 234.089 | 26841 | 999 |
| Chr19_551497220 | Chr19 | 551497220 | 4.02968 | 19.7986 | 6516 | 999 |
| Chr19_551502245 | Chr19 | 551502245 | 24.5065 | 549.739 | 39627 | 999 |
| Chr19_567392472 | Chr19 | 567392472 | 6.43414 | 52.2557 | 10404 | 999 |
| Chr19_567657205 | Chr19 | 567657205 | 13.9258 | 238.837 | 22518 | 999 |
| Chr19_568151844 | Chr19 | 568151844 | 13.4341 | 206.441 | 21723 | 999 |
| Chr19_568206980 | Chr19 | 568206980 | 17.8837 | 575.555 | 28918 | 999 |
| Chr19_579275771 | Chr19 | 579275771 | 6.77118 | 53.1481 | 10949 | 999 |
| Chr19_595874434 | Chr19 | 595874434 | 23.7551 | 1127.99 | 38412 | 999 |
| Chr19_600240707 | Chr19 | 600240707 | 6.43723 | 58.3217 | 10409 | 999 |
| Chr19_600240711 | Chr19 | 600240711 | 6.40074 | 57.2898 | 10350 | 999 |
| Chr19_611192837 | Chr19 | 611192837 | 4.45207 | 24.6229 | 7199 | 999 |
| Chr19_613982118 | Chr19 | 613982118 | 6.63327 | 65.2683 | 10726 | 999 |
| Chr19_621225169 | Chr19 | 621225169 | 19.9957 | 777.928 | 32333 | 999 |
| Chr19_621225181 | Chr19 | 621225181 | 17.7025 | 602.767 | 28625 | 999 |
| Chr19_626103249 | Chr19 | 626103249 | 5.765 | 50.0413 | 9322 | 999 |
| Chr19_633525948 | Chr19 | 633525948 | 28.7582 | 1393.73 | 46502 | 999 |
| Chr19_633526560 | Chr19 | 633526560 | 21.3302 | 356.853 | 34491 | 999 |
| Chr19_634868956 | Chr19 | 634868956 | 10.9165 | 172.348 | 17652 | 999 |
| Chr19_634868958 | Chr19 | 634868958 | 10.9703 | 174.185 | 17739 | 999 |
| Chr19_635571590 | Chr19 | 635571590 | 6.5102 | 44.3305 | 10527 | 999 |
| Chr19_639137052 | Chr19 | 639137052 | 5.50897 | 39.0756 | 8908 | 999 |
| Chr19_648383406 | Chr19 | 648383406 | 11.2962 | 162.754 | 18266 | 999 |
| Chr19_661055233 | Chr19 | 661055233 | 14.1608 | 341.359 | 22898 | 999 |
| Chr19_661221305 | Chr19 | 661221305 | 9.6543 | 95.5543 | 15611 | 999 |
| Chr19_667426316 | Chr19 | 667426316 | 13.4218 | 327.468 | 21703 | 999 |
| Chr19_667799520 | Chr19 | 667799520 | 10.2474 | 137.147 | 16570 | 999 |
| Chr19_667799542 | Chr19 | 667799542 | 10.2839 | 137.785 | 16629 | 999 |
| Chr19_667799559 | Chr19 | 667799559 | 10.4836 | 143.646 | 16952 | 999 |
| Chr19_667799567 | Chr19 | 667799567 | 10.4985 | 144.266 | 16976 | 999 |
| Chr19_670422192 | Chr19 | 670422192 | 14.4484 | 271.198 | 23363 | 999 |
| Chr19_670422199 | Chr19 | 670422199 | 14.4131 | 270.239 | 23306 | 999 |
| Chr19_670422209 | Chr19 | 670422209 | 14.4348 | 270.179 | 23341 | 999 |
| Chr19_674206491 | Chr19 | 674206491 | 8.40816 | 82.3061 | 13596 | 999 |
| Chr19_674206514 | Chr19 | 674206514 | 8.54298 | 83.2792 | 13814 | 999 |
| Chr19_683078725 | Chr19 | 683078725 | 22.6024 | 549.743 | 36548 | 999 |
| Chr19_685724565 | Chr19 | 685724565 | 20.9289 | 561.612 | 33842 | 999 |
| Chr19_688873494 | Chr19 | 688873494 | 11.2245 | 153.617 | 18150 | 999 |
| Chr19_692280728 | Chr19 | 692280728 | 7.83797 | 68.246 | 12674 | 999 |
| Chr19_699580116 | Chr19 | 699580116 | 17.2301 | 386.697 | 27861 | 999 |
| Chr19_702621705 | Chr19 | 702621705 | 9.8658 | 138.992 | 15953 | 999 |
| Chr19_706241533 | Chr19 | 706241533 | 5.88064 | 57.4567 | 9509 | 999 |
| Chr19_706241574 | Chr19 | 706241574 | 5.95238 | 58.533 | 9625 | 999 |
| Chr19_720869520 | Chr19 | 720869520 | 16.4317 | 335.913 | 26570 | 999 |
| Chr19_723458270 | Chr19 | 723458270 | 9.48299 | 73.1509 | 15334 | 999 |
| Chr19_725082995 | Chr19 | 725082995 | 37.7155 | 2570.12 | 60986 | 999 |
| Chr19_725082998 | Chr19 | 725082998 | 37.7001 | 2567.57 | 60961 | 999 |
| Chr19_761463377 | Chr19 | 761463377 | 7.16141 | 100.231 | 11580 | 999 |
| Chr19_761463432 | Chr19 | 761463432 | 7.0637 | 98.7837 | 11422 | 999 |
| Chr19_764651259 | Chr19 | 764651259 | 10.1571 | 130.578 | 16424 | 999 |
| Chr19_774682739 | Chr19 | 774682739 | 15.6809 | 423.742 | 25356 | 999 |
| Chr19_775136599 | Chr19 | 775136599 | 6.21954 | 56.9697 | 10057 | 999 |
| Chr19_775716841 | Chr19 | 775716841 | 8.21088 | 90.992 | 13277 | 999 |
| Chr19_778175485 | Chr19 | 778175485 | 9.39641 | 174.744 | 15194 | 999 |
| Chr19_778175506 | Chr19 | 778175506 | 9.52257 | 177.147 | 15398 | 999 |
| Chr19_781367377 | Chr19 | 781367377 | 15.7019 | 379.911 | 25390 | 999 |
| Chr19_783824174 | Chr19 | 783824174 | 25.2362 | 1081.96 | 40807 | 999 |
| Chr19_783824187 | Chr19 | 783824187 | 25.2641 | 1085.83 | 40852 | 999 |
| Chr19_786695798 | Chr19 | 786695798 | 8.42919 | 85.6709 | 13630 | 999 |
| Chr19_790474968 | Chr19 | 790474968 | 8.7149 | 88.225 | 14092 | 999 |
| Chr19_795472231 | Chr19 | 795472231 | 16.4199 | 258.98 | 26551 | 999 |
| Chr19_799378441 | Chr19 | 799378441 | 9.58009 | 73.8403 | 15491 | 999 |
| Chr19_799378447 | Chr19 | 799378447 | 9.52566 | 73.3634 | 15403 | 999 |
| Chr19_801514772 | Chr19 | 801514772 | 7.19171 | 55.7974 | 11629 | 999 |
| Chr19_801760984 | Chr19 | 801760984 | 13.7013 | 252.547 | 22155 | 999 |
| Chr19_801761017 | Chr19 | 801761017 | 13.0204 | 219.466 | 21054 | 999 |
| Chr20_276951 | Chr20 | 276951 | 6.70748 | 56.6477 | 10846 | 999 |
| Chr20_276984 | Chr20 | 276984 | 7.28139 | 64.3434 | 11774 | 999 |
| Chr20_1796623 | Chr20 | 1796623 | 4.33457 | 24.9047 | 7009 | 999 |
| Chr20_1976877 | Chr20 | 1976877 | 25.5102 | 703.489 | 41250 | 999 |
| Chr20_9114534 | Chr20 | 9114534 | 7.54174 | 77.4303 | 12195 | 999 |
| Chr20_9114554 | Chr20 | 9114554 | 7.54978 | 77.791 | 12208 | 999 |
| Chr20_14689895 | Chr20 | 14689895 | 7.13544 | 55.7521 | 11538 | 999 |
| Chr20_15437618 | Chr20 | 15437618 | 5.64502 | 45.4865 | 9128 | 999 |
| Chr20_16424776 | Chr20 | 16424776 | 7.83302 | 75.3706 | 12666 | 999 |
| Chr20_23736212 | Chr20 | 23736212 | 6.1577 | 47.8062 | 9957 | 999 |
| Chr20_23825693 | Chr20 | 23825693 | 11.4886 | 174.688 | 18577 | 999 |
| Chr20_27101299 | Chr20 | 27101299 | 18.094 | 291.059 | 29258 | 999 |
| Chr20_27101304 | Chr20 | 27101304 | 18.0223 | 288.242 | 29142 | 999 |
| Chr20_27101306 | Chr20 | 27101306 | 19.5955 | 372.864 | 31686 | 999 |
| Chr20_27902283 | Chr20 | 27902283 | 6.97093 | 59.3055 | 11272 | 999 |
| Chr20_28201301 | Chr20 | 28201301 | 10.4694 | 72.4101 | 16929 | 999 |
| Chr20_30102952 | Chr20 | 30102952 | 9.97835 | 150.256 | 16135 | 999 |
| Chr20_36560916 | Chr20 | 36560916 | 39.8448 | 1189.42 | 64429 | 999 |
| Chr20_41778219 | Chr20 | 41778219 | 6.05195 | 39.8426 | 9786 | 999 |
| Chr20_50818926 | Chr20 | 50818926 | 10.6914 | 193.999 | 17288 | 999 |
| Chr20_51426780 | Chr20 | 51426780 | 13.1107 | 340.224 | 21200 | 999 |
| Chr20_55559668 | Chr20 | 55559668 | 6.01484 | 60.8562 | 9726 | 999 |
| Chr20_74814898 | Chr20 | 74814898 | 7.22263 | 74.7722 | 11679 | 999 |
| Chr20_78052462 | Chr20 | 78052462 | 5.48856 | 40.1473 | 8875 | 999 |
| Chr20_78742054 | Chr20 | 78742054 | 11.0111 | 171.869 | 17805 | 999 |
| Chr20_78921690 | Chr20 | 78921690 | 26.6679 | 872.417 | 43122 | 999 |
| Chr20_79479473 | Chr20 | 79479473 | 13.7835 | 228.733 | 22288 | 999 |
| Chr20_81248047 | Chr20 | 81248047 | 8.51948 | 81.6421 | 13776 | 999 |
| Chr20_88811702 | Chr20 | 88811702 | 14.269 | 164.999 | 23073 | 999 |
| Chr20_94931299 | Chr20 | 94931299 | 5.97774 | 58.7619 | 9666 | 999 |
| Chr20_102958647 | Chr20 | 102958647 | 5.87941 | 49.491 | 9507 | 999 |
| Chr20_105464246 | Chr20 | 105464246 | 23.0204 | 678.296 | 37224 | 999 |
| Chr20_112164583 | Chr20 | 112164583 | 18.1058 | 432.632 | 29277 | 999 |
| Chr20_132291404 | Chr20 | 132291404 | 12.4947 | 280.487 | 20204 | 999 |
| Chr20_136775954 | Chr20 | 136775954 | 17.6605 | 542.632 | 28557 | 999 |
| Chr20_145384697 | Chr20 | 145384697 | 7.78726 | 70.8557 | 12592 | 999 |
| Chr20_145384700 | Chr20 | 145384700 | 7.75263 | 70.5254 | 12536 | 999 |
| Chr20_148322957 | Chr20 | 148322957 | 5.07978 | 32.6193 | 8214 | 999 |
| Chr20_171233211 | Chr20 | 171233211 | 6.99629 | 69.4369 | 11313 | 999 |
| Chr20_175929703 | Chr20 | 175929703 | 6.61719 | 66.4493 | 10700 | 999 |
| Chr20_176607293 | Chr20 | 176607293 | 10.8485 | 141.83 | 17542 | 999 |
| Chr20_176607304 | Chr20 | 176607304 | 10.8602 | 141.511 | 17561 | 999 |
| Chr20_193202832 | Chr20 | 193202832 | 11.0179 | 149.761 | 17816 | 999 |
| Chr20_197010368 | Chr20 | 197010368 | 10.1293 | 144.698 | 16379 | 999 |
| Chr20_197010420 | Chr20 | 197010420 | 10.2585 | 150.916 | 16588 | 999 |
| Chr20_200546262 | Chr20 | 200546262 | 10.9462 | 212.223 | 17700 | 999 |
| Chr20_209307441 | Chr20 | 209307441 | 11.0087 | 150.702 | 17801 | 999 |
| Chr20_209307444 | Chr20 | 209307444 | 10.8782 | 145.712 | 17590 | 999 |
| Chr20_209307458 | Chr20 | 209307458 | 11.1373 | 152.423 | 18009 | 999 |
| Chr20_209307459 | Chr20 | 209307459 | 11.1639 | 152.817 | 18052 | 999 |
| Chr20_209307461 | Chr20 | 209307461 | 11.1694 | 152.763 | 18061 | 999 |
| Chr20_209336851 | Chr20 | 209336851 | 8.25727 | 76.5179 | 13352 | 999 |
| Chr20_210224094 | Chr20 | 210224094 | 18.4199 | 522.7 | 29785 | 999 |
| Chr20_212647274 | Chr20 | 212647274 | 17.0841 | 423.331 | 27625 | 999 |
| Chr20_213068277 | Chr20 | 213068277 | 5.18306 | 38.5952 | 8381 | 999 |
| Chr20_217106143 | Chr20 | 217106143 | 5.91775 | 44.1089 | 9569 | 999 |
| Chr20_217128670 | Chr20 | 217128670 | 5.95176 | 53.2898 | 9624 | 999 |
| Chr20_217128718 | Chr20 | 217128718 | 6.00433 | 53.9338 | 9709 | 999 |
| Chr20_220979038 | Chr20 | 220979038 | 8.65615 | 96.6206 | 13997 | 999 |
| Chr20_230137061 | Chr20 | 230137061 | 4.50031 | 19.4197 | 7277 | 999 |
| Chr20_235654682 | Chr20 | 235654682 | 12.8442 | 186.349 | 20769 | 999 |
| Chr20_246239009 | Chr20 | 246239009 | 29.5275 | 1575.97 | 47746 | 999 |
| Chr20_246788143 | Chr20 | 246788143 | 24.9227 | 919.747 | 40300 | 999 |
| Chr20_247814315 | Chr20 | 247814315 | 8.76747 | 96.7763 | 14177 | 999 |
| Chr20_251282929 | Chr20 | 251282929 | 8.8287 | 144.527 | 14276 | 999 |
| Chr20_251282941 | Chr20 | 251282941 | 8.91342 | 149.242 | 14413 | 999 |
| Chr20_257574060 | Chr20 | 257574060 | 10.8454 | 125.337 | 17537 | 999 |
| Chr20_275817311 | Chr20 | 275817311 | 8.68213 | 131.172 | 14039 | 999 |
| Chr20_275817315 | Chr20 | 275817315 | 8.69264 | 131.641 | 14056 | 999 |
| Chr20_276513137 | Chr20 | 276513137 | 7.16883 | 85.1714 | 11592 | 999 |
| Chr20_276693054 | Chr20 | 276693054 | 5.3321 | 37.0474 | 8622 | 999 |
| Chr20_283792483 | Chr20 | 283792483 | 5.39889 | 44.3996 | 8730 | 999 |
| Chr20_284774255 | Chr20 | 284774255 | 11.7112 | 127.215 | 18937 | 999 |
| Chr20_285827461 | Chr20 | 285827461 | 5.59122 | 36.1577 | 9041 | 999 |
| Chr20_288177017 | Chr20 | 288177017 | 17 | 341.587 | 27489 | 999 |
| Chr20_290839449 | Chr20 | 290839449 | 9.27273 | 88.6217 | 14994 | 999 |
| Chr20_290839479 | Chr20 | 290839479 | 9.20965 | 87.3576 | 14892 | 999 |
| Chr20_291873266 | Chr20 | 291873266 | 7.00062 | 32.8001 | 11320 | 999 |
| Chr20_294187882 | Chr20 | 294187882 | 6.90785 | 47.3436 | 11170 | 999 |
| Chr20_296717880 | Chr20 | 296717880 | 8.30921 | 102.454 | 13436 | 999 |
| Chr20_297630739 | Chr20 | 297630739 | 17 | 576.001 | 27489 | 999 |
| Chr20_321016032 | Chr20 | 321016032 | 7.29746 | 72.4752 | 11800 | 999 |
| Chr20_325634572 | Chr20 | 325634572 | 25.1237 | 697.014 | 40625 | 999 |
| Chr20_325634577 | Chr20 | 325634577 | 25.0272 | 689.208 | 40469 | 999 |
| Chr20_332164287 | Chr20 | 332164287 | 19.8262 | 680.963 | 32059 | 999 |
| Chr20_337944351 | Chr20 | 337944351 | 28.9215 | 1467.6 | 46766 | 999 |
| Chr20_339143644 | Chr20 | 339143644 | 10.3445 | 202.388 | 16727 | 999 |
| Chr20_350521522 | Chr20 | 350521522 | 6.82931 | 81.6874 | 11043 | 999 |
| Chr20_350521547 | Chr20 | 350521547 | 6.79468 | 80.8328 | 10987 | 999 |
| Chr20_363430738 | Chr20 | 363430738 | 13.974 | 350.671 | 22596 | 999 |
| Chr20_363430739 | Chr20 | 363430739 | 13.9641 | 349.287 | 22580 | 999 |
| Chr20_378528138 | Chr20 | 378528138 | 21.4286 | 864.95 | 34650 | 999 |
| Chr20_379029374 | Chr20 | 379029374 | 14.1051 | 299.875 | 22808 | 999 |
| Chr20_379165953 | Chr20 | 379165953 | 17.3395 | 417.528 | 28038 | 999 |
| Chr20_379165974 | Chr20 | 379165974 | 17.4669 | 420.832 | 28244 | 999 |
| Chr20_379165975 | Chr20 | 379165975 | 17.4632 | 420.97 | 28238 | 999 |
| Chr20_392288831 | Chr20 | 392288831 | 7.82993 | 74.7514 | 12661 | 999 |
| Chr20_398394340 | Chr20 | 398394340 | 19.7396 | 597.308 | 31919 | 999 |
| Chr20_401945354 | Chr20 | 401945354 | 5.25108 | 39.4047 | 8491 | 999 |
| Chr20_414086864 | Chr20 | 414086864 | 9.09153 | 130.677 | 14701 | 999 |
| Chr20_414086869 | Chr20 | 414086869 | 9.11008 | 130.937 | 14731 | 999 |
| Chr20_414086897 | Chr20 | 414086897 | 9.08411 | 129.55 | 14689 | 999 |
| Chr20_419690267 | Chr20 | 419690267 | 7.88559 | 71.0296 | 12751 | 999 |
| Chr20_423668398 | Chr20 | 423668398 | 14.384 | 337.612 | 23259 | 999 |
| Chr20_423668436 | Chr20 | 423668436 | 14.1596 | 333.897 | 22896 | 999 |
| Chr20_424010620 | Chr20 | 424010620 | 7.33395 | 64.2721 | 11859 | 999 |
| Chr20_424010635 | Chr20 | 424010635 | 7.08905 | 59.8708 | 11463 | 999 |
| Chr20_424010636 | Chr20 | 424010636 | 7.1509 | 61.4166 | 11563 | 999 |
| Chr20_438403191 | Chr20 | 438403191 | 38.7464 | 1648.35 | 62653 | 999 |
| Chr20_440036613 | Chr20 | 440036613 | 6.36054 | 59.149 | 10285 | 999 |
| Chr20_440036638 | Chr20 | 440036638 | 7.37106 | 89.1828 | 11919 | 999 |
| Chr20_441916334 | Chr20 | 441916334 | 8.58503 | 87.2145 | 13882 | 999 |
| Chr20_448591789 | Chr20 | 448591789 | 13.9023 | 135.213 | 22480 | 999 |
| Chr20_453657843 | Chr20 | 453657843 | 18.7904 | 522.368 | 30384 | 999 |
| Chr20_455131258 | Chr20 | 455131258 | 6.57328 | 53.7324 | 10629 | 999 |
| Chr20_456336240 | Chr20 | 456336240 | 7.77365 | 66.1938 | 12570 | 999 |
| Chr20_456664854 | Chr20 | 456664854 | 13.3438 | 291.901 | 21577 | 999 |
| Chr20_473261859 | Chr20 | 473261859 | 10.6741 | 130.367 | 17260 | 999 |
| Chr20_473261860 | Chr20 | 473261860 | 10.7174 | 131.674 | 17330 | 999 |
| Chr20_473333286 | Chr20 | 473333286 | 11.4069 | 157.792 | 18445 | 999 |
| Chr20_474886604 | Chr20 | 474886604 | 13.5485 | 343.467 | 21908 | 999 |
| Chr20_477804591 | Chr20 | 477804591 | 7.39023 | 81.5908 | 11950 | 999 |
| Chr20_488953053 | Chr20 | 488953053 | 5.34756 | 42.4002 | 8647 | 999 |
| Chr20_488953080 | Chr20 | 488953080 | 5.4731 | 44.3225 | 8850 | 999 |
| Chr20_488953081 | Chr20 | 488953081 | 5.49165 | 44.8342 | 8880 | 999 |
| Chr20_492433825 | Chr20 | 492433825 | 4.35622 | 24.3619 | 7044 | 999 |
| Chr20_496453882 | Chr20 | 496453882 | 10.1682 | 143.766 | 16442 | 999 |
| Chr20_506125269 | Chr20 | 506125269 | 7.71923 | 66.9743 | 12482 | 999 |
| Chr20_511894301 | Chr20 | 511894301 | 19.0266 | 404.762 | 30766 | 999 |
| Chr20_518152221 | Chr20 | 518152221 | 21.6549 | 650.86 | 35016 | 999 |
| Chr20_520189512 | Chr20 | 520189512 | 18.0631 | 348.32 | 29208 | 999 |
| Chr20_523338183 | Chr20 | 523338183 | 12.9703 | 285.222 | 20973 | 999 |
| Chr20_523338209 | Chr20 | 523338209 | 12.8009 | 276.73 | 20699 | 999 |
| Chr20_523338210 | Chr20 | 523338210 | 12.8002 | 277.263 | 20698 | 999 |
| Chr20_529010511 | Chr20 | 529010511 | 7.84601 | 65.9608 | 12687 | 999 |
| Chr20_529320423 | Chr20 | 529320423 | 9.06679 | 114.179 | 14661 | 999 |
| Chr20_536961159 | Chr20 | 536961159 | 11.5832 | 222.377 | 18730 | 999 |
| Chr20_536961182 | Chr20 | 536961182 | 11.5083 | 219.045 | 18609 | 999 |
| Chr20_542457790 | Chr20 | 542457790 | 6.16388 | 56.1099 | 9967 | 999 |
| Chr20_542457824 | Chr20 | 542457824 | 7.19109 | 76.5953 | 11628 | 999 |
| Chr20_542457826 | Chr20 | 542457826 | 7.16698 | 75.7246 | 11589 | 999 |
| Chr20_542457842 | Chr20 | 542457842 | 7.20779 | 76.3132 | 11655 | 999 |
| Chr20_542457848 | Chr20 | 542457848 | 7.18244 | 76.0849 | 11614 | 999 |
| Chr20_556104297 | Chr20 | 556104297 | 16.308 | 280.748 | 26370 | 999 |
| Chr20_556978629 | Chr20 | 556978629 | 24.1027 | 998.643 | 38974 | 999 |
| Chr20_560589333 | Chr20 | 560589333 | 5.40012 | 32.3813 | 8732 | 999 |
| Chr20_561972174 | Chr20 | 561972174 | 8.10328 | 41.2882 | 13103 | 999 |
| Chr20_567217995 | Chr20 | 567217995 | 12.6636 | 261.965 | 20477 | 999 |
| Chr20_568849697 | Chr20 | 568849697 | 19.6401 | 577.711 | 31758 | 999 |
| Chr20_570217385 | Chr20 | 570217385 | 7.41311 | 89.4716 | 11987 | 999 |
| Chr20_571365034 | Chr20 | 571365034 | 6.094 | 50.0667 | 9854 | 999 |
| Chr20_571609498 | Chr20 | 571609498 | 8.33704 | 82.5355 | 13481 | 999 |
| Chr20_571793709 | Chr20 | 571793709 | 5.00866 | 32.047 | 8099 | 999 |
| Chr20_571793724 | Chr20 | 571793724 | 4.95238 | 31.6073 | 8008 | 999 |
| Chr20_571793725 | Chr20 | 571793725 | 4.99443 | 32.0513 | 8076 | 999 |
| Chr20_573093299 | Chr20 | 573093299 | 9.03649 | 68.5005 | 14612 | 999 |
| Chr20_574043988 | Chr20 | 574043988 | 6.28633 | 60.2614 | 10165 | 999 |
| Chr20_583107805 | Chr20 | 583107805 | 7.40569 | 58.4913 | 11975 | 999 |
| Chr20_583107806 | Chr20 | 583107806 | 7.34075 | 56.9401 | 11870 | 999 |
| Chr20_583569880 | Chr20 | 583569880 | 14.0371 | 301.302 | 22698 | 999 |
| Chr20_583569893 | Chr20 | 583569893 | 14.1336 | 306.91 | 22854 | 999 |
| Chr20_583600514 | Chr20 | 583600514 | 15.3074 | 245.446 | 24752 | 999 |
| Chr20_583767022 | Chr20 | 583767022 | 5.98516 | 49.173 | 9678 | 999 |
| Chr20_583767023 | Chr20 | 583767023 | 5.99443 | 49.539 | 9693 | 999 |
| Chr20_583767034 | Chr20 | 583767034 | 5.9932 | 49.2394 | 9691 | 999 |
| Chr20_583767056 | Chr20 | 583767056 | 5.92022 | 48.6564 | 9573 | 999 |
| Chr20_587687525 | Chr20 | 587687525 | 5.62028 | 46.0674 | 9088 | 999 |
| Chr20_589634138 | Chr20 | 589634138 | 5.11626 | 38.7389 | 8273 | 999 |
| Chr20_589684289 | Chr20 | 589684289 | 6.50835 | 57.8033 | 10524 | 999 |
| Chr20_589688546 | Chr20 | 589688546 | 14.8349 | 306.415 | 23988 | 999 |
| Chr20_592040452 | Chr20 | 592040452 | 25.2876 | 887.608 | 40890 | 999 |
| Chr20_600876201 | Chr20 | 600876201 | 9.17192 | 124.345 | 14831 | 999 |
| Chr20_600876241 | Chr20 | 600876241 | 8.80334 | 116.398 | 14235 | 999 |
| Chr20_603356555 | Chr20 | 603356555 | 9.63884 | 132.968 | 15586 | 999 |
| Chr20_604756773 | Chr20 | 604756773 | 9.624 | 153.818 | 15562 | 999 |
| Chr20_605110164 | Chr20 | 605110164 | 6.82375 | 49.0611 | 11034 | 999 |
| Chr20_605110166 | Chr20 | 605110166 | 6.82004 | 48.9496 | 11028 | 999 |
| Chr20_606686820 | Chr20 | 606686820 | 7.22635 | 57.82 | 11685 | 999 |
| Chr20_606829912 | Chr20 | 606829912 | 29.9394 | 1515.62 | 48412 | 999 |
| Chr20_610641516 | Chr20 | 610641516 | 12.3086 | 147.746 | 19903 | 999 |
| Chr20_610717256 | Chr20 | 610717256 | 5.83673 | 48.7394 | 9438 | 999 |
| Chr20_611491208 | Chr20 | 611491208 | 6.11936 | 43.724 | 9895 | 999 |
| Chr20_612125523 | Chr20 | 612125523 | 10.2486 | 175.702 | 16572 | 999 |
| Chr20_616959920 | Chr20 | 616959920 | 5.82189 | 51.8977 | 9414 | 999 |
| Chr20_619803122 | Chr20 | 619803122 | 8.51206 | 98.5062 | 13764 | 999 |
| Chr20_623165739 | Chr20 | 623165739 | 20.9678 | 663.568 | 33905 | 999 |
| Chr20_623165746 | Chr20 | 623165746 | 20.846 | 652.25 | 33708 | 999 |
| Chr20_623171966 | Chr20 | 623171966 | 18.7767 | 1000.1 | 30362 | 999 |
| Chr20_623171984 | Chr20 | 623171984 | 19.4261 | 1071.94 | 31412 | 999 |
| Chr20_623172032 | Chr20 | 623172032 | 103.308 | 7369.97 | 167049 | 999 |
| Chr20_623172034 | Chr20 | 623172034 | 103.456 | 7400.64 | 167288 | 999 |
| Chr20_640265816 | Chr20 | 640265816 | 12.7396 | 245.003 | 20600 | 999 |
| Chr20_640265823 | Chr20 | 640265823 | 12.9759 | 251.048 | 20982 | 999 |
| Chr20_640265843 | Chr20 | 640265843 | 13.0748 | 254.157 | 21142 | 999 |
| Chr20_640265845 | Chr20 | 640265845 | 13.0433 | 251.588 | 21091 | 999 |
| Chr20_640265850 | Chr20 | 640265850 | 13.1088 | 255.478 | 21197 | 999 |
| Chr20_644077989 | Chr20 | 644077989 | 13.5999 | 181.525 | 21991 | 999 |
| Chr20_644078069 | Chr20 | 644078069 | 12.4805 | 243.747 | 20181 | 999 |
| Chr20_647524338 | Chr20 | 647524338 | 26.449 | 1244.91 | 42768 | 999 |
| Chr20_648709396 | Chr20 | 648709396 | 15.5628 | 282.77 | 25165 | 999 |
| Chr20_648899815 | Chr20 | 648899815 | 4.98887 | 31.8452 | 8067 | 999 |
| Chr20_649319479 | Chr20 | 649319479 | 31.7019 | 1699.41 | 51262 | 999 |
| Chr20_650347896 | Chr20 | 650347896 | 19.4224 | 641.885 | 31406 | 999 |
| Chr20_650347906 | Chr20 | 650347906 | 19.846 | 671.379 | 32091 | 999 |
| Chr20_657323573 | Chr20 | 657323573 | 9.05875 | 115.443 | 14648 | 999 |
| Chr20_663875371 | Chr20 | 663875371 | 12.1521 | 277.505 | 19650 | 999 |
| Chr20_663875404 | Chr20 | 663875404 | 12.1763 | 276.328 | 19689 | 999 |
| Chr20_667784662 | Chr20 | 667784662 | 14.0192 | 331.075 | 22669 | 999 |
| Chr20_669609300 | Chr20 | 669609300 | 8.02536 | 85.5235 | 12977 | 999 |
| Chr20_669942277 | Chr20 | 669942277 | 16.9821 | 527.29 | 27460 | 999 |
| Chr20_671573708 | Chr20 | 671573708 | 10.2826 | 179.228 | 16627 | 999 |
| Chr20_671675183 | Chr20 | 671675183 | 25.2393 | 844.372 | 40812 | 999 |
| Chr20_671675189 | Chr20 | 671675189 | 25.2338 | 845.482 | 40803 | 999 |
| Chr20_671675218 | Chr20 | 671675218 | 24.8262 | 833.003 | 40144 | 999 |
| Chr20_673407385 | Chr20 | 673407385 | 26.1701 | 1115.59 | 42317 | 999 |
| Chr20_673407422 | Chr20 | 673407422 | 24.8479 | 990.885 | 40179 | 999 |
| Chr20_675507334 | Chr20 | 675507334 | 17.0204 | 555.828 | 27522 | 999 |
| Chr20_675507352 | Chr20 | 675507352 | 17.1082 | 555.491 | 27664 | 999 |
| Chr21_454334 | Chr21 | 454334 | 9.77427 | 101.639 | 15805 | 999 |
| Chr21_732413 | Chr21 | 732413 | 12.5189 | 145.677 | 20243 | 999 |
| Chr21_732436 | Chr21 | 732436 | 12.3915 | 143.977 | 20037 | 999 |
| Chr21_14662803 | Chr21 | 14662803 | 28.0161 | 869.35 | 45302 | 999 |
| Chr21_19461428 | Chr21 | 19461428 | 13.9004 | 148.608 | 22477 | 999 |
| Chr21_19865402 | Chr21 | 19865402 | 13.2047 | 170.943 | 21352 | 999 |
| Chr21_26597138 | Chr21 | 26597138 | 11.1868 | 88.7955 | 18089 | 999 |
| Chr21_26643023 | Chr21 | 26643023 | 8.04947 | 81.3936 | 13016 | 999 |
| Chr21_28667469 | Chr21 | 28667469 | 5.60606 | 40.3763 | 9065 | 999 |
| Chr21_33179362 | Chr21 | 33179362 | 14.4113 | 389.756 | 23303 | 999 |
| Chr21_36897443 | Chr21 | 36897443 | 7.69635 | 77.6249 | 12445 | 999 |
| Chr21_38788166 | Chr21 | 38788166 | 7.68893 | 68.9929 | 12433 | 999 |
| Chr21_40825737 | Chr21 | 40825737 | 8.23438 | 92.9754 | 13315 | 999 |
| Chr21_40848869 | Chr21 | 40848869 | 6.59802 | 33.9064 | 10669 | 999 |
| Chr21_60544353 | Chr21 | 60544353 | 7.33952 | 84.4806 | 11868 | 999 |
| Chr21_60544354 | Chr21 | 60544354 | 7.3791 | 85.2417 | 11932 | 999 |
| Chr21_60544389 | Chr21 | 60544389 | 7.17934 | 83.1225 | 11609 | 999 |
| Chr21_60544391 | Chr21 | 60544391 | 7.21892 | 83.832 | 11673 | 999 |
| Chr21_66711647 | Chr21 | 66711647 | 5.5269 | 36.6467 | 8937 | 999 |
| Chr21_66711713 | Chr21 | 66711713 | 5.94187 | 40.6785 | 9608 | 999 |
| Chr21_68705341 | Chr21 | 68705341 | 10.5578 | 136.077 | 17072 | 999 |
| Chr21_74619683 | Chr21 | 74619683 | 5.60915 | 47.3558 | 9070 | 999 |
| Chr21_76442731 | Chr21 | 76442731 | 7.53123 | 82.9051 | 12178 | 999 |
| Chr21_76442734 | Chr21 | 76442734 | 7.96413 | 94.724 | 12878 | 999 |
| Chr21_85074698 | Chr21 | 85074698 | 11.2845 | 171.972 | 18247 | 999 |
| Chr21_87224891 | Chr21 | 87224891 | 17.3828 | 565.293 | 28108 | 999 |
| Chr21_87411360 | Chr21 | 87411360 | 9.71676 | 113.389 | 15712 | 999 |
| Chr21_96991725 | Chr21 | 96991725 | 11.449 | 127.639 | 18513 | 999 |
| Chr21_109431388 | Chr21 | 109431388 | 8.32035 | 72.0236 | 13454 | 999 |
| Chr21_121191714 | Chr21 | 121191714 | 13.1645 | 290.277 | 21287 | 999 |
| Chr21_122029829 | Chr21 | 122029829 | 11.6524 | 208.416 | 18842 | 999 |
| Chr21_123081526 | Chr21 | 123081526 | 6.77737 | 65.4541 | 10959 | 999 |
| Chr21_123575900 | Chr21 | 123575900 | 5.76129 | 38.0135 | 9316 | 999 |
| Chr21_123575901 | Chr21 | 123575901 | 5.7384 | 37.6623 | 9279 | 999 |
| Chr21_123575908 | Chr21 | 123575908 | 5.73531 | 37.623 | 9274 | 999 |
| Chr21_126244973 | Chr21 | 126244973 | 6.52072 | 41.9589 | 10544 | 999 |
| Chr21_126303815 | Chr21 | 126303815 | 5.36982 | 40.7023 | 8683 | 999 |
| Chr21_126303831 | Chr21 | 126303831 | 5.37786 | 40.8082 | 8696 | 999 |
| Chr21_126303839 | Chr21 | 126303839 | 5.3927 | 41.2696 | 8720 | 999 |
| Chr21_126652312 | Chr21 | 126652312 | 5.1812 | 28.0655 | 8378 | 999 |
| Chr21_127180495 | Chr21 | 127180495 | 5.85714 | 33.6844 | 9471 | 999 |
| Chr21_127180505 | Chr21 | 127180505 | 5.85714 | 33.7153 | 9471 | 999 |
| Chr21_134271196 | Chr21 | 134271196 | 12.3265 | 158.013 | 19932 | 999 |
| Chr21_134271211 | Chr21 | 134271211 | 12.3123 | 157.412 | 19909 | 999 |
| Chr21_138111635 | Chr21 | 138111635 | 7.11565 | 77.9291 | 11506 | 999 |
| Chr21_142021065 | Chr21 | 142021065 | 5.94496 | 33.3652 | 9613 | 999 |
| Chr21_143551768 | Chr21 | 143551768 | 14.6654 | 323.952 | 23714 | 999 |
| Chr21_143551770 | Chr21 | 143551770 | 14.5288 | 315.311 | 23493 | 999 |
| Chr21_143551792 | Chr21 | 143551792 | 14.6463 | 322.6 | 23683 | 999 |
| Chr21_148423926 | Chr21 | 148423926 | 9.68398 | 114.246 | 15659 | 999 |
| Chr21_148423929 | Chr21 | 148423929 | 9.67718 | 113.512 | 15648 | 999 |
| Chr21_148586020 | Chr21 | 148586020 | 7.06184 | 61.6459 | 11419 | 999 |
| Chr21_165532187 | Chr21 | 165532187 | 30.2703 | 1435.36 | 48947 | 999 |

**Mean depth**: Mean read depth per locus averaged across all individuals; **Var depth**: mean read depth for each variant, i.e. number of reads that have mapped to this position; **Sum depth**: sum of read depth; **Qual**: marker quality score

**Supplementary Table S3**: Frequency of half-sibs that pollinated the 15 mother genets.

| **Mother** | **No. of progeny** | **No. of Unique Fathers that Pollinated this Mother** | **Count of Half-sibs as fathers** | **% of Half-sibs among all fathers** |
| --- | --- | --- | --- | --- |
| C418S0089 | 63 | 28 | 2 | 7.1 |
| C418S0206 | 65 | 46 | 3 | 6.5 |
| C418S0207 | 53 | 30 | 3 | 10.0 |
| C418S0259 | 61 | 40 | 2 | 5.0 |
| C418S0295 | 58 | 47 | 0 | 0.0 |
| C418S0296 | 50 | 40 | 1 | 2.5 |
| C418S0343 | 59 | 35 | 2 | 5.7 |
| C418S0455 | 65 | 41 | 3 | 7.3 |
| C418S0460 | 53 | 38 | 2 | 5.3 |
| C418S0482 | 72 | 41 | 3 | 7.3 |
| C418S0512 | 46 | 34 | 3 | 8.8 |
| C418S0527 | 54 | 34 | 3 | 8.8 |
| C418S0591 | 44 | 33 | 1 | 3.0 |
| C418S0598 | 56 | 43 | 2 | 4.7 |
| C418S0604 | 47 | 19 | 0 | 0.0 |
|  |  |  |  |  |
| Total | 846 | 549 | 30 | 82.1 |
| Mean |  | 36.6 | 2 | 5.5 |

**Supplementary Table S4:** Wind direction and speed during the week of IWG anthesis in 2019.

| **Wind Direction** | **Frequency** | **Average Wind Speed (km/h)** |
| --- | --- | --- |
| NE | 19 | 2.00 |
| ENE | 19 | 2.53 |
| NNE | 17 | 2.83 |
| ESE | 17 | 2.32 |
| N | 16 | 1.85 |
| SSE | 12 | 4.92 |
| SE | 12 | 3.88 |
| NNW | 10 | 2.54 |
| NW | 9 | 3.89 |
| WNW | 8 | 3.49 |
| SW | 7 | 6.98 |
| E | 7 | 2.08 |
| W | 5 | 0.55 |
| SSW | 4 | 3.22 |
| S | 4 | 6.68 |
| WSW | 2 | 5.71 |

**Supplementary Table S5:** Diversity indices of progeny obtained from all fathers and those obtained from half-sib fathers. Values with different letters, wherever applicable, are significantly different at *α* = 0.05 level according to the LSD test among the bins.

|  | **Progeny from all fathers** | | | | **Progeny from half-sib fathers** | | | |
| --- | --- | --- | --- | --- | --- | --- | --- | --- |
| **Pollination distance bin** | **Genetic Distance** | **Nei’s *D*** | **Shannon Diversity Index** | **Rarefaction** | **Genetic Distance** | **Nei’s *D*** | **Shannon Diversity Index** | **Rarefaction** |
| **m** |  |  | ***H*** |  |  |  | ***H*** |  |
| 0-3 | 0.36a | 0.0132ab | 2.66 | 7.4 | 0.36a | 0.0131a | 2.40 | 8.09 |
| 3-6 | 0.36a | 0.0133ab | 2.60 | 7.23 | 0.36a | 0.0130ab | 1.83 | 7.00 |
| 6-9 | 0.36a | 0.0131b | 2.56 | 7.16 | 0.34a | 0.0129ab | 0.69 | 2.00 |
| 9-12 | 0.36a | 0.0132ab | 2.62 | 7.57 | -- | -- | -- | -- |
| 12-15 | 0.36a | 0.0138a | 2.48 | 7.61 | -- | -- | -- | -- |
| 15-18 | 0.36a | 0.0135ab | 2.27 | 6.88 | -- | -- | -- | -- |
| 18-21 | 0.36a | 0.0133ab | 2.04 | 6.67 | -- | -- | -- | -- |
| 21-24 | 0.36a | 0.0134ab | 1.68 | 6 | -- | -- | -- | -- |
| 24-27 | 0.37a | 0.0133ab | 0.38 | 2 | -- | -- | -- | -- |

**Supplementary Figure S1**: Arrangement of 15 IWG mother plants (families) used in this study from the cycle 4 IWG breeding population (UMN_C4) at the University of Minnesota. Numbers and heatmap boxes indicate the number of half sibs sampled per family. Gray dots indicate potential father genets and black dots within yellow boxes indicate dead or missing plants in the nursery.


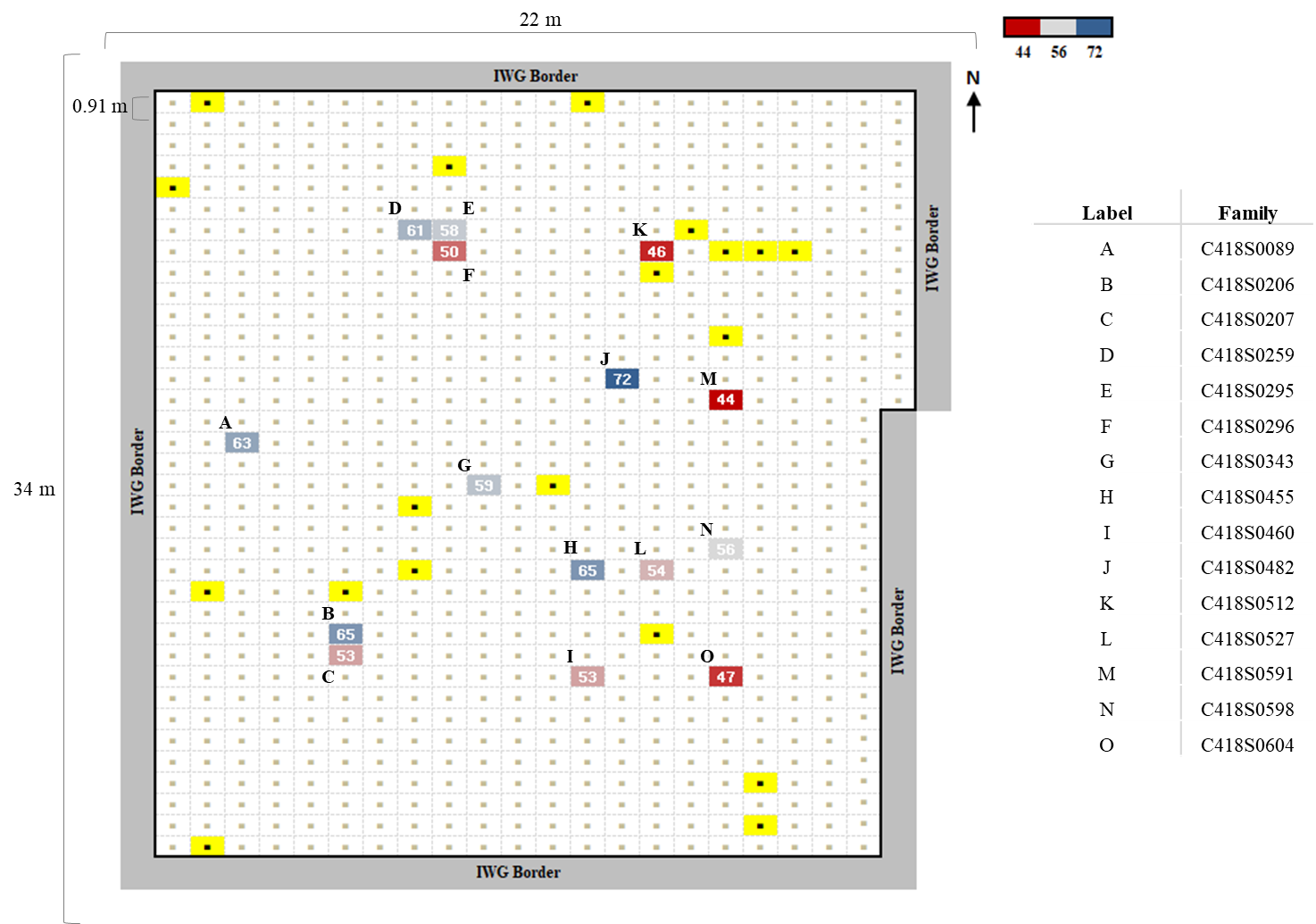


**Supplementary Figure S2:** Cardinal direction distribution of father plants in the nursery per mother plant.

**
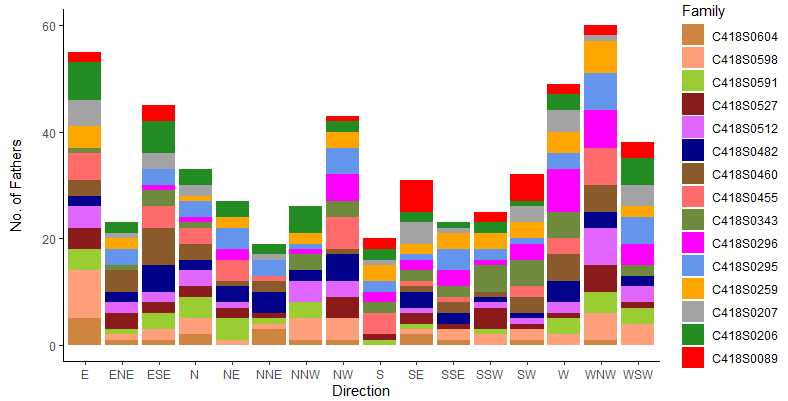
**

**Supplementary Figure S3:** Number of father genets that pollinated each mother plant and their distances from the mother plant.


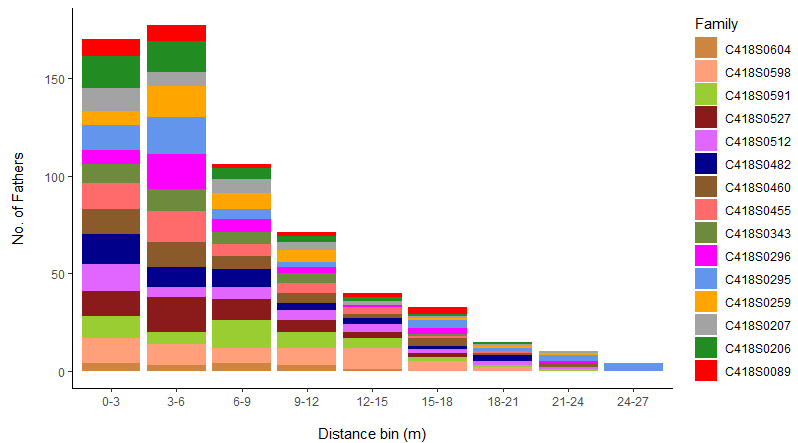

Supplement: Supplementary file 1 [file Data_Sheet_1.docx]
